# Conserved DNA Motifs, Including the CENP-B Box-like, Are Possible Promoters of Satellite DNA Array Rearrangements in Nematodes

**DOI:** 10.1371/journal.pone.0067328

**Published:** 2013-06-27

**Authors:** Nevenka Meštrović, Martina Pavlek, Ana Car, Philippe Castagnone-Sereno, Pierre Abad, Miroslav Plohl

**Affiliations:** 1 Department of Molecular Biology, Rudjer Bošković Institute, Zagreb, Croatia; 2 French National Institute for Agriculture Research (INRA), Institut Sophia Agrobiotech, Sophia Antipolis, France; 3 University of Nice- Sophia Antipolis (UNSA), UMR Institut Sophia Agrobiotech, Sophia Antipolis, France; 4 Centre National se la Recherche Scientifique (CNRS), Institut Sophia Agrobiotech, Sophia Antipolis, France; Oregon State University, United States of America

## Abstract

Tandemly arrayed non-coding sequences or satellite DNAs (satDNAs) are rapidly evolving segments of eukaryotic genomes, including the centromere, and may raise a genetic barrier that leads to speciation. However, determinants and mechanisms of satDNA sequence dynamics are only partially understood. Sequence analyses of a library of five satDNAs common to the root-knot nematodes *Meloidogyne chitwoodi* and *M. fallax* together with a satDNA, which is specific for *M. chitwoodi* only revealed low sequence identity (32–64%) among them. However, despite sequence differences, two conserved motifs were recovered. One of them turned out to be highly similar to the CENP-B box of human alpha satDNA, identical in 10–12 out of 17 nucleotides. In addition, organization of nematode satDNAs was comparable to that found in alpha satDNA of human and primates, characterized by monomers concurrently arranged in simple and higher-order repeat (HOR) arrays. In contrast to alpha satDNA, phylogenetic clustering of nematode satDNA monomers extracted either from simple or from HOR array indicated frequent shuffling between these two organizational forms. Comparison of homogeneous simple arrays and complex HORs composed of different satDNAs, enabled, for the first time, the identification of conserved motifs as obligatory components of monomer junctions. This observation highlights the role of short motifs in rearrangements, even among highly divergent sequences. Two mechanisms are proposed to be involved in this process, i.e., putative transposition-related cut-and-paste insertions and/or illegitimate recombination. Possibility for involvement of the nematode CENP-B box-like sequence in the transposition-related mechanism and together with previously established similarity of the human CENP-B protein and pogo-like transposases implicate a novel role of the CENP-B box and related sequence motifs in addition to the known function in centromere protein binding.

## Introduction

Satellite DNAs (satDNAs) can be briefly defined as DNA elements repeated in tandem. Often found as high-copy sequences underlying centromeres and broad pericentromeric regions, they rapidly achieve extreme diversity in nucleotide sequence, copy number, and organization in reproductively isolated groups of organisms (for review see [1]). Extensive studies of centromeric regions suggest coevolution of satDNAs and centromere-specific histone-like proteins leading to rapid evolution of centromeres and their rapid evolution is thought to be an intrinsic trigger of speciation [2].

SatDNAs evolve according to principles of concerted evolution, which is consequence of a 2-level process called molecular drive. At the first level, within the genome, mutations are homogenized among repeats of the satDNA [3]. Sequence homogenization results from a complex interplay of recombinational mechanisms, such as unequal crossing over and gene conversion. On the population level satDNA variants become fixed as a result of random assortment of genetic material in meiosis. The outcome of the whole process is higher homogeneity of repeats in a satDNA family within species than between species. Although turnover mechanisms in complex repetitive areas are difficult to explore, unequal crossing-over has been identified as the most widespread mechanism involved in satDNA dynamics in centromeric and pericentromeric regions [4], traditionally considered as regions of suppressed recombination (for review see [5]). Nevertheless, recent studies indicate gene conversion as the dominant mechanism in evolution of satDNAs [6]. As species diverge, satDNAs accumulate changes as a consequence of mutations and turnover mechanisms in separate lineages [3]. Rapidly accumulating differences in species satDNA profiles also can be accomplished by saltatory copy number changes and by emergence of new repeats in a common set or a library of satDNAs shared by related genomes [7], [8]. The library concept of satellite DNA evolution explains the occurrence of species-specific satellite DNA profiles as a result of differential amplifications and/or contractions within a pool of sequences shared by related genomes. In agreement with this concept, the study of 7 different satDNAs in six congeneric *Meloidogyne* species revealed the distribution of satDNAs consistent with lineage diversification and long term conservation (up to 45 Myr) of some satellite sequences [9].

However, the key question about satDNA evolution concerns the nature of mechanisms that drive formation and spread of novel tandem repeats in genomes. Although satDNAs can be extremely divergent, a common feature of many of them is irregular distribution of sequence variability along the monomer sequence and formation of conserved sequence segments, probably because of evolution under selective constraints (for review see [1]). The most prominent examples are found in rice [10], nematodes [8], *Arabidopsis* and human [11]. Among all detected conserved regions, the only function is assigned to the CENP-B box of alpha satDNA in human and other primates, which is proposed to act as a centromere protein binding site [12]. The possible role of other conserved sequence segments detected in satDNA monomers remains, however, obscure.

*Meloidogyne* are root-knot plant-parasitic nematodes that cause vast damage in agriculture. Although nematodes represent one large class of invertebrates, characterized by holocentric chromosomes with diffuse centromeres, evolutionary studies of satDNAs in this group are very limited. The recent completion of two root-knot nematode genomes *M. incognita* [13] and *M. hapla* [14] emphasized them as model organisms of metazoan plant parasitic species [15].

Recently separated but reproductively isolated nematodes *Meloidogyne fallax* and *M. chitwoodi* [16], [17] offer an exceptional platform to explore mechanisms involved in satDNA formation and spread and possible requirements on their sequences. Previous work showed six satDNAs in *M. chitwoodi*, grouped according to sequence similarity in group 1 (1a, 1b, 1c and 1d satDNAs) and group 2 (2a and 2b satDNAs) [18]. The presence of the conserved 2a satDNA in *M. fallax* [19] indicates distribution of these satellites according to the principles of the satDNA library concept [7].

In this paper, we characterized five divergent satDNAs of the library shared by *M. fallax* and *M. chitwoodi* and one satDNA which is specific for *M. chitwoodi* only. We performed structural, organizational and phylogenetic analyzes which disclosed complex organization patterns of monomers in the form of simple and higher-order repeat (HOR) arrays. We also detected two short conserved domains in analyzed satDNA sequences. Interestingly, one of them appeared to be similar to the CENP-B box of human alpha satDNA. It was detected in sequence alignments as a conserved segment common for six divergent satDNAs. Our results suggest involvement of conserved domains in array rearrangements and onset of new sequence combinations. Proposed mechanisms act on short-segment tracts and indicate highly recombinogenic nature of satDNA arrays. Based on our findings we suggest an additional role of the CENP-B box and general involvement of conserved sequence motifs in rapid evolution of tandemly repeated sequences.

## Materials and Methods

### Sampling and DNA Isolation

The *Meloidogyne* spp. isolates used in this study were chosen from the living collection maintained at INRA, Sophia Antipolis, France. The geographic origin of the isolates was as follows: *M. chitwoodi* (Spijkenisse, The Netherlands), *M. fallax* (Baexem, The Netherlands), *M. javanica* (Pelotas, Brazil), *M. paranaensis* (Londrina, Brazil), *M. incognita* (Antibes, France), *M. arenaria* (Chappes, France) and *M*. *hapla* (La Môle, France). Nematodes were maintained on tomatoes (*Lycopersicon esculentum* cv. Saint Pierre) grown at 20°C in a greenhouse. They were specifically identified morphologically and according to their isoesterase electrophoretic pattern [20]. Eggs were collected from infested roots, according to the procedure described earlier [21]. Total genomic DNA was purified from 50–100 µl eggs using the DNeasy Tissue Kit (Qiagen) according to the manufacturer’s instructions. Possibility of sample cross-contamination with other nematode DNA was excluded through PCR check of genomic DNA with SCAR (sequence characterized amplified region) primers specific for *Meloidogyne chitwoodi* and *M. fallax* species [22].

### PCR Analyses, Cloning and Sequencing

The satellite sequences were amplified with specific primers derived from previously published data [18] and from sequences obtained in this work. Primer sequences and their positions on the HOR sequence are indicated in Table S1 and Fig. S1, respectively. The reaction mixture consisted of reaction buffer, 1.5 mM MgCl_2_, 0.2 mM dNTPs, 0.5 U GoTaq DNA polymerase (Promega), 0.4 µM of each primer and 20 ng of genomic DNA. The PCR cycling parameters used were as follows: 2 min initial denaturation at 94°C, followed by 30 cycles of: 95°C for 30 sec, 58°C for 30 sec, and 72°C for 1 min. Final extension was at 72°C for 10 min. PCR products were ligated in a pGEM T-Easy vector (Promega) and transformed in *Escherichia coli* DH5α-competent cells (Invitrogene). Recombinant clones with multimeric arrays of satellite DNA were sequenced by Macrogen (Korea). Monomers and HORs sequences from *M. chitwoodi* and *M. fallax* as well as Box 1-containing sequences from *M. incognita* sequenced genome were deposited in EMBL databank under Accession Numbers: JX186757-JX186849, JX186850-JX186855, JX186856 - JX186877, JX186878 - JX186996, KC968979 - KC969073.

### Southern and Dot Blot Analyses

Standard procedures were used for restriction endonuclease digestions, electrophoresis, transfer to nylon membranes [23]. For genomic Southern hybridization analysis, 10 µg of genomic DNAs were partially digested with an appropriate restriction enzyme which cuts once in targeted repetitive unit. Gel electrophoresis was run in a 0.8% agarose, denatured and DNA transferred to Hybond N^+^membrane (Amersham). Hybridizations were performed overnight under high stringency conditions (65°C) in the buffer containing 250 mM Na_2_HPO_4_ (pH 7.2), 7% SDS, 1 mM EDTA, 0.5% blocking reagent and 50 ng/ml of the probe. Posthybridization washes were done in 20 mM Na_2_HPO_4_/1 mM EDTA/1% SDS at the temperature 2°C lower than the hybridization temperature. Chemiluminescent detection was carried out using the alkaline phosphatase substrate CDP-Star (Roche Applied Science). Cloned satellite monomers labeled with biotin-dUTP by PCR were used as hybridization probes.

The abundance of satDNA sequences in *Meloidogyne* species was estimated by quantitative dot blot analysis using a series of genomic DNA dilutions ranging from 50 to 200 ng. Satellite monomers, excised from a plasmid, were dot-blotted in the range between 0.05 and 1 ng, and used as a calibration curve.

### Sequence Analyses

DNA sequence data were compared to the GenBank databases by using the BLAST version 2.0 server at the National Center for Biotechnology Information.

The BLAST servers of *M. incognita* (http://meloidogyne.toulouse.inra.fr/blast/blast.html) and *M. hapla* (http://www.pngg.org/cbnp/index.php) genome were used to search for Box 1-containing sequences in the sequenced genomes. Initial sequence manipulations were done by using BioEdit v.7.0.5.3 [24]. Multiple alignments and pairwise sequence identity of monomers and HOR sequences were extracted from ClustalW Output (version 1.83) [25]. Lasergene software package v.7.0.0 (DnaStar) was used in further analyses of repetitive sequences including pairwise alignments, dot plot analyses and PCR primer design. Monomers from cloned multimeric arrays were extracted using Key-String Algorithm (KSA) [26]. KSA algorithm is based on the use of a freely chosen short sequence of nucleotides, called the key string, which cuts a given short sequence at each location within multimeric satDNA sequence. Distribution of monomer sequence variability was analyzed by using DnaSP v.4.10.9 [27]. The percent occurrence of the most frequent base at each site was calculated for all monomers repeats; this was plotted with the average percent occurrence and standard deviation (SD). A window length of 15 bp with a step size of 2 was used in the analysis. Due to the large number of monomers, neighbor-joining methods were used to construct phylogenetic tree by PAUP 4.0 (100 bootstrap iterations) [28]. Trees were displayed with MEGA 3.1 [29].

## Results

### Complex satDNA Arrays in *M. chitwoodi* and *M. fallax*

**Figure 1:**
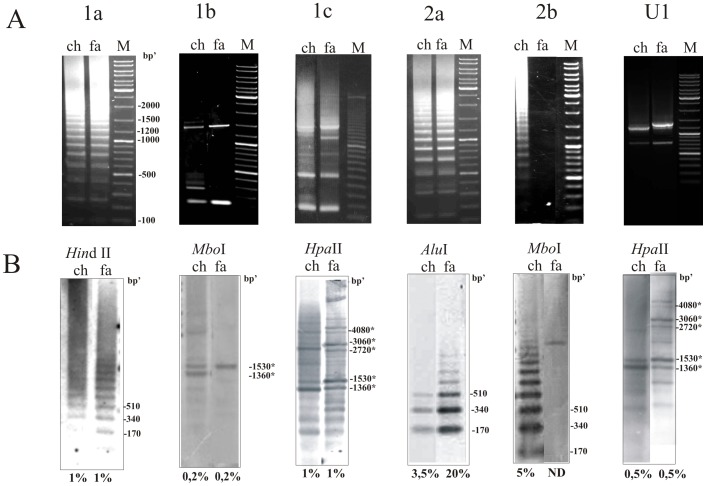
Organization of satDNAs in *M. chitwoodi* (ch) and *M. fallax* (fa) genome. (A) Electrophoretic separation of PCR products obtained by amplification of genomic DNAs using primers specific for 1a, 1b, 1c, 2a and 2b satDNAs and U1 sequence are shown on upper panel. (B) Southern hybridizations of genomic DNA partially digested with RE-s and probed with 1a, 1b, 1c, 2a and 2b satDNA monomers and with U1 sequence are shown on lower panel. Approximative contribution of particular sequence in the genome, estimated by dot blot, is shown as a percentage indicated below Southern blots. HORs are indicated with asteriks. M is the DNA ladder marker. ND-not detectable.

The first goal of this work was to characterize the structure and organization of satDNAs in *M. chitwoodi* and *M. fallax* genomes. PCR search for sequences related to *M. chitwoodi* satDNAs 1a, 1b, 1c, 1d, 2a and 2b revealed, except for 2b, orthologous counterparts in the closely related species *M. fallax* (Figure 1). However, it was not possible to detect any ortologous satDNA in other analysed *Meloidogyne* species (*M. incognita, M. javanica, M. arenaria, M. javanica, M*. *paranaensis*; data not shown).

**Figure 2:**
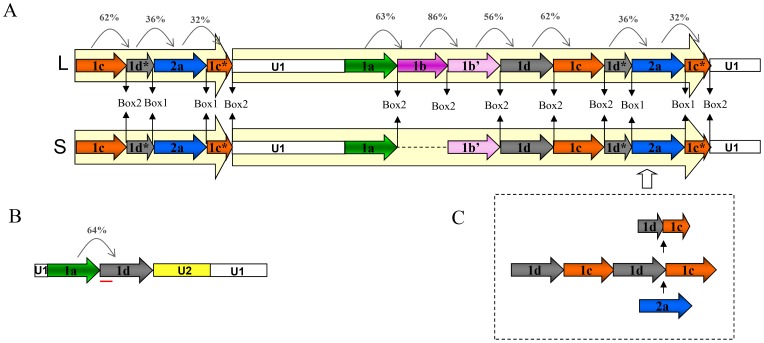
Schematic representation of complex satDNA structure. (A) The long-L and short-S HOR sequence and (B) complex fragment. The percent identity between monomers is written on arrows above the scheme. Box 1 and Box 2 in junction regions between different monomeres are indicated. 1d* and 1c* represent monomer parts which remain after 2a insertion. The red line below the complex fragment represents the overlapping segment of 1a and 1d monomers. (C) The scheme in the frame represents outcome of the proposed cut-and-paste mechanism of 2a insertion in HOR array.

Amplification of *M. chitwoodi and M. fallax* genomes with primers specific for satDNAs 1a, 2a and 2b produced ladder of bands based on the monomer size while other satDNA amplicons displayed different profiles (Figure 1A). PCR analysis of 1b showed bands of monomeric and dimeric size together with a fragment of about 1.5 kb in length, while amplification with 1c (Figure 1A) and 1d primers revealed complex but similar profiles (shown only for 1c). In order to perform detailed analyses of organizational patterns of these satDNA repeats, first we focused on sequences obtained by amplification of both genomes with 1c satDNA primers. In total, 20 cloned PCR fragments corresponding to multimeric size (i.e. ≥500 bp) were sequenced (Table S2). Two types of satDNA arrays were obtained, distinctive by the composition and organizational complexity of repeat subunits. The first type is characterized by arrays composed of alternating 1c and 1d satDNA monomers which together define the dimeric unit, 338 bp long (169 bp×2), organized in homogenous arrays (8 cloned fragments, M_1c_fa_n_ and M_1c_ch_n_; Table S1). Absence of a 170 bp based ladder in 1c PCR amplification supports this dimeric form as the basic repeating unit of these satDNAs. Multiple sequence alignment of another 12 fragments from *M. fallax* (H_1c_fa_n_) and *M. chitwoodi* (H_1c_ch_n_) (Table S2) revealed complex arrays composed of satDNA monomers 1a, 1b, a new 1b’ variant, 1c, 1d and 2a together with U1, yet uncharacterized sequence segment (Figure 2A and Figure S1). BLAST searches did not indicate any relevant sequence homology of U1 with the studied satDNAs or any other sequence deposited in data bases. In the following experiment, U1 specific PCR primers were constructed in order to extend the segments of complex arrays. In both genomes, obtained PCR products revealed fragments of expected lengths (∼1200 and ∼1400 bp) but also generated a shorter fragment of about 700 bp (Figure 1A). Sequence alignment of fragments obtained with U1 primers (H_u_ch_n_ and H_u_fa_n_) and previously cloned complex arrays (H_1c_fa_n_ and H_1c_ch_n_) is consistent with tandem organization of the HOR unit (Figure S1 and Figure 2A). In contrast to homogeneity of HOR units (84–99% mutual sequence identity) neighboring monomers in HORs show a wide range of relationships: from relatively high sequence identity of 86% between 1b and 1b’ variants to apparently unrelated sequences sharing only 32% identity, such as detected between 2a and 1c monomers (Figure 2A and Table 1). In addition, HOR segments revealed two variants which differ in the presence of 1b-type monomers (Figure 2A and Figure S1). Long HOR variants have two consecutive monomers, 1b and 1b’, that share sequence identity of 86% (Table 1), while short HOR variants lack 1b monomer (Figure 2A). Genomic DNA cut with the REs specific for 1c monomer sequence and probed with the labeled 1c monomer fragment supports the proposed HOR tandem organization (marked with asterisks in Figure 1B). Southern hybridization of genomic DNA with 1c indicates that long HOR variants prevail in *M. fallax* genome, while short variants seem to be more abundant in *M. chitwoodi* (Figure 1B).

**Table 1:** Mean of percent sequence identity between main groups of satDNAs monomers.

Monomer length(bp)	Monomers name	Group name	n	1aHch_n_, 1aHfa_n_	1aMch_n_, 1aMfa_n_	1bHch_n_ 1bHfa_n,_	1b’Hch_n_ 1b’Hfa_n_	1dHch_n_,1dHfa_n_, 1dMch_n_,1dMfa_n_	1cHch_n_, 1cHfa_n_, 1cMch_n_,1cMfa_n_	2aHch_n_,2aHfa_n_ 2aMch_n_, 2aMfa_n_	2bMch_n_
170	**1aHch_n_, 1aHfa_n_**	**1aH**	26	**94** (2.8)^a^							
169 (+5)	**1aMch_n_, 1aMfa_n_**	**1aM**	50	**81** (1.6)	**94** (2.1)						
170	**1bHch_n_, 1bHfa_n,_**	**1bH**	10	**63** (1.9)	**64** (0.8)	**99** (0.3)					
	**1b’Hch_n_ 1b’Hfa_n_**	**1b’H**	21	**64 (**1.2)	**66** (0.9)	**86**(2.9)	**93** (4.7)				
169	**1dHch_n_,1dHfa_n_, 1dMch_n_,1dMfa_n_**	**1dMH**	31	**64** (1.9)	**63** (2.0)	**57** (0.5)	**56** (0.7)	**98** (1.7)			
169	**1cHch_n_, 1cHfa_n_, 1cMch_n_,1cMfa_n_**	**1cMH**	31	**56** (0.6)	**53** (1.0)	**52** (0.2)	**51** (1.2)	**62** (1.0)	**99** (0.5)		
180	**2aHch_n_,2aHfa_n_ 2aMch_n_, 2aMfa_n_**	**2aMH**	37	**39** (0.9)	**40** (1.0)	**46** (0.6)	**42** (1.0)	**36** (0.7)	**32** (0.5)	**97** (1.6)	
179	**2bMch_n_**	**2bM**	10	**40** (1.1)	**40** (0.8)	**51** (0.5)	**46** (1.6)	**37** (0.6)	**35** (0.6)	**60** (0.9)	**96** (1.3)

n-number of analysed monomers.

a average percent identity scores for each pairwise comparison are indicated in bold, while standard deviation (SD) is indicated in bracket.

In addition to HORs, the alignment of the 700 bp-long complex fragments amplified with U1 primers revealed one additional homogenous group of sequences common for *M. fallax* and *M. chitwoodi*, named h_u_fa_n_ and h_u_ch_n_, respectively (Table S1). These sequences are composed of 1a and 1d complete monomers linked to a novel 170 bp long fragment named U2. The whole composite fragment is flanked by U1 sequences (Figure S2 and Figure 2B). It has to be noted that a 62 bp-long perfectly conserved fragment of U1 is also found as a part of U2 sequence. Additional PCR analyses using U2 specific primers could not prove tandem organization of the 700 bp complex fragment (data not shown). It can be therefore concluded that this fragment probably represents a particular combinatorial form of 1a and 1d satellite repeating units, present in the genome as an interspersed repeat.

### Homogenous Monomeric Arrays

PCR with 1a-specific primers produced ladder-like profiles in both genomes, with fragments corresponding to multimers of 170 bp (Figure 1A). Cloning and sequencing (Table S2) revealed homogenous tandem arrays (94% mutual identity) composed of a variant of 1a satDNA sequence, indicated now as 1aM. This variant is different from the HOR variant detected above, which is therefore indicated as 1aH. Average sequence identity between 1aH and 1aM variants is 81% (Table 1). Southern blot hybridization of genomic DNA probed with cloned 1aM-type satDNA repeats confirmed tandem organization of 1aM variants (Figure 1B). In addition, 1aH-specific primers were constructed to check if 1aH builds independent tandem arrays. PCR reaction did not reveal any ladder-like profile (data not shown) indicating that these variants are exclusively present as subunits of HORs.

We also examined if 1b variants could be found in monomeric arrays or are an exclusive component of HOR elements. Southern blot analysis of genomic DNA showed hybridization signals only in bands corresponding to HOR arrays (Figure 1B). PCR with 1b primers revealed fragments whose length corresponds to HOR organization. According to primer position, fragments of monomeric and dimeric forms that appeared in the PCR reaction (Figure 1A) originate from HORs, as they were undetectable in genomic Southern blot. These results emphasize unique organization of 1b monomers exclusively in HORs in both genomes.

**Figure 3:**
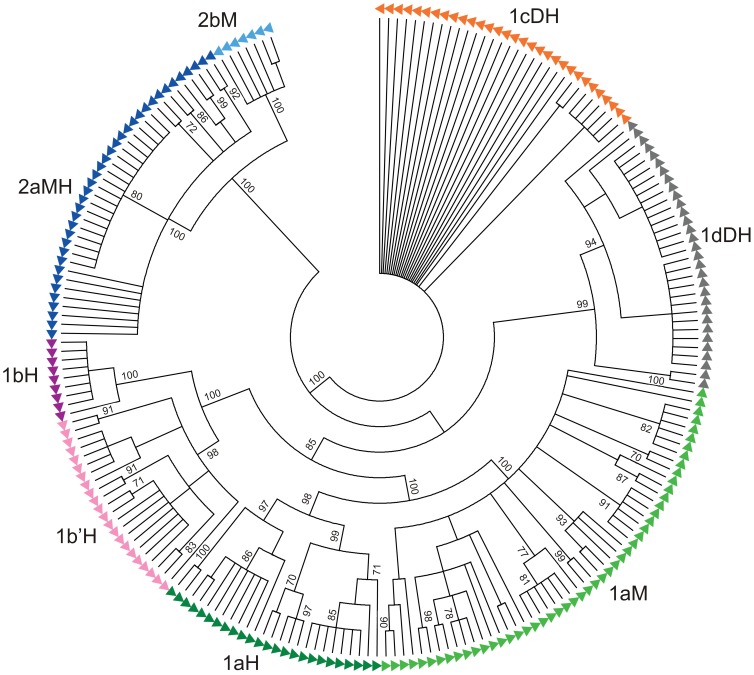
The phylogenetic tree of 1a, 1b, 1b’ 1c, 1d, 2a and 2b monomers. Monomers from the HORs (H), dimeric (D) and monomeric arrays (M). Phylogenetic analysis of 212 monomers was performed by neighbor-joining method with bootstrap value of 100. Numbers at nodes indicate bootstrap values (100 replicates; only values greater than 70 % are shown.

It has been published previously that 2a satDNA exists in the *M. fallax* and *M. chitwoodi* genome in tandem arrangement, in a high copy number, organized as homogenous monomeric arrays [19]. The results provided in this work show that 2a satellite also exists as the element of HORs in both genomes (Figure 2). No diagnostic sequence differences could be observed with respect to organizational pattern or species of origin (Figure 3 and Figure S3). The only difference is in abundance of 2a satDNA, 3.5% *in M. chitwoodi* and 20% in *M. fallax* (Figure 1). Examination of 2b satellite by PCR amplification and Southern blot (Figure 1) confirmed its exclusive presence in the *M. chitwoodi* genome in the form of high copy homogenous monomeric arrays. The only observed hybridization signal in *M. fallax* is the faint band (Figure 1B) which could represent a sporadic 2b sequence embedded in a longer DNA segment.

### Phylogenetic Analyzes of Monomers

In an effort to assess sequence dynamics of repetitive units in the closely related *M. chitwoodi* and *M. fallax* genomes, we examined phylogenetic relationships of all monomers, regardless to their organizational pattern and species origin. A total of 212 monomeric units from *M. chitwoodi* and *M. fallax* were included in the multiple sequence alignment (Figure S3). Neighbor-joining phylogenetic analysis showed eight different clusters (1aH, 1aM, 1bH, 1b’H, 1cDH, 1dDH, 2aMH and 2bM; letters H, D, M indicate HOR, dimeric or monomeric organizational form, respectively) distributed in two main branches, satDNAs of group 1 and group 2 (Figure 3). Monomers within clusters could not be distinguished according to the species of origin nor was it possible to differentiate 1c, 1d and 2a monomers according to their array affiliation. In agreement with previous observation, 1a split in 1aM and 1aH according to their organizational origin, while 1b monomers form two distinct groups 1bH and 1b’H related to their position in HORs. It should be noted that 1aH further clusters in two subgroups, based on short and long HOR forms.

Sequence comparisons between monomer groups display three different levels of similarity (Table 1). Similarity is high within 1bH group (86%) and between 1aM and 1aH (81%) monomer variants. Similarities within other satDNAs of group 1 and within satDNAs of group 2 are moderate, ranging from 51 to 66%. Comparison between satDNAs of group 1 and 2 gives negligible similarities, 32–46% (Table 1), and it can be supposed that these two groups might represent sequences of unrelated origin.

### Conserved Motifs and Junctions Between Monomers

**Figure 4:**
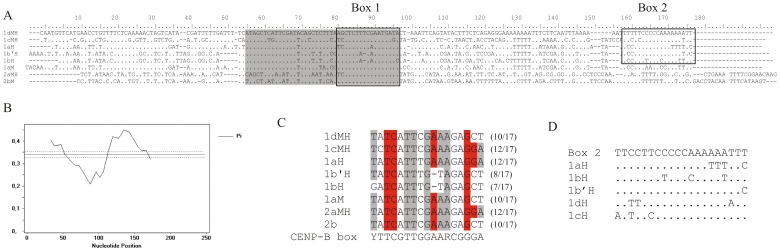
Conserved motifs in satDNAs. (A) Consensus sequences of 1dMH, 1cMH, 1aH, 1bH, 1b’H, 1aM, 2aMH and 2bM satDNAs, determined according to the 50% majority rule. Conserved Box 1 and Box 2 are indicated within the boxed area, and shaded part represents a region of low variability.(B) Identification of low variable domains by sliding window analysis by DnaSP. The average nucleotide variability P is shown by a solid line, and dashed lines represent 2-fold value of standard deviation. (C) Comparison of two variants of Box 1 with the consensus of human CENP-B box. The reverse complementary sequence of Box 1 is presented. Identities between sequences are highlighted in grey, and bases considered essential to bind the CENP-B protein in human [34] are highlighted in red. The number of total conserved bases is reported in brackets. (D) Aligment of Box 2 sequences from HOR related monomers; positions identical to the overall consensus are shown with dots.

In contrast to the very low overall sequence similarity between some of the monomer groups (Table 1), pairwise sequence alignment and sliding window analysis of all monomer sequences identified common domains of low variability (Figure 4A, B). The shaded domain in consensus sequences indicates the region of low variability shared among all satDNAs. Part of this region is a conserved 17 bp long segment, named Box 1. It is interesting to note that this sequence segment remains conserved among highly divergent satDNAs. For example, 1c and 2a satDNAs share only 32% identity while in the same time one single change characterizes the Box 1. Comparison of conserved Box 1 sequences (in Figure 4C presented as a reverse complement) with the human CENP-B box shows significant degree of similarity. Six of them have 10–12 out of 17 nucleotides conserved and if bases essential for CENP-B binding in human are considered, 4–5 out of 9 remain conserved. The lowest identity is in exclusively HOR-included elements, 1b’H and 1bH, in which sequences may represent degenerate variants of the motif. This analysis was extended with the search for related motifs in sequenced *M. incognita* and *M. hapla* genomes. Preliminary results recovered different repetitive sequences with the Box 1 in unassembled part of *M. incognita* sequenced genome (Figure S4). However, none of these repeats indicated any sequence similarity with satDNA sequences studied in this work in *M. chitwoodi* and *M. fallax*. In addition, detailed analysis of HOR elements in *M. chitwoodi* and *M. fallax* revealed that transitions from 1d to 2a monomer and from 2a to 1c are located exactly at the Box 1 (Figure 2A, see Discussion).

Another common region (Box 2) conserved in HOR-related monomers of group 1 satDNAs (1aH, 1bH, 1b’H, 1cH and 1dH) (Figure 4D and Figure S5). In order to refine alignment of this sequence motif, Box 2 segments were compared with their consensus sequence (Figure 4D). This region is 20 bp-long composed of T, C and A tracts and shows significant degree of mutual sequence identity with only few nucleotide changes (Figure 4D). It must be noted that the Box 2 region is always found in HORs as a transition region between monomers from group 1 (Figure 2A). In addition, detailed analysis of the so-called complex fragment revealed that 1a monomer extends into 1d monomer in the 50 bp long overlapping region shared by both monomers. This whole segment is highly conserved, with only 6 nucleotide substitutions (Figure S2).

## Discussion

In the present study we performed a comprehensive analysis of five divergent satDNAs (1a, 1b, 1c, 1d, and 2a) shared as elements of the satDNA library of root-knot nematodes *M. chitwoodi* and *M. fallax*, the two species considered to become separated recently [16], [17]. A distinctive element of this satDNA library is 2b satDNA, which turned to be present only in the *M. chitwoodi* genome. This observation supports our previous conclusion that presence of novel satDNAs in the library is accompany of speciation processes [9]. The distribution analysis data shows the absence of 1a, 1b, 1c, 1d, 2a and 2b counterparts in other congeneric *Meloidogyne* species thus indicating that satDNAs described in this work are specific for *M. chitwoodi* and M. *fallax*.

The exceptional attribute of studied satDNAs is complex organization of repeat units. Simple arrays are highly homogenous and composed of monomers or dimers, the later being built of two highly divergent monomers. Comparable dimeric organization based on monomers of low sequence similarity (50–60%) was reported in the marmoset (NewWorld monkeys) and it represents an ancient dimeric structure of alphoid sequences [30]. In our work, complex HORs are formed of monomers of divergent satDNAs that range from apparently unrelated (32% sequence identity) to those sharing up to 86% sequence identity. While the later can be considered as variants of a single satDNA, such as the 1b'H-1bH monomer pair, possible common evolutionary origin of the most divergent monomers is masked. Such a complex organization of monomers, described in details, is characteristic for alpha satDNA of human and great apes [30], [31]. For the difference to characterized nematode satDNAs, alpha satDNA HORs are composed of monomers with relatively high mutual sequence similarity (75–88%) [32]. A significant difference in organization of simple arrays can be also observed; while simple arrays of *M. fallax* and *M. chitwoodi* are highly homogenous (94–97% sequence similarity), equivalent arrays of alpha satDNA exhibit sequence similarity comparable to that of monomers in alpha HORs [32]. Phylogenetic analyses of alpha satDNA monomers in primates and human chategorized HOR and monomeric forms as phylogenetically distinct and suggested evolution of both forms from ancestral arrays of monomeric repeats [33]. Similar analysis in *M. chitwoodi* and *M. fallax* revealed clustering of HOR units with those from simple arrays, indicating continuous shuffling of monomers between HORs and simple arrays. The only exception is grouping of 1aH and 1aM monomers, in accordance with array affiliation. This result suggests that mechanisms in addition to unequal crossover over and gene conversion [3], [5] should be involved in creation of HORs (see below).

Irrespectively to the low level of sequence identity (32–64%) among studied satDNAs and the organizational pattern in which they were found, examined monomers share two conserved segments, named Box 1 and Box 2. Box 1 is a conserved 17 bp-long segment characteristic for all analyzed satDNAs. This particular motif is observed even in the divergent 2b satDNA, found only in homogeneous monomeric arrays of *M. chitwoodi*. One single deleted nucleotide was found in Box 1 of 1bH and 1b’H monomers which, curiously, appear exclusively as HOR-included elements. This raises the speculative possibility that conserved Box 1 participates in the formation of homogenous simple arrays. It was already proposed that abundant satDNAs may have been selected for amplification because of their ability to bind nuclear proteins [34]. Interestingly, conserved Box 1 shows significant homology with the human CENP-B box, with identity in 10–12 out of 17 nucleotides. The CENP-B box is a well-described sequence motif of human alpha satDNA which represents a binding site for the CENP-B protein in a subset of alpha satellite HORs [35]. It has been proposed that the CENP-B protein participates in human centromere assembly [35] but normal chromosome segregation in a mouse CENP-B protein null mutant and absence of CENP-B binding sites at the centromeres of human and mouse Y chromosome make its exact function unclear 36,37. DNA sequence motifs similar to the CENP-B box were found in diverse mammalian species, although their satDNA sequences are completely unrelated among themselves and with the alpha satDNA [38], [39]. For example, seven divergent horse satDNAs exibit CENP B box variants with identity in 9–12 out of 17 nucleotide of human CENP B box [39]. Presence of motifs similar to the CENP-B box has also been detected in a number of satDNAs from diverse species outside mammals [40], [41]. In examined nematode species, homology of Box 1 with the human CENP-B box is in the same range found for the CENP-B box in diverse mammalian species [39]. Exceptional feature of the nematode CENP-B box-like motif is significant conservation in the six divergent satDNAs which emphasized it as the most prominent example of the CENP-B box-like sequence out of mammals.

Mechanisms of genetic exchange of satDNAs are hard to study because of repetitive nature of satDNAs arrays. However, our experimental system composed of complex HORs and their counterparts in simple arrays offers a convenient model in which “beginning” and “end” of monomers can be precisely defined. Detailed analyses of *Meloidogyne* satDNA arrays led to observation that junctions between monomers are always located in conserved motifs. Box 1 is found at sites of insertion of the complete 2a monomer into highly divergent 1d and 1c monomers, while in turn, the corresponding segment of equivalent length in 1d and 1c, limited with Box 1, has been extruded (Figure 2C). This rearrangement event indicates novel cut-and-paste mechanism that involves the 17 bp-long CENP-B box-like motif and, probably, is related to mechanisms of transposition. It has been already hypothesized that the CENP-B box, in addition to its putative centromeric role, might have a function in satDNA sequence rearrangements [42]. This assumption is based on similarity of the CENP-B protein and transposases of the *pogo* family [43]. Accordingly, the CENP-B box might trigger illegitimate recombination in centromeric areas, in an epigenetically controlled process [44]. Highly conserved CENP-B protein homologs were detected in many mammalian species, but not in other metazoans [43]. In contrast, transposase-derived proteins related to the CENP-B and with putative ability to interact with satDNAs have been detected in diverse invertebrate and vertebrate species [43]. In support, a search in the genome sequence of related species *M. incognita* [13] allowed identification of an EST-supported gene encoding a protein with both CENP-B/Tc5 transposase DNA binding domains (Minc05185) (unpublished data) as well as the existence of different repetitive sequences that contain the CENP B box- like motif identical as that observed in this work.

The conserved Box 2 is a sequence motif composed of A/T/C tracts, found as a 20 bp- long transition region of all group 1 monomers in HORs. This indicates that homopolymeric tracts which have been found as a common feature of many satellites [1], participate in sequence recombination events in *Meloidogyne*. Since divergent monomers are involved, a mechanism of illegitimate recombination mediated by Box 2 can be assumed. Illegitimate recombination was previously proposed as a mechanism responsible for interspersion of long arrays generating abrupt switches between nonhomologous satDNAs in *Drosophila* [45]. While switches between unrelated arrays in *Drosophila* were detected as relatively rare events, our results nominate Box 2 as promoter of recombination acting frequently on DNA fragments of near monomer size. The minimal observed junction length of about 20 bp in both Box 1 and Box 2 is in accordance with the length of recombination breakpoints in human alpha-satellite [46]. In support to this, the role in satDNA shuffling can be assumed by presence of different conserved regions of similar length, as observed in the MEL 172 satDNA family identified in several *Meloidogyne* species [8] and in other, such as *Arabidopsis* [47].

In conclusion, we disclosed complex organization of monomers in two *Meloidogyne* species, characterized by highly homogenous simple arrays and by HORs, composed of highly divergent monomers. We propose that onset of this organizational pattern was mediated by conserved Box 1 and Box 2 sequence motifs. In principle, the two mechanisms are envisaged in this process, satDNA transposition and illegitimate recombination. Similarity of Box 1 with the CENP-B box of alpha satDNA and hypothesized transposase origin of the CENP-B protein [43] favor the role of transposition in formation and dynamics of satDNA arrays. These mechanisms act on short-segment tracts indicating the highly recombinogenic nature of repetitive environment which is in agreement with recent studies performed on mammalian centromere [44] and in other species [45], [48]. Finally, HORs can also represent a template from which monomers with conserved CENP-B box-like segments can be amplified and form high copy number arrays. It can be hypothesized that parallelism in organizational patterns of nematode and human satDNAs and similar sequence motif may mirror similar mechanisms of genesis and sequence dynamics, presumably driven by the same family of transposase-related processes.

## Supporting Information

Figure S1 **Alignment of HORs from** ***M. fallax*** **(clone names in blue) and** ***M. chitwoodi*** **(clone names in green).** H1cfa(n) and H1cch(n) represent fragments amplified with 1c primers. Hufa(n) and Huch(n) are amplified with primers specific for U1 sequence. All primer positions are marked above sequences and primers are listed in Table S1. SatDNA monomers are indicated in different colours; 1c, 1d, 2a, 1a, 1b and 1b'. Unlabeled part of the HOR is U1 sequence. Red boxes indicate Box A, and black boxes represent Box B. Sequences are deposited in EMBL databank under accession numbers: JX186856–JX186877.
(DOC)

Figure S2 **Alignment of complex fragments from** ***M. fallax*** **(clone names in blue) and** ***M. chitwoodi*** **(clone names in green).** Sequences are indicated in different colours; 1a monomer (green), 1d monomer (grey) and U2 sequence (yellow). Unlabeled part belongs to U1 sequence. Blue box represents overlapping region of 1a and 1d monomers. Box 1 is indicated in red, and Box 2 in black. Grey boxes represent perfectly conserved fragment common for U1 and U2 sequences. Primer positions for U2 are indicated above sequences. Sequences are deposited in EMBL databank under accession numbers: JX186850–JX186855.
(DOC)

Figure S3 **Alignment of 1a, 1b, 1b’, 1c, 1d, 2a and 2b monomers from** ***M. fallax*** **and** ***M. chitwoodi*** **.** Monomers are extracted from monomeric and HOR arrays using KSA algorithm [26]. All monomers are compared with first sequence and positions identical to the first sequence are shown with dot. Monomer group are indicated on the right side. Monomer sequences are deposited in EMBL data bank under accession numbers: JX186757–JX186849 and JX186878–JX186996. Box 1 is shaded with yellow. Detail description of satellite monomers are indicated below alignment.
(DOC)

Figure S4 **Alignment of Box 1-containing sequences extracted from unassembled part of** ***M. incognita*** **sequenced genome.** All sequences are compared with first sequence and positions identical to the first sequence are shown with dot. Sequences are deposited in EMBL data bank under accession numbers: KC968979–KC969073. Box 1 is shaded with yellow.
(DOC)

Figure S5 **Alignment of Box 2 from HOR related monomers of group 1 (1aH, 1bH, 1b’H1c, and 1dH).**
(DOC)

Table S1 **Primers used to amplify genomic sequences.**
(DOC)

Table S2 **Description of cloned satellite DNA arrays.** In cloned satellite fragments, letters H, M and h indicate higher-order repeats, monomeric arrays, complex fragment, respectively. Then follow primer name (first subscript), species acronym and clone number (second subscript).
(DOC)
