# Supplementary material for: Conserved DNA Motifs, Including the CENP-B Box-like, Are Possible Promoters of Satellite DNA Array Rearrangements in Nematodes
Source: PLoS One. 2013 Jun 27;8(6):e67328. doi: 10.1371/journal.pone.0067328 (PMC3694981; doi:10.1371/journal.pone.0067328)
Supplement: Figure S1 — Alignment of HORs from M. fallax (clone names in blue) and M. chitwoodi (clone names in green). H1cfa(n) and H1cch(n) represent fragments amplified with 1c primers. Hufa(n) and Huch(n) are amplified with primers specific for U1 sequence. All primer positions are marked above sequences and primers are listed in Table S1. SatDNA monomers are indicated in different colours; 1c, 1d, 2a, 1a, 1b and 1b'. Unlabeled part of the HOR is U1 sequence. Red boxes indicate Box A, and black boxes represent Box B. Sequences are deposited in EMBL databank under accession numbers: JX186856–JX186877. (DOC) [file pone.0067328.s001.doc]

**10 20 30 40 50 60 70 80 90 100 110 120 130 140 150 160 170 180 190 200**

1cL

**....|....|....|....|....|....|....|....|....|....|....|....|....|....|....|....|....|....|....|....|....|....|....|....|....|....|....|....|....|....|....|....|....|....|....|....|....|....|....|....|**

**H1cfa2 TTCGATTCACCTCTTCATCCTCTTTCGAATGAGATATGACTCATCAATAACTTACCTACAGAATGTTTTTTAAAAAACTCAAAGTATCCATTCTCCCCCCAAAAAATTTCTATGTTCCGGACCCTGTTTCTCAAAAACTAGTCATACGATTTTTGAATTTCATAGCACATTCGATTCAGCTTTCAAAGCTCTTTCGAATG**

**H1cfa8 ...N....N....................................C.............N....T....................................N...N..........N................................................NN.................................**

**H1cfa17 ..T..........................................C..................T.........................C...........................................................................T.................................**

**H1cfa18 .............................................C..................T.....................................................................................................T.................................**

**H1cch2 .............................................C.........T........T.....................................................A...........................T...................T.................................**

**H1cch3 ----T........................................C.........T........T.......................................................................................................................................**

**H1cch4 .............................................C.........T........T.....................................................................................................T.................................**

**H1cch6 .............................................C.........T........T.......................................................................................................................................**

**H1cch8 .............................................C.........T........T.....................................................................................................T......................A..........**

**H1cch9 .............................................C.........T........T.......................................................................................................................................**

**H1cch12 .............................................C.........T........T.....-...............................................................................................T.................................**

**H1cch11 .............................................C..................T.....................................................................................................T.................................**

**Huch21 --------------------------------------------------------------------------------------------------------------------------------------------------------------------------------------------------------**

**Huch22 --------------------------------------------------------------------------------------------------------------------------------------------------------------------------------------------------------**

**Huch23 --------------------------------------------------------------------------------------------------------------------------------------------------------------------------------------------------------**

**Huch11 --------------------------------------------------------------------------------------------------------------------------------------------------------------------------------------------------------**

**Hufa4 --------------------------------------------------------------------------------------------------------------------------------------------------------------------------------------------------------**

**Hufa1 --------------------------------------------------------------------------------------------------------------------------------------------------------------------------------------------------------**

**Hufa5 --------------------------------------------------------------------------------------------------------------------------------------------------------------------------------------------------------**

**HufaP8 --------------------------------------------------------------------------------------------------------------------------------------------------------------------------------------------------------**

**Huch9 --------------------------------------------------------------------------------------------------------------------------------------------------------------------------------------------------------**

**Hufa10 --------------------------------------------------------------------------------------------------------------------------------------------------------------------------------------------------------**

**210 220 230 240 250 260 270 280 290 300 310 320 330 340 350 360 370 380 390 400**

**....|....|....|....|....|....|....|....|....|....|....|....|....|....|....|....|....|....|....|....|....|....|....|....|....|....|....|....|....|....|....|....|....|....|....|....|....|....|....|....|**

**H1cfa2 ATATATGAATCATATAGAAATATTTTGTCGATAATAAGTTAGCCGCGGTTGAACCTCCCAAATTATTTTTTCGGAACAAGTCTGAAATTTTCGGAACAAGTCTCATAACTTACTGAATTTTCTT-CAAAAAATATTCATTCCAGCTTCAATATATTCAGAATTAAATCCTCTTTCGAATGATATATGACTCATACGAAAC**

**H1cfa8 ............................................................................................................................-...........................................................................**

**H1cfa17 ............................................................................................................................-...........................................................................**

**H1cfa18 ............................................................................................................................-...........................................................................**

**H1cch2 ............................................................................................................................-...........................................................................**

**H1cch3 ............................................................................................................................-...........................................................................**

**H1cch4 ............................................................................................................................-...........................................................................**

**H1cch6 ............................................................................................................................-...........................................................................**

**H1cch8 ............................................................................................................................-...........................................................................**

**H1cch9 ...........................................................G................................................................-...........................................................................**

**H1cch12 ............................................................................................................................-...........................................................................**

**H1cch11 ............................................................................................................................-...........................................................................**

**Huch21 --------------------------------------------------------------------------------------------------------------------------------------------------------------------------------------------------------**

**Huch22 --------------------------------------------------------------------------------------------------------------------------------------------------------------------------------------------------------**

**Huch23 --------------------------------------------------------------------------------------------------------------------------------------------------------------------------------------------------------**

**Huch11 --------------------------------------------------------------------------------------------------------------------------------------------------------------------------------------------------------**

**Hufa4 --------------------------------------------------------------------------------------------------------------------------------------------------------------------------------------------------------**

**Hufa1 --------------------------------------------------------------------------------------------------------------------------------------------------------------------------------------------------------**

**Hufa5 --------------------------------------------------------------------------------------------------------------------------------------------------------------------------------------------------------**

**HufaP8 --------------------------------------------------------------------------------------------------------------------------------------------------------------------------------------------------------**

**Huch9 --------------------------------------------------------------------------------------------------------------------------------------------------------------------------------------------------------**

**Hufa10 --------------------------------------------------------------------------------------------------------------------------------------------------------------------------------------------------------**

**410 420 430 440 450 460 470 480 490 500 510 520 530 540 550 560 570 580 590 600**

**....|....|....|....|....|....|....|....|....|....|....|....|....|....|....|....|....|....|....|....|....|....|....|....|....|....|....|....|....|....|....|....|....|....|....|....|....|....|....|....|**

**H1cfa2 -TTACCTACAGATTTTTTTTACAAAAATTCAAAATAGTCATTCTCCCCCAAAAAATTTCTATGTT-CAGCAACTTGCAACTTTTGACTGTTCTGTTAAAAGTTGATAATTTTTGCTTAAGAATGAAGAATATAGAATGAGAATCATAATAAAAGAAAAAAATTTGAAGAAATACCATAGAAATAAAAAGTTAATAAATTT**

**H1cfa8 -...............N..-.............................................-......................................................................N.......N.......................................................**

**H1cfa17 -................................................................-......................................................................................................................................**

**H1cfa18 -................................................................-......................................................................................................................................**

**H1cch2 -................................................................-.................................G............................................................................A.......................**

**H1cch3 -................................................................-.................................G............................................................................A.......................**

**H1cch4 -................................................................-.................................G............................................................................A.......................**

**H1cch6 -................................................................-.................................G............................................................................A.......................**

**H1cch8 -..........................C.....................................-.................................G............................................................................A.......................**

**H1cch9 -................................................................-.................................G...........................................................................GA.......................**

**H1cch12 -................................................................-.................................G............................................................................A.......................**

**H1cch11 -................................................................-.................K.G.............G............................................................................A.......................**

**Huch21 --------------------------------------------------------------------------------------------------------------------------------------------------------------------------------------------------------**

**Huch22 --------------------------------------------------------------------------------------------------------------------------------------------------------------------------------------------------------**

**Huch23 --------------------------------------------------------------------------------------------------------------------------------------------------------------------------------------------------------**

**Huch11 --------------------------------------------------------------------------------------------------------------------------------------------------------------------------------------------------------**

**Hufa4 --------------------------------------------------------------------------------------------------------------------------------------------------------------------------------------------------------**

**Hufa1 --------------------------------------------------------------------------------------------------------------------------------------------------------------------------------------------------------**

**Hufa5 --------------------------------------------------------------------------------------------------------------------------------------------------------------------------------------------------------**

**HufaP8 --------------------------------------------------------------------------------------------------------------------------------------------------------------------------------------------------------**

**Huch9 --------------------------------------------------------------------------------------------------------------------------------------------------------------------------------------------------------**

**Hufa10 --------------------------------------------------------------------------------------------------------------------------------------------------------------------------------------------------------**

**610 620 630 640 650 660 670 680 690 700 710 720 730 740 750 760 770 780 790 800**

U1L

**....|....|....|....|....|....|....|....|....|....|....|....|....|....|....|....|....|....|....|....|....|....|....|....|....|....|....|....|....|....|....|....|....|....|....|....|....|....|....|....|**

**H1cfa2 GTTCCCCCCATCTCCCTTTTTACACCCAGCCAAAACCCAAGCTCTCCAAAGTGGAAGTGGCTTGTCTCTTCTACACCTATTGAAGCTCTAATGCTTGGATGTGACCGTATCTGGTCTCTTCTGTACCTTTTGAAACTTAAAGGTTTGGAGTTTAATTTTTTATACACCCAATGAAACTTAAAGGTAGAAAAGGGTGTTCC**

**H1cfa8 .......S............................Y..........................................................................................................W........................................................**

**H1cfa17 ........................................................................................................................................................................................................**

**H1cfa18 ..............Y..............................................................................................................................................................................G.......A..**

**H1cch2 A.....................................................................................................................................G...............................T..........................A......**

**H1cch3 ......................................................................................................................................G.................................................................**

**H1cch4 ......................................................................................................................................G.................................................................**

**H1cch6 ......................................................................................................................................G.................................................................**

**H1cch8 ....................................A.................................................................................................G...........A.....................................................**

**H1cch9 ......................................................................................................................................G.................................................................**

**H1cch12 ......................................................................................................................................G.................................................................**

**H1cch11 ........................................................................................A...........A........................T-.......G.................................................................**

**Huch21 ---------------------------------------------------------------------------------------------------------------------------------------------------------------------------------------------...........**

**Huch22 ---------------------------------------------------------------------------------------------------------------------------------------------------------------------------------------------...........**

**Huch23 ---------------------------------------------------------------------------------------------------------------------------------------------------------------------------------------------...........**

**Huch11 ----------------------------------------------------------------------------------------------------------------------------------------------------------------------------------------------..........**

**HufaN4 ---------------------------------------------------------------------------------------------------------------------------------------------------------------------------------------------...........**

**HufaP1 ---------------------------------------------------------------------------------------------------------------------------------------------------------------------------------------------...........**

**HufaP4 ---------------------------------------------------------------------------------------------------------------------------------------------------------------------------------------------...........**

**HufaP8 ---------------------------------------------------------------------------------------------------------------------------------------------------------------------------------------------...........**

**HuchP9 ---------------------------------------------------------------------------------------------------------------------------------------------------------------------------------------------...........**

**HufaP10 ---------------------------------------------------------------------------------------------------------------------------------------------------------------------------------------------...........**

1aL

1aR

1aR'

U1R

**810 820 830 840 850 860 870 880 890 900 910 920 930 940 950 960 970 980 990 1000**

**....|....|....|....|....|....|....|....|....|....|....|....|....|....|....|....|....|....|....|....|....|....|....|....|....|....|....|....|....|....|....|....|....|....|....|....|....|....|....|....|**

**H1cfa2 TTT-ACTCCTTCTATTTTCAAAAATTTTTTT-CTCAAAAACTAGTCGATGGATTTTTGAATTT-CATAGCTCATTCGATTCAGCTCTTTACGCTCTTTCAAATGATACTAAATTCAGAAAAATTCCAACGATGGAAATTTTTTTATATAAGTTCAAAAATATTCCTTCCCCCAAATTTTTCTTCAAAAAATCATATCTCT**

**H1cfa8 ...-...........................-...............................-........................................................................................................................................**

**H1cfa17 ...-...........................-...............................-........................................................................................................................................**

**H1cfa18 ...-....Y...............A.....--...............................-T.........................A..........................C...T.........................A.......................A........--------------------**

**H1cch2 ...-....................A......T..............TG.A.......TTT.AAAT.A................TAA..C.TC.........................C...T..............C..........A..................C....A..-.....--------------------**

**H1cch3 ...T....................--.....T...............................-T..............A..........A........G.................C...T............R.T..........A..................C....A..-.....--------------------**

**H1cch4 ...-....................-......T...............................-T..................TAA..C.TC.........................C...T.........................A..T......T......................--------------------**

**H1cch6 ...-....................-......T...............................-T..................TAA..C.TC.........................C...T.........................A..T......T......................--------------------**

**H1cch8 ...-....................-......T...............................-..........................A...............TT.........C...T..............T..........A..................C....A..-.....--------------------**

**H1cch9 ...-....................-......T...............................-T..................TAA..C.TC.........................C...T.........................A..................-.............--------------------**

**H1cch12 ...-....................-......T...............................-T..................TAA..C.TC.........................C..MT.........................A..T......T......................--------------------**

**H1cch11 ...-....................A......-.........................TT-.AAAT.A................TAA..C.TC.........................C...T..............C..........A................................--------------------**

**Huch21 ...-...........................-...............................-T..................TAA..C.TC.........................C...T.........................A..T......T......................--------------------**

**Huch22 ...-...........................-...............................-T..................TAA..C.TC.........................C...T.........................A..T......T................-.....--------------------**

**Huch23 ...-....................A......-...............................-...................................................................................A..................-.............--------------------**

**Huch11 ...-...........................-...............................-T..................TAA..C.TC.........................C...T.........................A..T......T......................--------------------**

**HufaN4 ...-...........................-...............................-........................................................................................................................................**

**HufaP1 ...-...........................-...............................-........................................................................................................................................**

**HufaP4 ...-...........................-...............................-........................................................................................................................................**

**HufaP8 ...-....................AA.....-...............................-T.........................A..........................C...T.........................A.......................A............................**

**HuchP9 ...-....................AA.....-...............................-T.........................A..........................C...T.........................A.......................A............................**

**HufaP10 ...-...........................-...............................-........................................................................................................................................**

1bL

1bR

**1010 1020 1030 1040 1050 1060 1070 1080 1090 1100 1110 1120 1130 1140 1150 1160 1170 1180 1190 1200**

**....|....|....|....|....|....|....|....|....|....|....|....|....|....|....|....|....|....|....|....|....|....|....|....|....|....|....|....|....|....|....|....|....|....|....|....|....|....|....|....|**

**H1cfa2 CAAAGCCTTCTTATTTAGGAGGTCTATCATAGCTCATTCGATTCAGTTTTTCAAGCTCTACAAATGATCCAAAAATCAGCGAATATGATTCGAGCAACTTTTTTTTTTCAAACTTTGAAATATTCCTTCCCCCCAAAATTTTCAAAAATTTTTAAAAAATCATATCTTTCAAAGACTCTT---TTAGGAGGTCTATCAT**

**H1cfa8 .........................................................N.......................................................................T..................................................---................**

**H1cfa17 .................................................................................................................................T..................................................---C.......CT......**

**H1cfa18 ---------------------------------------------------------------------------------------------------------------------------------------------------------..............C......C..TC.TAT.....T..........**

**H1cch2 ---------------------------------------------------------------------------------------------------------------------------------------------------------..............C......C..TC.TAT.....T..........**

**H1cch3 ---------------------------------------------------------------------------------------------------------------------------------------------------------..............C......C..TC.TAT................**

**H1cch4 ---------------------------------------------------------------------------------------------------------------------------------------------------------..............C......C..TC.TAT................**

**H1cch6 ---------------------------------------------------------------------------------------------------------------------------------------------------------..............C......C..TC.TAT................**

**H1cch8 ---------------------------------------------------------------------------------------------------------------------------------------------------------..............C......C..TC.TAT................**

**H1cch9 ---------------------------------------------------------------------------------------------------------------------------------------------------------..............C...G..C..TC.TAT................**

**H1cch12 ---------------------------------------------------------------------------------------------------------------------------------------------------------..............C......C..TC.TAT................**

**H1cch11 ---------------------------------------------------------------------------------------------------------------------------------------------------------......C.......C......C..TC.TAT................**

**Huch21 ---------------------------------------------------------------------------------------------------------------------------------------------------------..............C......C..TC.TAT................**

**Huch22 ---------------------------------------------------------------------------------------------------------------------------------------------------------..............C......C..TC.TAT................**

**Huch23 ---------------------------------------------------------------------------------------------------------------------------------------------------------..............C......C..TC.TAT.....T..........**

**Huch11 ---------------------------------------------------------------------------------------------------------------------------------------------------------..............C......C..TC.TAT.....T..........**

**HufaN4 .................................................................................................................................T.....................................C......C.....T---...............**

**HufaP1 .................................................................................................................................T..................................................T---...............**

**HufaP4 .................................................................................................................................T..................................................T---...............**

**HufaP8 .................................................................................................................................T..................................................T---...............**

**HuchP9 .................................................................................................................................T..................................................T---...............**

**HufaP10 ....................................................G............................................................................T..................................................T---...............**

**1210 1220 1230 1240 1250 1260 1270 1280 1290 1300 1310 1320 1330 1340 1350 1360 1370 1380 1390 1400**

**....|....|....|....|....|....|....|....|....|....|....|....|....|....|....|....|....|....|....|....|....|....|....|....|....|....|....|....|....|....|....|....|....|....|....|....|....|....|....|....|**

**H1cfa2 AGCTCATTCGATTCAGTTTTTCGAGCTCTACAAATGATACAAATTACAGCGAATATGATTCGGAGGAAATTTTTT-ATCGAAGTTTTAAATATTCCTTCCCCCAAAAAATTCCAATGTTCATGGACTTGGTTCTCAAAAACTAGTCATACGATTTTTGAATA----------------------------------------**

**H1cfa8 ...........................................................................T.........................................N......N............................N.......TTCATAGNTCATTCGATACAGCTCTTTAANCTCTTTCGAAT**

**H1cfa17 ...........................................................................T...................................................................................T.TTCATAGCTCATTCGATACAGCTCTTTAAGCTCTTTCGAAT**

**H1cfa18 .........................................................G...C.............T...............................................................T........TAA......TTTA.AAATAGCTCATTCGATACAGCTTTTCATGCTCTTTCGAAT**

**H1cch2 .........................................................G...C.............T...................................................................................T.TTA-TAGCTCATTCGATACAGCTCTTTAAGCTCTTTCGAAT**

**H1cch3 .........................................................C...C.............T.......................................G...........................................T.TTCATAGCTCATTCGATACAGCTCTTTAAGCTCTTTCGAAT**

**H1cch4 .........................................................C...C.............T.......................................G...........................................T.TTCATAGCTCATTCGATACAGCTCTTTAAGCTCTTTCGAAT**

**H1cch6 .........................................................C...C.............T.......................................G...........................................T.TTCATAGCTCATTCGATACAGCTCTTTAAGCTCTTTCGAAT**

**H1cch8 .........................................................C...C.........C...T...................................................................................T.CT-ATAGCTCATTCGATACAGCTCTTTAAGCTCTTTCGAAT**

**H1cch9 .........................................................C...C.........-...T................N................C.................................................T.TTCATAGCTCATTCGATACAGCTCTTTAAGCTCTTTCGAAT**

**H1cch12 .........................................................C...C.............T...........T...........................G...........................................T.TTCATAGCTCATTCGATACAGCTCTTTAAGCTCTTTCGAAT**

**H1cch11 .........................................................C...C.............T.....................................................................................TTCATAG--CAATCGAAT-----------------------**

**Huch21 .........................................................C...C.............T...................T...................G...........................................T.TTCATAGCTCATTCGATACAGCTCTTTAAGCTCTTTCGAAT**

**Huch22 .........................................................C...C......G......T.......................................G...........................................T.TTCATAGCTCATTCGATACAGCTCTTTAAGCTCTTTCGAAT**

**Huch23 ...........................................................................T...................................................................................T.TTCATAGCTCATTCGATACAGCTCTTTAAGCTCTTTCGAAT**

**Huch11 ...........................................................................T.......................................G...........................................T.TTCATAGCTCATTCGATACAGCTCTTTAAGCTCTTTCGAAT**

**HufaN4 ...........................................................................T...................................................................................T.TTCATAGCTCATTCGATACAGCTCTTTAAGCTCTTTCGAAT**

**HufaP1 ...........................................................................T...................................................................................T.TTCATAGCTCATTCGATACAGCTCTTTAAGCTCTTTCGAAT**

**HufaP4 ...........................................................................T...................................................................................T.TTCATAGCTCATTCGATACAGCTCTTTAAGCTCTTTCGAAT**

**HufaP8 .........................................................C...C......G......T...............................................................T........TAA......TTTA.AAATAGCTCATTCGATACAGCTTTTCATGCTCTTTCGAAT**

**HuchP9 .........................................................C...C......G......T...............................................................T........TAA......TTTA.AAATAGCTCATTCGATACAGCTTTTCATGCTCTTTCGAAT**

**HufaP10 ...........................................................................T...................................................................................T.TTCATAGCTCATTCGATACAGCTCTTTAAGCTCTTTCGAAT**

**1410 1420 1430 1440 1450 1460 1470 1480 1490 1500 1510 1520 1530 1540 1550 1560 1570 1580 1590 1600**

**....|....|....|....|....|....|....|....|....|....|....|....|....|....|....|....|....|....|....|....|....|....|....|....|....|....|....|....|....|....|....|....|....|....|....|....|....|....|....|....|**

**H1cfa2 --------------------------------------------------------------------------------------------------------------------------------------------------------------------------------------------------------**

**H1cfa8 NATACTAAANTCAGTATACTTTCTCAGAGGGAAAAAAAATTTCTTCAATTTAAAAAANTTTTTCCCCCAAAAAANNTTNTATGTTCATGAACCTGTNTCTCGAGTTCTGTCNGTAAGTTGCTNGATNTCAATAGGTTGTTCGATTCACCTNTTCATCCTCTTTCGAATGAGATANGANTCATCCNTAACTTACCAA----**

**H1cfa17 GATACTAAATTCAGTATACTTTCTCAGAGGGAAAAAAAATTTCTTCAATTTAAAAAATTTTTTCCCCCAAAAAAATTTCTATGTTCATGAACCTGTTTCTCGAGTTCTGTCTGTAAGTTGCTTGATTTCAATAAA-----------------------------------------------------------------**

**H1cfa18 GATACTAAATTCAGTATACTTTCTCAGAGGGAAAAAAAATTTCTTCAATTTAAAAAATTTTTTCCCCCAAAAAAATTTCTATGTTCATGAACCTGTTTCTCGAGTTCTGTCTGTAAGTTGCTTGATTTCAATAGCTTGTTCGATTCACCTCTCCATCCTCTTTCGAATGAGATATGACTCATCCATAACTTACCTACAGA**

**H1cch2 GATACTAAATTCAGTATACTTTCTCAGAGGGAAAAAAAATTTCTTCAATTTAAAAAATTTTTTCCCCCAAAAAAATTTCTATGTTCATGAACCTGTTTCTCGAGTTCTGTCTGTAAGTTGCTTGATTTCAATAGCTTGTTCGATTCACCTCTTCATCCTCTTTCGAATGAGATATGACTCATCCATAACTTACCTACAGA**

**H1cch3 GATACTAAATTCAGTATACTTTCTCAGAGGGAAAAAAAATTTCTTCAATTTAAAAAATTTTTTCCCCCAAAAAAATTTCTATGTTCATGAACCTGTTTCTCGAGTTCTGTCTGTAAGTTGCTTGATTTCAATAGCTTGTTCGATTCACCTCTTCATCCTCTTTCGAATGAGAGATGACTCATCCATAACTTACTTACAGA**

**H1cch4 GATACTAAATTCAGTATACTTTCTCAGAGGGAAAAAAAATTTCTTCAATTTAAAAAATTTTTTCCCCCAAAAAAATTTCTATGTTCATGAACCTGTTTCTCGAGTTCTGTCTGTAAGTTGCTTGATTTCAATAGCTTGTTCGATTCACCTCTTCATCCTCTTTCGAATGAGATATGACTCATCCATAACTTACTTACAGA**

**H1cch6 GATACTAAATTCAGTATACTCTCTCAGAGGGAAAAAAAATTTCTTCAATTTAAAAAATTTTTTCCCCCAAAAAAATTTCTATGTTCATGAACCTGTTTCTCGAGTTCTGTCTGTAAGTTGCTTGATTTCAATAGCTTGCTCGATTCACCTCTTCATCCTCTTTCGAATGAGATATGACTCATCCATAACTTACCTACAGA**

**H1cch8 GATACTAAATTCAGTATACTTTCTCAGAGGGAAAAAAAATTTCTTCAATTTAAAAAATTTTTTCCCCCAAAAAAATTTCTATGTTCATGAACCTGTTTCTCGAGTTCTGTCTGTAAGTTGCTTGATTTCAATAGCTTGTTCGATTCACCTCTTCATCCTCTTTCGAATGAGATATGACTCATCCATAACTTACTTACAGA**

**H1cch9 GATACTAAATTCAGTATACTTTCTCAGAGGGAAAAAAA-TTTCTTCAATTTAAAAAATTTTTTCCCCCAAAAAAATTTCTGTGTTCATGAACCTGTTTCTCGAGTTCTGTCTGTAAGTTGCTTGATTTCAATAGCTTGTTCGATTCACCTCTTCATCCTCTTTCGAATGAGATATGACTCATCCATAACTTACCTACAGA**

**H1cch12 GATACTAAATTCAGTATACTTTCTCAGAGGGAAAAAAAATTTCTTCAATTTAAAAAATTTTTTCCCCCAAAAAAATTTCTATGTTCATGAACCTGTTTCTCGAGTTCTGTCTGTAAGTTGCTTGATTTCAATAGCTTGTTCGATTCACCTCTTCATCCTCTTTCGAATGAGATATGACTCATCCATAACTTACTTACAGA**

**H1cch11 --------------------------------------------------------------------------------------------------------------------------------------------------------------------------------------------------------**

**Huch21 GATACTAAATTCAGTATACTTTCTCAGAGGGAAAAAAAATTTCTTCAATTTAAAAAATTTTTTCCCCCAAAAAAATTTCTATGTTCATGAACCTGTTTCTCGAGTTCTGTCTGTAAGTTGCTTGATTTCAATAGCTTGTTCGATTCACCTCTTCATCCTCTTTCGAATGAGATATGACTCATCCATAACTTACTTACAGA**

**Huch22 GATACTAAATTCAGTATACTTTCTCAGAGGGAAAAAAAATTTCTTCAATTTAAAAAATTTTTTCCCCCAAAAAAATTTCTATGTTCATGAACCTGTTTCTCGAGTTCTGTCTGTAAGTTGCTTGATTTCAATAGCTTGTTCGATTCACCTCTTCATCCTCTTTCGAATGAGATATGACTCATCCATAACTTACCTACAGA**

**Huch23 GATACTAAATTCAGTATACTTTCTCAGAGGGAAAAAAAATTTCTTCAATTTAAAAAATTTTTTCCCCCAAAAAAATTTCTATGTTCATGAACCTGTTTCTCGAGTTCTGTCTGTAAGTTGCTTGATTTCAATAGCTTGTTCGATTCACCTCTTCATCCTCTTTCGAATGAGATATGACTCATCCATAACTTACCTACAGA**

**Huch11 GATACTAAATTCAGTATACTTTCTCAGAGGGAAAAAAAATTTCTTCAATTTAAAAAATTTTTTCCCCCAAAAAAATTTCTATGTTCATGAACCTGTTTCTCGAGTTCTGTCTGTAAGTTGCTTGATTTCAATAGCTTGTTCGATTCACCTCTTCATCCTCTTTCGAATGAGATATGACTCATCCATAACTTACTTACAGA**

**HufaN4 GATACTAAATTCAGTATACTTTCTCAGAGGGAAAAAAAATTTCTTCAATTTAAAAAATTTTTTCCCCCAAAAAAATTTCTATGTTCATGAACCTGTTTCTCGAGTTCTGTCTGTAAGTTGCTTGATTTCAATAGCTTGTTCGATTCACCTCTTCATCCTCTTTCGAATGAGATATGACTCATCCATAACTTACCTACAGA**

**HufaP1 GATACTAAATTCAGTATACTTTCTCAGAGGGAAAAAAAATTTCTTCAATTTAAAAAATTTTTTCCCCCAAAAAAATTTCTATGTTCATGAACCTGTTTCTCGAGTTCTGTCTGTAAGTTGCTTGATTTCAATAGCTTGTTCGATTCACCTCTTCATCCTCTTTCTAATGAGATATGACTCATCCATAACTTACCTACAGA**

**HufaP4 GATACTAAATTCAGTATACTTTCTCAGAGGGAAAAAAAATTTCTTCAATTTAAAAAATTTTTTCCCCCAAAAAAATTTCTATGTTCATGAACCTGTTTCTCGAGTTCTGTCTGTAAGTTGCTTGATTTCAATAGCTTGTTCGATTCACCTCTTCATCCTCTTTCTAATGAGATATGACTCATCCATAACTTACCTACAGA**

**HufaP8 GATACTAAATTCAGTATACTTTCTCAGAGGGAAAAAAAATTTCTTCAATTTAAAAAATTTTTTCCCCCAAAAAAATTTCTATGTTCATGAACCTGTTTCTCGAGTTCTGTCTGTAAGTTGCTTGATTTCAATAGCTTGTTCGATTCACCTCTTCATCCTCTTTCGAATGAGATATGACTCATCCATAACTTACCTACAGA**

**HuchP9 GATACTAAATTCAGTATACTTTCTCAGAGGGAAAAAAAATTTCTTCAATTTAAAAAATTTTTTCCCCCAAAAAAATTTCTATGTTCATGAACCTGTTTCTCGAGTTCTGTCTGTAAGTTGCTTGATTTCAATAGCTTGTTCGATTCACCTCTTCATCCTCTTTCGAATGAGATATGACTCATCCATAACTTACCTACAGA**

**HufaP10 GATACTAAATTCAGTATACTTTCTCAGAGGGAAAAAAAATTTCTTCAATTTAAAAAATTTTTTCCCCCAAAAAAATTTCTATGTTCATGAACCTGTTTCTCGAGTTCTGTCTGTAAGTTGCTTGGTTTCAATAGCTTGTTCGATTCACCTCTTCATCCTCTTTCGAATGAGATATGACTCATCCATAACTTACCTACAGA**

1cR

2aL

**1610 1620 1630 1640 1650 1660 1670 1680 1690 1700 1710 1720 1730 1740 1750 1760 1770 1780 1790 1800**

**....|....|....|....|....|....|....|....|....|....|....|....|....|....|....|....|....|....|....|....|....|....|....|....|....|....|....|....|....|....|....|....|....|....|....|....|....|....|....|....|**

**H1cfa2 --------------------------------------------------------------------------------------------------------------------------------------------------------------------------------------------------------**

**H1cfa8 --------------------------------------------------------------------------------------------------------------------------------------------------------------------------------------------------------**

**H1cfa17 --------------------------------------------------------------------------------------------------------------------------------------------------------------------------------------------------------**

**H1cfa18 ATTTTTTTTAAAAAACTCA-------------------------------------------------------------------------------------------------------------------------------------------------------------------------------------**

**H1cch2 ATTTTTTTTAAAAAACTCAAAGTATCCATTCTCCCCCC------------------------------------------------------------------------------------------------------------------------------------------------------------------**

**H1cch3 ATTTTTTTTAAAAAACTCAAAGTATCCATTCTCCCCCC------------------------------------------------------------------------------------------------------------------------------------------------------------------**

**H1cch4 ATTTTTTTTAAAAAACTCAAAGTATCCATTCTCCCCCC------------------------------------------------------------------------------------------------------------------------------------------------------------------**

**H1cch6 ATTTTTTTTAAAAAACTCAAAGTATCCATTCTCCCCCC------------------------------------------------------------------------------------------------------------------------------------------------------------------**

**H1cch8 ATTTTTTTTAAAAAACTCAAAGTATCCATTCTCCCCCC------------------------------------------------------------------------------------------------------------------------------------------------------------------**

**H1cch9 ATTTTTTTTAAAAAACTCAAAGTATCCATTCTCCCCCC------------------------------------------------------------------------------------------------------------------------------------------------------------------**

**H1cch12 ATTTTTTTTAAAAAACTCAAAGTATCCATTCTCCCCCC------------------------------------------------------------------------------------------------------------------------------------------------------------------**

**H1cch11 --------------------------------------------------------------------------------------------------------------------------------------------------------------------------------------------------------**

**Huch21 ATTTTTTTTAAAAAACTCAAAGTATCCATTCTCCCCCCAAAAAATTTCTATGTTCCGGACCCTGTTTCTCAAAAACTAGTCATACGA-TTTTTGAATTTCATAGCTCATTCGATTCAGCTTTCAAAGCTCTTTCGAATGATATATGAATCATATAGAAATATTTTGTCGATAATAAGTTAGCCGCGGTTGAACCTCCCAA**

**Huch22 ATTTTTTTTAAAAAACTCAAAGTATCCATTCTCCCCCCAAAAAATTTCTATGTTCCGGACCCTGTTTCTCAAAAACTAGTCATACGA-TTTTTGAATTTCATAGCTCATTCGATTCAGCTTTCAAAGCTCTTTCGAATGATATATGAATCATATAGAAATATTTTGTCGATAATAAGTTAGCCGCGGTTGAACCTCCCAA**

**Huch23 ATTTTTTTTAAAAAACTCAAAGTATCCATTCTCCCCCCAAAAAATTTCTATGTTCCGGACCCTGTTTCTCAAAAACTAGTCATACGA-TTTTTGAATTTCATAGCTCATTCGATTCAGCTTTCAAAGCTCTTTCGAATGATATATGAATCATATAGAAATATTTTGTCGATAATAAGTTAGCCGCGGTTGAACCTCCCAA**

**Huch11 ATTTTTTTTAAAAAACTCAAAGTATCCATTCTCCCCCCAAAAAATTTCTATGTTCCGGACCCTGTTTCTCAAAAACTAGTCATACGA-TTTTTGAATTTCATAGCTCATTCGATTCAGCTTTCAAAGCTCTTTCGAATGATATATGAATCATATAGAAATATTTTGTCGATAATAAGTTAGCCGCGGTTGAACCTCCCAA**

**HufaN4 ATTTTTTTTAAAAAACTCAAAGTATCCATTCTCCCCCCAAAAAATTTCTATGTTCCGGACCCTGTTTCTCAAAAACTAGTCATACGA-TTTTTGAATTTCATAGCTCATTCGATTCAGCTTTCAAAGCTCTTTCGAATGATATATGAATCATATAGAAATATTTTGTCGATAATAAGTTAGCCGCGGTTGAACCTCCCAA**

**HufaP1 ATTTTTTTTAAAAAACTCAAAGTATCCATTCTCCCCCCAAAAAATTTCTATGTTCCGGACCCTGTTTCTCAAAAACTAGTCATACGA-TTTTTGAATTTCATAGCTCATTCGATTCAGCTTTCAAAGCTCTTTCGAATGATATATGAATCATATAGAAATATTTTGTCGATAATAAGTTAGCCGCGGTTGAACCTCCCAA**

**HufaP4 ATTTTTTTTAAAAAACTCAAAGTATCCATTCTCCCCCCAAAAAATTTCTATGTTCCGGACCCTGTTTCTCAAAAACTAGTCATACGA-TTTTTGAATTTCATAGCTCATTCGATTCAGCTTTCAAAGCTCTTTCGAATGATATATGAATCATATAGAAATATTTTGTCGATAATAAGTTAGCCGCGGTTGAACCTCCCAA**

**HufaP8 ATTTTTTTTAAAAAACTCAAAGTATCCATTCTCCCCCCAAAAAATTTCTATGTTCCGGACCCTGTTTCTCAAAAACTAGTCATACGA-TTTTTGAATTTCATAGCTCATTCGATTCAGCTTTCAAAGCTCTTTCGAATGATATATGAATCATATAGAAATATTTTGTCGATAATAAGTTAGCCGCGGTTGAACCTCCCAA**

**HuchP9 ATTTTTTTTAAAAAACTCAAAGTATCCATTCTCCCCCCAAAAAATTTCTATGTTCCGGACCCTGTTTCTCAAAAACTAGTCATACGATTTTTTGAATTTCATAGCTCATTCGATTCAGCTTTCAAAGCTCTTTCGAATGATATATGAATCATATAGAAATATTTTGTCGATAATAAGTTAGCCGCGGTTGAACCTCCCAA**

**HufaP10 ATTTTTTTTAAAAAACTCAAAGTATCCATTCTCCCCCCAAAAAATTTCTATGTTCCGGACCCTGTTTCTCAAAAACTAGTCATACGA-TTTTTGAATTTCATAGCTCATTCGATTCAGCTTTCAAAGCTCTTTCGAATGATATATGAATCATATAGAAATATTTTGTCGATAATAAGTTAGCCGCGGTTGAACCTCCCAA**

**1810 1820 1830 1840 1850 1860 1870 1880 1890 1900 1910 1920 1930 1940 1950 1960 1970 1980 1990 2000**

2aL

**....|....|....|....|....|....|....|....|....|....|....|....|....|....|....|....|....|....|....|....|....|....|....|....|....|....|....|....|....|....|....|....|....|....|....|....|....|....|....|....|**

**H1cfa2 --------------------------------------------------------------------------------------------------------------------------------------------------------------------------------------------------------**

**H1cfa8 --------------------------------------------------------------------------------------------------------------------------------------------------------------------------------------------------------**

**H1cfa17 --------------------------------------------------------------------------------------------------------------------------------------------------------------------------------------------------------**

**H1cfa18 --------------------------------------------------------------------------------------------------------------------------------------------------------------------------------------------------------**

**H1cch2 --------------------------------------------------------------------------------------------------------------------------------------------------------------------------------------------------------**

**H1cch3 --------------------------------------------------------------------------------------------------------------------------------------------------------------------------------------------------------**

**H1cch4 --------------------------------------------------------------------------------------------------------------------------------------------------------------------------------------------------------**

**H1cch8 --------------------------------------------------------------------------------------------------------------------------------------------------------------------------------------------------------**

**H1cch9 --------------------------------------------------------------------------------------------------------------------------------------------------------------------------------------------------------**

**H1cch12 --------------------------------------------------------------------------------------------------------------------------------------------------------------------------------------------------------**

**H1cch5 --------------------------------------------------------------------------------------------------------------------------------------------------------------------------------------------------------**

**H1cch11 --------------------------------------------------------------------------------------------------------------------------------------------------------------------------------------------------------**

**Huch21 ATTATTTTTTCGGAACAAGTCTGAAATTTTCGGAACAAGTCTCATAACTTACTGAATTTTCTTCAAAAAATATTCATTCCAGCTTCAATATATTCAGAATTAAATCCTCTTTCGAATGATATATGACTCATACGAAACTTACCTACAGATTTTTTTTACAAAAATTCAAAATAGTCATTCTCCCCCAAAAAATTTCTATG**

**Huch22 ATTATTTTTTCGGAACAAGTCTGAAATTTTCGGAACAAGTCTCATAACTTACTGAATTTTCTTCAAAAAATATTCATTCCAGCTTCAATATATTCAGAANTAAATCCTCTTTCGAATGATGTATGACTCATACGAAACTTACCTACAGATTTTTTTTACAAAAATTCAAAATAGTCATTCTCCCCCAAAAAATTTCTATG**

**Huch23 ATTATTTTTTCGGAACAAGTCTGAAATTTTCGGAACAAGTCTCATAACTTACTGAATTTTCTTCAAAAAATATTCATTCCAGCTTCAATATATTCAGAATTAAATCCTCTTTCGAATGATATATGACTCATACGAAACTTACCTACAGATTTTTTTTACAAAAATTCAAAATAGTCATTCTCCCCCAAAAAATTTCTATG**

**Huch11 ATTATTTTTTCGGAACAAGTCTGAAATTTTCGGAACAAGTCTCATAACTTACTGAATTTTCTTCAAAAAATATTCATTCCAGCTTCAATATATTCAGAATTAAATCCTCTTTCGAATGATATATGACTCATACGAAACTTACCTACAGATTTTTTTTACAAAAATTCAAAATAGTCATTCTCCCCCAAAAAATTTCTATG**

**HufaN4 ATTATTTTTTCGGAACAAGTCTGAAATTTTCGGAACAAGTCTCATAACTTACTGAATTTTCTTCAAAAAATATTCATTCCAGCTTCAATATATTCAGAATTAAATCCTCTTTCGAATGATATATGACTCATACGAAACTTACCTACAGATTTTTTTTACAAAAATTCAAAATAGTCATTCTCCCCCAAAAAATTTCTATG**

**HufaP1 ATTATTTTTTCGGAACAAGTCTGAAATTTTCGGAACAAGTCTCATAACTTACTGAATTTTCTTCAAAAAATATTCATTCCAGCTTCAATATATTCAGAATTAAATCCTCTTTCGAATGATATATGACTCATACGAAACTTACCTACAGATTTTTTTTACAAAAATTCAAAATAGTCATTCTCCCCCAAAAAATTTCTATG**

**HufaP4 ATTATTTTTTCGGAACAAGTCTGAAATTTTCGGAACAAGTCTCATAACTTACTGAATTTTCTTCAAAAAATATTCATTCCAGCTTCAATATATTCAGAATTAAATCCTCTTTCGAATGATATATGACTCATACGAAACTTACCTACAGATTTTTTTTACAAAAATTCAAAATAGTCATTCTCCCCCAAAAAATTTCTATG**

**HufaP8 ATTATTTTTTCGGAACAAGTCTGAAATTTTCGGAACAAGTCTCATAACTTACTGAATTTTCTTCAAAAAATATTCATTCCAGCTTCAATATATTCAGAATTAAATCCTCTTTCGAATGATATATGACTCATACGAAACTTACCTACAGA-TTTTTTTACAAAAATTCAAAATAGTCATTCTCCCCCAAAAAATTTCTATG**

**HuchP9 ATTATTTTTTCGGAACAAGTCTGAAATTTTCGGAACAAGTCTCATAACTTACTGAATTTTCTTCAAAAAATATTCATTCCAGCTTCAATATATTCAGAATTAAATCCTCTTTCGAATGATATATGACTCATACGAAACTTACCTACAGA-TTTTTTTACAAAAATTCAAAATAGTCATTCTCCCCCAAAAAATTTCTATG**

**HufaP10 ATTATTTTTTCGGGACAAGTCTGAAATTTTCGGAACAAGTCTCATAACTTACTGAATTTTCTTCAAAAAATATTCATTCCAGCTTCAATATATTCAGAATTAAATCCTCTTTCGAATGATATATGACTCATACGAAACTTACCTACAGATTTTTTTTACAAAAATTCAAAATAGTCATTCTCCCCCAAAAAATTTCTATG**

**2010 2020 2030 2040 2050 2060 2070 2080 2090 2100 2110 2120 2130 2140 2150 2160 2170 2180 2190 2200**

**....|....|....|....|....|....|....|....|....|....|....|....|....|....|....|....|....|....|....|....|....|....|....|....|....|....|....|....|....|....|....|....|....|....|....|....|....|....|....|....|**

**H1cfa2 --------------------------------------------------------------------------------------------------------------------------------------------------------------------------------------------------------**

**H1cfa8 --------------------------------------------------------------------------------------------------------------------------------------------------------------------------------------------------------**

**H1cfa17 --------------------------------------------------------------------------------------------------------------------------------------------------------------------------------------------------------**

**H1cfa18 --------------------------------------------------------------------------------------------------------------------------------------------------------------------------------------------------------**

**H1cch2 --------------------------------------------------------------------------------------------------------------------------------------------------------------------------------------------------------**

**H1cch3 --------------------------------------------------------------------------------------------------------------------------------------------------------------------------------------------------------**

**H1cch4 --------------------------------------------------------------------------------------------------------------------------------------------------------------------------------------------------------**

**H1cch8 --------------------------------------------------------------------------------------------------------------------------------------------------------------------------------------------------------**

**H1cch9 --------------------------------------------------------------------------------------------------------------------------------------------------------------------------------------------------------**

**H1cch12 --------------------------------------------------------------------------------------------------------------------------------------------------------------------------------------------------------**

**H1cch5 --------------------------------------------------------------------------------------------------------------------------------------------------------------------------------------------------------**

**H1cch11 --------------------------------------------------------------------------------------------------------------------------------------------------------------------------------------------------------**

**Huch21 TTCAGCAACTTGCAACTTTTGACTGTTCTGTTAAAGGTTGATAATTTTTGCTTAAGAATGAAGAATATAGAATGAGAATCATAATAAAAGAAAAAAATTTGAAGAAATACCAAAGAAATAAAAAGTTAATAAATTTGTTCCCCCCATCTCCCTTTTTACACCCAGCCAAAACCCAAGCTCTCCAAAGTGGAAGTGGCTTG**

**Huch22 TTCAGCAACTTGCAACTTTTGACTGTTCTGTTAAAGGTTGATAATTTTTGCTTAAGAATGAAGAATATAGAATGAGAATCATAATAAAAGAAAAAAATTTGAAGAAATACCAAAGAAATAAAAAGTTAATAAATTTGTTCCCCCCATCTCCCTTTTTACACCCAGCCAAAACCCAAGCTCTCCAAAGTGGAAGTGGCTTG**

**Huch23 NTCAGCAACTTGCAACTTTTGACTGTTCTGTTAAAGGTTGATAATTTTTGCTTAAGAATGAAGAATATAGAATGAGAATCATAATAAAAGAAAAAAATTTGAAGAAATACCAAAGAAATAAAAAGTTAATAAATTTGTTCCCCCCATCTCCCTTTTTACACCCAGCCAAAACCCAAGCTCTCCAAAGTGGAAGTGGCTTG**

**Huch11 TTCAGCAACTTGCAACTTTTGACTGTTCTGTTAAAGGTTGATAATTTTTGCTTAAGAATGAAGAATATAGAATGAGAATCATAATAAAAGAAAAAAATTTGAAGAAATACCAAAGAAATAAAAAGTTAATAAATTTGTTCCCCCCATCTCCCTTTTTACACCCAGCCAAAACCCAAGCTCTCCAAAGTGGAAGTGGCTTG**

**HufaN4 TTCAGCAACTTGCAACTTTTGACTATTCTGTTAAAAGTTGATAATTTTTGCTTAAGAATGAAGAATATAGAATGAGAATCATAATAAAAGAAAAAAATTTGAAGAAATACCATAGAAATAAAAAGTTAATAAATTTGTTCCCCCCATCTCCCTTTTTACACCCAGCCAAAACCCAAGCTCTCCAAAGTGGAAGTGGCTTG**

**HufaP1 TTCAGCAACTTGCAACTTTTGACTGTTCTGTTAAAAGTTGATAATTTTTGCTTAAGAATGAAGAATATAGAATGAGAATCATAATAAAAGAAAAAAATTTGAAGAAATACCATAGAAATAAAAAGTTAATAAATTTGTTCCCCCCATCTCCCTTTTTACACCCAGCCAAAACCCAAGCTCTCCAAAGTGGAAGTGGCTTG**

**HufaP4 TTCAGCAACTTGCAACTTTTGACTGTTCTGTTAAAAGTTGATAATTTTTGCTTAAGAATGAAGAATATAGAATGAGAATCATAATAAAAGAAAAAAATTTGAAGAAATACCATAGAAATAAAAAGTTAATAAATTTGTTCCCCCCATCTCCCTTTTTACACCCAGCCAAAACCCAAGCTCTCCAAAGTGGAAGTGGCTTG**

**HufaP8 TTCAGCAACTTGCAACTTTTGACTGTTCTGTTAAAGGTTGATAATTTTTGCTTAAGAATGAAGAATATAGAATGAGAATCATAATAAAAGAAAAAAATTTGAAGAAATACCAAAGAAATAAAAAGTTAATAAATTTGTTCCCCCCATCTCCCTTTTTACACCCAGCCAAAACCCAAGCTCTCCAAAGTGGAAGTGGCTTG**

**HuchP9 TTCAGCAACTTGCAACTTTTGACTGTTCTGTTAAAGGTTGATAATTTTTGCTTAAGAATGAAGAATATAGAATGAGAATCATAATAAAAGAAAAAAATTTGAAGAAATACCAAAGAAATAAAAAGTTAATAAATTTGTTCCCCCCATCTCCCTTTTTACACCCAGCCAAAACCCAAGCTCTCCAAAGTGGAAGTGGCTTG**

**HufaP10 TTCAGCAACTTGCAACTTTTGACTGTTCTGTTAAAAGTTGATAATTTTTGCTTAAGAATGAAGAATATAGAATGAGAATCATAATAAAAGAAAAAAATTTGAAGAAATACCATAGAAATAAAAAGTTAATAAATTTGTTCCCCCCATCTCCCTTTTTACACCCAGCCAAAACCCAAGCTCTCCAAAGTGGAAGTGGCTTG**

**2210 2220**

**....|....|....|....|....|**

**H1cfa2 -------------------------**

**H1cfa8 -------------------------**

**H1cfa17 -------------------------**

**H1cfa18 -------------------------**

**H1cch2 -------------------------**

**H1cch3 -------------------------**

**H1cch4 -------------------------**

**H1cch6 -------------------------**

**H1cch8 -------------------------**

**H1cch9 -------------------------**

**H1cch12 -------------------------**

**H1cch11 -------------------------**

**Huch21 TCTCTTCTACACCAATCACTAGTG-**

**Huch22 TCTCTTCTACACCAATCACTAGTG-**

**Huch23 TCTCTTCTACACCAATCACTAGTG**

**Huch11 TCTCTTCTACACCAATCACTAGTG**

**HufaN4 TCTCTTCTACACC**

**HufaP1 TCTCTTCTACACC**

**HufaP4 TCTCTTCTACACC**

**HufaP8 TCTCTTCTACACC**

**HuchP9 TCTCTTCTACACC**

**HufaP10 TCTCTTCTACACC**

Figure S1 Alignment of HORs from *M. fallax* (clone names in blue) and *M. chitwoodi* (clone names in green). H1cfa(n) and H1cch(n) represent fragments amplified with 1c primers. Hufa(n) and Huch(n) are amplified with primers specific for U1 sequence. All primer positions are marked above sequences and primers are listed in Supplemental Table S1. SatDNA monomers are indicated in different colours; **1c**, **1d**, **2a**, **1a**, **1b** and **1b'**. Unlabeled part of the HOR is U1 sequence. Red boxes indicate Box 1, and black boxes represent Box 2. Sequences are deposited in EMBL databank under accession numbers: JX186856 - JX186877.
