# Supplementary material for: Conserved DNA Motifs, Including the CENP-B Box-like, Are Possible Promoters of Satellite DNA Array Rearrangements in Nematodes
Source: PLoS One. 2013 Jun 27;8(6):e67328. doi: 10.1371/journal.pone.0067328 (PMC3694981; doi:10.1371/journal.pone.0067328)
Supplement: Figure S2 — Alignment of complex fragments from M. fallax (clone names in blue) and M. chitwoodi (clone names in green). Sequences are indicated in different colours; 1a monomer (green), 1d monomer (grey) and U2 sequence (yellow). Unlabeled part belongs to U1 sequence. Blue box represents overlapping region of 1a and 1d monomers. Box 1 is indicated in red, and Box 2 in black. Grey boxes represent perfectly conserved fragment common for U1 and U2 sequences. Primer positions for U2 are indicated above sequences. Sequences are deposited in EMBL databank under accession numbers: JX186850–JX186855. (DOC) [file pone.0067328.s002.doc]

**10 20 30 40 50 60 70 80 90 100 110 120 130**

**....|....|....|....|....|....|....|....|....|....|....|....|....|....|....|....|....|....|....|....|....|....|....|....|....|....|**

**hunch1 AAGGGTGTTCCTTT-ACTCCTTCTATTTTCAAAAATTTTTTT-CTCAAAAACTAGTCGATGGATTTTTGAATTT-TATAGCTCATTCGATTCAGTAATTCGTCCTCTTACAAATGATACTAAATTCAGCA**

**hunch2 ..............-...........................-...............................-.......................................................**

**hunfa1 --T...........-...........................-...............................-.........................A.......T.....................**

**hunfa2 --T...........-...........................-...............................-.........................A.......T.....................**

**hunch3 ..............-...........................-...............................-.........................A.......T.....................**

**hunfa3 ..............-...........................-...............................-.........................A.......T.....................**

**140 150 160 170 180 190 200 210 220 230 240 250 260 270 280 290 300 310 320 330 340 350**

**....|....|....|....|....|....|....|....|....|....|....|....|....|....|....|....|....|....|....|....|....|....|....|....|....|....|....|....|....|....|....|....|....|....|....|....|....|....|....|....|....|....|....|....|**

**hunch1 AATTTCCAACGATGGAAATTTTTTTATAAAAGTTCAAAAATATTCCTTCCCCAAAATTTTTCTATACTTTCAAAAAATTTTTTCTCAAAAACTAGTCATAATATTTTTGAATTTCATAGCTCATTCGATTCAGCTTTCAAAGCTCTTTCGAATGATACCAAATTCAGTAAAATTCTATAGAGGGAAAAAAAATTTCTTCAATTTAAAAAATTTATTCACC**

**hunch2 ............................................................................................................................................................................................................................**

**hunfa1 .........................................................................................................................................................................T..................................................**

**hunfa2 .........................................................................................................................................................................T..................................................**

**hunch3 .........................................................................................................................................................................T..................................................**

**hunfa3 .........................................................................................................................................................................T..................................................**

U2L

U2L

**360 370 380 390 400 410 420 430 440 450 460 470 480 490 500 510 520 530 540 550 560 570**

**....|....|....|....|....|....|....|....|....|....|....|....|....|....|....|....|....|....|....|....|....|....|....|....|....|....|....|....|....|....|....|....|....|....|....|....|....|....|....|....|....|....|....|....|**

**hunch1 CCAAAAAATTTCTATGTTCAGCAACTTGCAACTTTTGACTGTCTTGTTAGATATTTACAATTTTGGTACCTACATCATCTATATAGAATGGGAATCATAATAAAACAAAAAAATTTGACGAAGTACCATAGAAATAAAAAGTTAGAAAAATTTGATTTTTTCAAATTTAAAGTACAAATTCTTTTCAAAATTTTAAAACTTTTGACTGTTCTGTTAAAAG**

**hunch2 ............................................................................................................................................................................................................................**

**hunfa1 .........................................................................................................................C..........................................................T.......................................**

**hunfa2 .................................................................................................... ....................C..........................................................T.......................................**

**hunch3 ....................-...............................................................................................................................................................T.......................................**

**hunfa3 ....................................................................................................................................................................................T........... ............................**

**580 590 600 610 620 630 640 650 660 670 680 690 700 710 720 730 740**

**....|....|....|....|....|....|....|....|....|....|....|....|....|....|....|....|....|....|....|....|....|....|....|....|....|....|....|....|....|....|....|....|....|....|....|...**

**hunch1 TTAATAATTTTTGTTTTAGGATGAAGAATATAGAATGAGAATCATAATAAAACAAAAAAATTTGACGAAAGACCATAAGAATAAAAAGTTATAAGATTAGTCCCCCCCATTTCCCCTTCAACAACCAATCAAAGCCCAAAGCCCTACAAAGTGGCCGTGGCTTGTCTCTTCTACACCA**

**hunch2 ..................................................................................................................................................................................**

**hunfa1 ...................A..............................................................................................................................................................**

**hunfa2 ...................A..............................................................................................................................................................**

**hunch3 ...................A.......................................................................................................................-...........................A.........T**

**hunfa3 ...................A...................................................................................................................................................A.........T**

Figure S2. Alignment of complex fragments from *M. fallax* (clone names in blue) and *M. chitwoodi* (clone names in green) amplified with primers specific for U1 sequence. Sequences are indicated in different colours; 1a monomer (green), 1d monomer (grey) and U2 sequence (yellow). Unlabeled part belongs to U1 sequence. Blue box represents overlapping region of 1a and 1d monomers. Box 1 is indicated in red, and Box 2 in black. Grey boxes represent perfectly conserved fragment common for U1 and U2 sequences. Primer positions for U2 are indicated above sequences. Sequences are deposited in EMBL databank under accession numbers: JX186850 - JX186855.
