# Supplementary material for: Conserved DNA Motifs, Including the CENP-B Box-like, Are Possible Promoters of Satellite DNA Array Rearrangements in Nematodes
Source: PLoS One. 2013 Jun 27;8(6):e67328. doi: 10.1371/journal.pone.0067328 (PMC3694981; doi:10.1371/journal.pone.0067328)
Supplement: Figure S3 — Alignment of 1a, 1b, 1b’, 1c, 1d, 2a and 2b monomers from M. fallax and M. chitwoodi . Monomers are extracted from monomeric and HOR arrays using KSA algorithm [26]. All monomers are compared with first sequence and positions identical to the first sequence are shown with dot. Monomer group are indicated on the right side. Monomer sequences are deposited in EMBL data bank under accession numbers: JX186757–JX186849 and JX186878–JX186996. Box 1 is shaded with yellow. Detail description of satellite monomers are indicated below alignment. (DOC) [file pone.0067328.s003.doc]

10 20 30 40 50 60 70 80 90 100 110 120 130 140 150 160 170 180 190 200

**Box 1**

....|....|....|....|....|....|....|....|....|....|....|....|....|....|....|....|....|....|....|....|....|....|....|....|....|....|....|....|....|....|....|....|....|....|....|....| ....|....|....|....|

**1dHch2**  **----CAGTGTTCATGAACCTGTTT-TCTCAAAAACTAGTCA--TACGATTTTTGAT-TTTA-TAGCTCATTCGATACAGCTCTTTAAGCTCTTTCGAATGATACT-AAATTCAGT-ATACTTTCTCAGAGGGAAAAAAAATTTC-------TTCAATTTAAAA-AATT--TTTTCCCCCAAAAAAATTT**

**1dHch3**  **----..A.................-................--.............-...CA...........................................-.........-............................-------............-....--...................**

**1dHch4**  **----..A.................-................--.............-...CA...........................................-.........-............................-------............-....--...................**

**1dHch6**  **----..A.................-................--.............-...CA...........................................-.........-.....C......................-------............-....--...................**

**1dHch8**  **----..A.................-................--.............-.C.-A...........................................-.........-............................-------............-....--...................**

**1dHch9**  **----....................-................--.............-...CA...........................................-.........-.......................-....-------............-....--...................**

**1dHch12**  **----..A.................-................--.............-...CA...........................................-.........-............................-------............-....--...................**

**1dHch21**  **----..A.................-................--.............-...CA...........................................-.........-............................-------............-....--...................**

**1dHch22**  **----....................-................--.............-...CA...........................................-.........-............................-------............-....--...................**

**1dHch23**  **----..A.................-................--.............-...CA...........................................-.........-............................-------............-....--...................**

**1dHch11**  **----..A.................-................--.............-...CA...........................................-.........-............................-------............-....--...................**

**1dHfaN4**  **----....................-................--.............-...CA...........................................-.........-............................-------............-....--...................**

**1dHfaP1**  **----..A.................-................--.............-...CA...........................................-.........-............................-------............-....--...................**

**1dHfaP4**  **----....................-................--.............-...CA...........................................-.........-............................-------............-....--...................**

**1dHD**

**1dHfaP8**  **----..A.................-........T.......--.TAA......TT.-AAA.A...................T..C.T..................-.........-............................-------............-....--...................**

**1dHchP9**  **----..A.................-........T.......--.TAA......TT.-AAA.A...................T..C.T..................-.........-............................-------............-....--...................**

**1dHfaP10**  **----....................-................--.............-...CA...........................................-.........-............................-------............-....--...................**

**1dHch13a**  **----..A.................-................--............A-...CA....A......................................-.........-............................-------............-....--...................**

**1dMch13b**  **----..A.................-................--............A-...CA....A......................................-.........-............................-------............-....--...................**

**1dMch13c**  **----..A.................-................--.........-..A-...CA....A......................................-.........-............................-------............-....--...................**

**1dMfa16a**  **----..A.................-................--............A-...CA....A......................................-.........-...............-............-------............-....--................A..**

**1dMfa16b**  **----..A.................-................--............A-...CA....A......................................-.........-............................-------............-....--...................**

**1dMfa11a**  **----..A.................-................--.........-..A-...CA....A......................................-.........-............................-------............-....--...................**

**1dMfa11b**  **----..A.................-................--............A-...CA....A.................................G....-.........-............................-------............-....--...................**

**1dMfa11c**  **----....................-................--............A-...CA...........................................-.........-............................-------............-....--...................**

**1dMfa2a**  **----..A.................-................--............A-...CA....A......................................-.........-......................G.....-------............-....--...................**

**1dMfa2b**  **----..A.................-................--............A-..-CA....A......................................-.........-............................-------............-....--...-...............**

**1dMfa2c**  **----....................-................--.........-..A-...CA....A......................................-.........-............................-------............-....--...................**

**1dMch3a**  **----..A.................-................--............A-...CA....A......................................-.........-............................-------............-....--...................**

**1dMch3b**  **----..A.................-................--............A-...CA....A......................................-.........-............................-------............-....--...................**

**1dMch3c**  **----..A.................-................--............A-...CA....A......................................-.........-............................-------............-....--...................**

**1cHch2**  **----.TA.....CG..C......--....G.GTT..GTCTG--..A.T.GC.....-..C.A.....TG......T..C.....C.TC.............G.TA-TG.C...TCC...AC..AC.TACA.--..TTTTT...A-------AAA..C.C...G-T..CCA..C......C.........**

**1cHch3**  **----.TA.....CG..C......--....G.GTT..GTCTG--..A.T.GC.....-..C.A.....TG......T..C.....C.TC.............G.GA-TG.C...TCC...AC..ACTTACA.--..TTTTT...A-------AAA..C.C...G-T..CCA..C......C.........**

**1cHch4**  **----.TA.....CG..C......--....G.GTT..GTCTG--..A.T.GC.....-..C.A.....TG......T..C.....C.TC.............G.TA-TG.C...TCC...AC..ACTTACA.--..TTTTT...A-------AAA..C.C...G-T..CCA..C......C.........**

**1cHch6**  **----.TA.....CG..C......--....G.GTT..GTCTG--..A.T.GC.....-..C.A.....TGC.....T..C.....C.TC.............G.TA-TG.C...TCC...AC..AC.TACA.--..TTTTT...A-------AAA..C.C...G-T..CCA..C......C.........**

**1cHch8**  **----.TA.....CG..C......--....G.GTT..GTCTG--..A.T.GC.....-..C.A.....TG......T..C.....C.TC.............G.TA-TG.C...TCC...AC..ACTTACA.--..TTTTT...A-------AAA..C.C...G-T..CCA..C......C.........**

**1cHch9**  **----.TA.....CG..C......--....G.GTT..GTCTG--..A.T.GC.....-..C.A.....TG......T..C.....C.TC.............G.TA-TG.C...TCC...AC..AC.TACA.--..TTTTT...A-------AAA..C.C...G-T..CCA..C......C.........**

**1cHch12**  **----.TA.....CG..C......--....G.GTT..GTCTG--..A.T.GC.....-..C.A.....TG......T..C.....C.TC.............G.TA-TG.C...TCC...AC..ACTTACA.--..TTTTT...A-------AAA..C.C...G-T..CCA..C......C.........**

**1cHch21**  **----.TA.....CG..C......--....G.GTT..GTCTG--..A.T.GC.....-..C.A.....TG......T..C.....C.TC.............G.TA-TG.C...TCC...AC..ACTTACA.--..TTTTT...A-------AAA..C.C...G-T..CCA..C......C.........**

**1cHch22**  **----.TA.....CG..C......--....G.GTT..GTCTG--..A.T.GC.....-..C.A.....TG......T..C.....C.TC.............G.TA-TG.C...TCC...AC..AC.TACA.--..TTTTT...A-------AAA..C.C...G-T..CCA..C......C.........**

**1cHch23**  **----.TA.....CG..C......--....G.GTT..GTCTG--..A.T.GC.....-..C.A.....TG......T..C.....C.TC.............G.TA-TG.C...TCC...AC..AC.TACA.--..TTTTT...A-------AAA..C.C...G-T..CCA..C......C.........**

**1cHch11**  **----.TA.....CG..C......--....G.GTT..GTCTG--..A.T.GC.....-..C.A.....TG......T..C.....C.TC.............G.TA-TG.C...TCC...AC..ACTTACA.--..TTTTT...A-------AAA..C.C...G-T..CCA..C......C.........**

**1cHfaN4**  **----.TA.....CG..C......--....G.GTT..GTCTG--..A.T.GC.....-..C.A.....TG......T..C.....C.TC.............G.TA-TG.C...TCC...AC..AC.TACA.--..TTTTT...A-------AAA..C.C...G-T..CCA..C......C.........**

**1cHD**

**1cHfaP1**  **----.TA.....CG..C......--....G.GTT..GTCTG--..A.T.GC.....-..C.A.....TG......T..C.....C.TC.......T.....G.TA-TG.C...TCC...AC..AC.TACA.--..TTTTT...A-------AAA..C.C...G-T..CCA..C......C.........**

**1cHfaP4**  **----.TA.....CG..C......--....G.GTT..GTCTG--..A.T.GC.....-..C.A.....TG......T..C.....C.TC.......T.....G.TA-TG.C...TCC...AC..AC.TACA.--..TTTTT...A-------AAA..C.C...G-T..CCA..C......C.........**

**1cHfaP8**  **----.TA.....CG..C......--....G.GTT..GTCTG--..A.T.GC.....-..C.A.....TG......T..C.....C.TC.............G.TA-TG.C...TCC...AC..AC.TACA.--..TTTTT...A-------AAA..C.C...G-T..CCA..C......C.........**

**1cHchP9**  **----.TA.....CG..C......--....G.GTT..GTCTG--..A.T.GC.....-..C.A.....TG......T..C.....C.TC.............G.TA-TG.C...TCC...AC..AC.TACA.--..TTTTT...A-------AAA..C.C...G-T..CCA..C......C.........**

**1cHfaP10**  **----.TA.....CG..C......--....G.GTT..GTCTG--..A.T.GC...G.-..C.A.....TG......T..C.....C.TC.............G.TA-TG.C...TCC...AC..AC.TACA.--..TTTTT...A-------AAA..C.C...G-T..CCA..C......C.........**

**1cMch13a**  **----.TA.....CG..C......--....G.GTT..GTCTG--..A.T.GC.....-..C.A.....TG......T..C.....C.TC.............G.TA-TG.C...TCC...AC..AC.TACA.--..TTTTT...A-------AAA..C.C...G-T..CCA..C......C.........**

**1cMch13b**  **----.TA.....CG..C......--....G.GTT..GTCTG--..A.T.GC.....-..C.A.....TGC.....T..C.....C.TC.......T.....G.TA-TG.C...TCC...AC..AC.TACA.--..TTTTT...A-------AAA..C.C...G-T..CCA..C......C.........**

**1cMch13c**  **----.TA.....CG..C......--....G.GTT..GTCTG--..A.T.GC.....-..C.A.....TG......T..C.....C.TC.............G.TA-TG.C...TCA...AC..AC.TACA.--..TTTTT...A-------AAA..C.C...G-T..CCA..C......C.........**

**1cMfa16a**  **----.TA.....CG..C......--....G.GTT..GTCTG--..A.T.GC.....-..C.A.....TG......T..C.....C.TC.............G.TA-TG.C...TCC...AC..AC.TACA.--..TTTTT...A-------AAA..C.C...G-T..CCA..C......C.........**

**1cMfa16b**  **----.TA.....CG..C......--....G.GTT..GTCTG--..A.T.GC.....-..C.A.....TG......T..C.....C.TC.......T.....G.TA-TG.C...TCC...AC..AC.TACA.--..TTTTT...A-------AAA..C.C...G-T..CCA..C......C.........**

**1cMfa11a**  **----.TA.....CG..C......--....G.GTT..GTCTG--..A.T.GC.....-..C.A.....TGC.....T..C.....C.TC.............G.TA-TG.C...TCA...AC..AC.TACA.--..TTTTT...A-------AAA..C.C...G-T..CCA..C......C.........**

**1cMfa11b**  **----.TA.....CG..C......--....G.GTT..GTCTG--..A.T.GC.....-..C.A.....TG......T..C.....C.TC.............G.TA-TG.C...TCC...AC..AC.TACA.--..TTTTT...A-------AAA..C.C...G-T..CCA..C......C.........**

**1cMfa11c**  **----.TA.....CG..C......--....G.GTT..GTCTG--..A.T.GC.....-..C.A.....TG......T..C.....C.TC.............G.TA-TG.C...TCC...AC..AC.TACA.--..TTTTT...A-------AAA..C.C...G-T..CCA..C......C.........**

**1cMfa2a**  **----.TA.....CG..C.....---....G.GTT..GTCTG--..A.T.GC.....-..C.A.....TGC.....T..C.....C.TC.............G.TA-TG.C...TCC...AC..AC.TACA.--..TTTTT...A-------AAA..C.C...G-T..CCA..C......C.........**

**1cMfa2b**  **----.TA.....CG..C.....---....G.GTT..GTCTG--..A.T.GC.....-..C.A.....TG......T..C.....C.TC.............G.TA-TG.C...TCC...AC..AC.TACA.--..TTTTT...A-------AAA..C.C...G-T..CCA..C......C.........**

**1cMfa2c**  **----.TA.....CG..C.....---....G.GTT..GTCTG--..A.T.GC.....-..C.A.....TGC.....T..C.....C.TC.............G.TA-TG.C...TCC...AC..AC.TACA.--..TTTTT...A-------AAA..C.C...G-T..CCA..C......C.........**

**1cMch3a**  **----.TA.....CG..C.....---....G.GTT..GTCTG--..A.T.GC.....-..C.A.....TG......T..C.....C.TC.............G.TA-TG.C...TCC...AC..AC.TACA.--..TTTTT...A-------AAA..C.C...G-T..CCA..C......C.........**

**1cMch3b**  **----.TA.....CG..C.....---....G.GTT..GTCTG--..A.T.GC.....-..C.A.....TG......T..C.....C.TC.............G.TA-TG.C...TCC...AC..AC.TACA.--..TTTTT...A-------AAA..C.C...G-T..CCA..C......C.........**

**1cMch3c**  **----.TA.....CG..C.....---....G.GTT..GTCTG--..A.T.GC.....-..C.A.....TG......T..C.....C.TC.............G.TA-TG.CA..TCC...AC..AC.TACA.--..CTTTT...A-------AAA..C.C...G-T..CCA..C......C.........**

**1aHfa2___**  **-----__.T.CA.AA.TTT.T..---..............G-A.GGAT....GA.--...CA.............T..........C........A.........-........A--A.AA-.TC..AC.ATGG...TTT...T----AT-A.A.G..C....AT...CC..C---...C...TTT..C**

**1aHfa8__**  **-------.T....AA..TT.T...--..............G-A.GGAT....GA.--...CA.............T..........C........A.........-........A--A.AA-.TC..AC.ATGG...TTT...T----AT-A.A.G..C....AT...CC..C---...C...TTT..C**

**1aHfa17___** **-------.T....AA..TT.T...--..............G-A.GGAT....GA.--...CA.............T..........C........A.........-........A--A.AA-.TC..AC.ATGG...TTT...T----AT-A.A.G..C....AT...CC..C---...C...TTT..C**

**1aHch21**  **-------.T....AA..TT.T...--..............G-A.GGAT....GA..-..-TA.............T...TAA..C.TC.......A.........-........C--A.A.-.TC..AC.ATGG...TTT...T----AT-AAA.T..C...TAT...CC..C---...C...TTT..C**

**1aHch22**  **-------.T....AA..TT.T...--..............G-A.GGAT....GA..-..-TA.............T...TAA..C.TC.......A.........-........C--A.A.-.TC..AC.ATGG...TTT...T----AT-AAA.T..C...TAT...CC..C---...C..-TTT..C**

**1aHch23**  **-------.T....AA..AT.T...--..............G-A.GGAT....GA..-..-CA.............T..........C........A.........-........A--A.AA-.TC..AC.ATGG...TTT...T----AT-AAA.G..C....AT...CC.-C---...C...TTT..C**

**1aHch11a**  **-------.T....AA..TT.T...--..............G-A.GGAT....GA..-..-TA.............T...TAA..C.TC.......A.........-........C--A.A.-.TC..AC.ATGG...TTT...T----AT-AAA.T..C...TAT...CC..C---...C...TTT..C**

**1aHfaN4**  **-------.T....AA..TT.T...--..............G-A.GGAT....GA..-..-CA.............T..........TC.......A.........-........A--A.AA-.TC..AC.ATGG...TTT...T----AT-A.A.G..C....AT...CC..C---...C...TTT..C**

**1aHfaP1**  **-------.T....AA..TT.T...--..............G-A.GGAT....GA..-..-CA.............T..........C........A.........-........A--A.AA-.TC..AC.ATGG...TTT...T----AT-A.A.G..C....AT...CC..C---...C...TTT..C**

**1aHfaP4**  **-------.T....AA..TT.T...--..............G-A.GGAT....GA..-..-CA.............T..........C........A.........-........A--A.AA-.TC..AC.ATGG...TTT...T----AT-A.A.G..C....AT...CC..C---...C...TTT..C**

**1aH**

**1aHfaP8**  **-------.T....AA..AA.T...--..............G-A.GGAT....GA..-..-TA.............T...................A.........-........C--A.A.-.TC..AC.ATGG...TTT...T----AT-AAA.G..C....AT...CC..C---.......TTT..C**

**1aHchP9**  **-------.T....AA..AA.T...--..............G-A.GGAT....GA..-..-TA.............T...................A.........-........C--A.A.-.TC..AC.ATGG...TTT...T----AT-AAA.G..C....AT...CC..C---.......TTT..C**

**1aHfaP10**  **-------.T....AA..TT.T...--..............G-A.GGAT....GA..-..-CA.............T..........C........A.........-........A--A.AA-.TC..AC.ATGG...TTT...T----AT-A.A.G..C....AT...CC..C---...C...TTT..C**

**1aHch5__**  **-------.T....AA..TT.T...--..............G-A.GGAT....GA.--...CA.............T..........C........A.........-........A--A.AA-.TC..AC.ATGG..CTTT...T----AT-AAA.T..C...TAT...CC..C---...C...TTT..C**

**1aHch3__**  **-------.T....AA..TT.T..---..............G-A.GGAT....GA.--...TA...........................................-........C--A.A.-.TC..AC.ATGG.-TTTT...T----AT-AAA.G..C....AT...CC.CC---......-TTT..C**

**1aHch8__**  **-------.T....AA..TT.T...--..............G-A.GGAT....GA.--...CA.............T...................A......TT.-........C--A.A.-.TC..AC.ATGG..TTTT...T----AT-AAA.G..C....AT...CC.CC---......-TTT..C**

**1aHfa18_**  **-------.T....AA..AT.T..---..............G-A.GGAT....GA.--...TA.............T...................A.........-........C--A.A.-.TC..AC.ATGG...TTT...T----AT-AAA.G..C....AT...CC..C---.......TTT..C**

**1aHch21a_**  **-------.T....AA..TT.T...--..............G-A.GGAT....GA.--...TA.............T...TAA..C.TC.......A.........-........C--A.A.-.TC..AC.ATGG...TTT...T----AT-AAA.T..C...TAT...CC..C---...C...TTT..C**

**1aHch22a_**  **-------.T....AA..TT.T...--..............G-A.GGAT....GA.--...TA.............T...TAA..C.TC.......A.........-........C--A.A.-.TC..AC.ATGG...TTT...T----AT-AAA.T..C...TAT...CC..C---...C..-TTT..C**

**1aHch4_**  **-------.T....AA..TT.T...--..............G-A.GGAT....GA.--...TA.............T...TAA..C.TC.......A.........-........C--A.A.-.TC..AC.ATGG...TTT...T----AT-AAA.T..C...TAT...CC..C---...C...TTT..C**

**1aHch12__**  **-------.T....AA..TT.T...--..............G-A.GGAT....GA.--...TA.............T...TAA..C.TC.......A.........-........C--A.-.-.TC..AC.ATGG...TTT...T----AT-AAA.T..C...TAT...CC..C---...C...TTT..C**

**1aHch6__**  **-------.T....AA..TT.T...--..............G-A.GGAT....GA.--...TA.............T...TAA..C.TC.......A.........-........C--A.A.-.TC..AC.ATGG...TTT...T----AT-AAA.T..C...TAT...CC..C---...C...TTT..C**

**1aHch9__**  **-------.T....AA..TT.T...--..............G-A.GGAT....GA.--...TA.............T...TAA..C.TC.......A.........-........C--A.A.-.TC..AC.ATGG...TTT...T----AT-AAA.G..C....AT...CC.-C---...C...TTT..C**

**1aHch11__**  **-------.T....AA..AT.T...--..............G-A.GGAT.....TT--AAATAA............T...TAA..C.TC.......A.........-........C--A.A.-.TC..AC.ATGG..CTTT...T----AT-AAA.G..C....AT...CC..C---...C...TTT..C**

**1aHch2__**  **-------.T....AA..AT.T...T-..............T-G..GAT.....TT.-AAATAA............T...TAA..C.TC.......A.........-........C--A.A.-.TC..AC.ATGG..CTTT...T----AT-AAA.G..C....AT...CC.-C---...C...TTT..C**

**1aMfa6d**  **----------------.TT.T...--..............G-A..GAT....AA.--...CAA............T.....TA..G..................A-........C--A.A.-.TC..AC.TTGG...TTT...T----AT-CGA.C...G..T-T...CC..CA--A..CC...TT...A**

**1aMfa1d**  **--CAA.A.T....AA..TT.T...--..............G-A..GAT....AA.--...CAA............T.....TA..G..................A-........C--A.A.-.TC..AC.TTGG...TTT...T----AT-CGA.C...G..T-T...CC..CA--A..CC...TT...A**

**1aMfa2d**  **--CAA.A.T....AA..TT.T...--..............G-A..GAT....AA.--...CAA............T.....TA..G..................A-........C--A.A.-.TC..AC.TTGG...TTT...T----AT-CGA.C...G..T-T...CC..CA--A..CC...TT...A**

**1aMfa3d**  **--CAA.A.T....AA..TT.T...--..............G-A..GAT....AA.--...CAA............T.....TA..G..................A-........C--A.A.-.TC..AC.TTGG...TTT...T----AT-CGA.C...G..T-T...CC..CA--A..CC...TT...A**

**1aMfa7d**  **--CAA.A.T....AA..TT.T...--..............G-A..GAT....AA.--...CAA............T.....TA..G..................A-........C--A.A.-.TC..AC.TTGG...TTT...T----AT-CGA.C...G..T-T...CC..CA--A..CC...TT...A**

**1aMfa4d**  **--CAA.A.T....AA..TT.T...--..............G-A..GAT....AA.--...CAA............T.....TA..G..................A-........C--A.A.-.TC..AC.TTGG...TTT...T----AT-CGA.C...G..T-T...CC..CA--A..CC...TT...A**

**1aMfa2c**  **--CAA.A.T....AA..TT.T...--..............G-A..GAT....AA.--...CAA............TT....TA..G..................C-........C--A.A.-.TC..AC.AT.G...TTT...T----AT-CGA.C...G..T-T...CC..CA--A..CC...TT...A**

**1aMfa4c**  **--CAA.A.T....AA..TT.T...--..............G-A..GAT....AA.--...CAA............TT....TA..G..................C-........C--A.A.-.TC..AC.AT.G...TTT...T----AT-CGA.C...G..T-T...CC..CA--A..CC...TT...A**

**1aMfa6c**  **--CAA.A.T....AA..TT.T...--..............G-A..GAT....AA.--...CAA............TT....TA..G..................C-........C--A.A.-.TC..AC.AT.G...TTT...T----AT-CGA.C...G..T-T...CC..CA--A..CC...TT...A**

**1aMfa3c**  **--CAA.A.T....AA..TT.T...--..............G-A..GAT....AA.--...CAA............TT....TA..G..................C-........C--A.A.-.TC..AC.AT.G...TTT...T----AT-CGA.C...G..T-T...CC..CA--A..CC...TT...A**

**1aMfa7c**  **--CAA.A.T.-..AA..TT.T...--..............G-A..GAT....AA.--...CAA............TT....TA..G..................C-........C--A.A.-.TC..AC.AT.G...TTT...T----AT-CGA.C...G..T-T...CC..CA--A..CC...TT...A**

**1aMfa1c**  **--CAA.A.T....AA..TT.T...--..............G-A..GAT....AA.--...CAA............TT....TA..G..................C-........C--A.A.-.TC..AC.AT.G...TTT...T----AT-CGA.C...G..T-T...CC..CA--A..CC...TT...A**

**1aMfa6a**  **----A.A.T....AA..TT.T..---..............G-A..GAT....AA.--...CA.............T.....TA.CG..................C-........C--A.A.-.TC..AC.AT.G...TTT...T----AT-CGA.C...G..T-T...CC..CA--T..CC...TT...A**

**1aMfa7a**  **----A.A.T....AA..TT.T..---..............G-A..GAT....AA.--...CA.............T.....TA.CG..................C-........C--A.A.-.TC..AC.AT.G...TTT...T----AT-CGA.C...G..T-T...CC..CA--T..CC...TT...A**

**1aMfa2a**  **----A.A.T....AA..TT.T...--..............G-A..GAT....AA.--...CA.............T.....TA.CG..................C-........C--A.A.-.TC..AC.AT.G...TTT...T----AT-CGA.C...G..T-T...CC..CA--T..CC...TT...A**

**1aMfa3a**  **----A.A.T....AA..TT.T...--..............G-A..GAT....AA.--...CA.............T.....TA.CG..................C-........C--A.A.-.TC..AC.AT.G...TTT...T----AT-CGA.C...G..T-T...CC..CA--T..CC...TT...A**

**1aMfa4a**  **----A.A.T....AA..TT.T...--..............G-A..GAT....AA.--...CA.............T.....TA.CG..................C-........C--A.A.-.TC..AC.AT.G...TTT...T----AT-CGA.C...G..T-T...CC..CA--T..CC...TT...A**

**1aMch13b**  **----A.A.T....AA..AT.T...--..............G-A..GAT....AA.--...CA.............T.....TA.CG..................C-........C--A.A.-.TC..AC.TTGG...TTT...T----AT-CGAGC...G..T-T...CC..CA--A..CC...TT...A**

**1aMch3b**  **--CAA.A.T....AA..AT.T...--..............G-A..GAT....AA.--...CA.............T.....TA.CG..................C-........C--A.A.-.TC..AC.TTGG...TTT...T----AT-CGAGC...G..T-T...CC..CA--A..CC...TT...A**

**1aMch5b**  **----A.A.T....A...AT.T...--..............G-A..GAT....AA.--...CA.............T.....TA.CG..................C-........C--A.A.-.TC..AC.TTGG...TTT...T----AT-CGA.C...G..T-T...CC..CC---..CT...TT...A**

**1aMch4a**  **----A.A.T....AA..AT.T...T-..............G-A..GAT....AA.--...CA........GT...T....ATA..G..................C-........C--A.A.-.TC..AC.ATG....TTT...T----AT-CGA.C...G..T-T...CCG.CA--A..CC...TT...A**

**1aMch5a**  **----A.A.T....AA..AT.T...T-..............G-A..GAT....AA.--...CA........GT...T....ATA..G..................C-........C--A.A.-.TC..AC.ATG....TTT...T----AT-CGA.C...G..T-T...CC..CA--A...C...TT...A**

**1aM**

**1aMfa3b**  **----A.A-T....AA..TT.T...--.......T......T-A..GAT....AA.--..ACA.............T.....TA..G..................C-........C--A.A.-.TC..AC.ATGG...TTT...T----AT-CGA.C...G..T-T...CCG.CA--A..CC...TT...A**

**1aMfa6b**  **----A.A.T....AA..TT.T...--.......T......T-A..GAT....AA.--..ACA.............T.....TA..G..................C-........C--A.A.-.TC..AC.ATGG...TTT...T----AT-CGA.C...G..T-T...CCG.CA--A..CC...TT...A**

**1aMfa7b**  **----A.A.T....AA..TT.T...--.......T......T-A..GAT....AA.--..ACA.............T.....TA..G..................C-........C--A.A.-.TC..AC.ATGG...TTT...T----AT-CGA.C...G..T-T...CCG.CA--A..CC...TT...A**

**1aMfa1b**  **----A.A.T....AA..TT.T...--.......T......T-A..GAT....AA.--..ACA.............T.....TA..G..................C-........C--A.A.-.TC..AC.ATGG...TTT...T----AT-CGA.C...G..T-T...CCG.CA--A..CC...TT...A**

**1aMfa2b**  **----A.A.T....AA..TT.T...--.......T......T-A..GAT....AA.--..ACA.............T.....TA..G..................C-........C--A.A.-.TC..AC.ATGG...TTT...T----AT-CGA.C...G..T-T...CCG.CA--A..CC...TT...A**

**1aMfa4b**  **----A.A.T....AA..TT.T...--.......T......T-A..GAT....AA.--..ACA.............T.....TA..G..................C-........C--A.A.-.TC..AC.ATGG...TTT...T----AT-CGA.C...G..T-T...CCG.CA--A..CC...TT...A**

**1aMfa1e**  **----A.A.T....AA..TT.T...--.......T......T-A..GAT....AA.--..ACA.............T.....TA..G..................C-........C--A.A.-.TC..AC.AT.G...TTT...T----AT-CGA.C...G..T-T...CCG.CA--A..CC...TCGA--**

**1aMch8b**  **----A.A.T....AA..TT.T...--...........T..T-A..GAT....AA.--...CA.....T.......T.....TA.CG..................C-........C--A.A.-.TC..AC.ATGG...TTT...T----GT-CGA.C...G..T-T...CCG.CA--A..CC...TT...A**

**1aMch8c**  **----A.A.T....AA..TT.T...--...........T..T-A..GAT....AA.--...CA.....T.......T.....TA.CG..................C-........C--A.A.-.TC..AC.ATGG...TTT...T----AT-GGA.C...G..T-T...CCG.CA--A..CC...TT...A**

**1aMch8d**  **----A.A.T....AA..TT.T...--..............T-A..GAT....AA.--...CA.....T.......T.....TA.CG..................C-........C--A.A.-.TC..AC.ATGG...TTT...T----AT-GGA.C...G..T-T...CC..CA--A..CC...TT...A**

**1aMch13d**  **----A.A.T....AA..TT.T...--.A............T-A..GAT....AA.--...CA.....T.......T.....TA.CG.....A............C-.......T.--A.A.-.TC..AC.ATGG...TTT...T----AT-CGA.C...G..T-T...CCG.CA--A..CC...TT...A**

**1aMch4b**  **----A.A.T....AA..TT.T...--.A............T-A..GAT....AA.--...CA.....T.......T.....TA.CG.....A............C-.......T.--A.A.-.TC..AC.ATGG...TTT...T----AT-CGA.C...G..T-T...CCG.CA--A..CC...TT...A**

**1aMch3d**  **----A.A.T....AA..TT.T...--.A............T-A..GAT....AA.--...CA.....T.......T.....TA.CG.....A............C-.......T.--A.A.-.TC..AC.ATGG...TTT...T----AT-CGA.C...G..T-T...CCG.CA--A..CC...TT...A**

**1aMch13c**  **--CAA.A.T....AA..AA.T...--..............T-A..GAT....AA.--...CA.....T.......T.....TA.CG.....A............C-.......T.--A.A.-CTC..AC.ATGG...TTT...T----AT-CGA.C...G..T-T...CCG.CA--A..CC...TT...A**

**1aMch3c**  **----A.A.T....AA..AA.T...--..............T-A..GAT....AA.--...CA.....T.......T.....TA.CG.....A............C-.......T.--A.A.-CTC..AC.ATGG...TTT...T----AT-CGA.C...G..T-T...CCG.CA--A..CC...TT...A**

**1aMch10b**  **----A.A.T....AA..TT.T...--..............T-A..GAT....AA.--...CA.....T.......T.....TA.CG.....A............C-.......T.--A.A.-.TC..AC.ATGG...TTT...-----AT-CGA.C...G..T-T...CC..CA--A..CC...TT...A**

**1aMch10c**  **----A.A.T....AA..TT.T...--.A............T-A..GAT....AA.--...CA.....T.......T.....TA.CG.....A............C-........C--A.A.-.TC..AC.ATGG...TTT...T----AT-CGA.C...G..T-T...CCG.CA--A..CC...TT...A**

**1aMch9c**  **----A.A.T....AA..TT.T...--.A............T-A..GAT....AA.--...CA.....T.......T.....TA.CG.....A............C-........C--A.A.-.TC..AC.ATGG...TTT...T----AT-CGA.C...G..T-T...CCG.CA--A..CC...TT...A**

**1aMch9d**  **----A.A.T....AA..TT.T...--.A............T-A..GAT....AA.--...CA.....T.......T.....TA.CG.....A............C-........C--A.A.-.TC..AC.AT.G...TTT...T----AT-CGA.C...G..T-T...CCG.CA--A..CC...TT...A**

**1aMch10d**  **----A.A.T....AA..TT.T...--.A............T-A..GAT....AA.--...CA.....T.......T.....TA.CG.....A............C-........C--A.A.-.TC..AC.AT.G...TTT...T----AT-CGA.C...G..T-T...CC..CA--A..CT...TT...A**

**1aMch10a**  **----A.A.T....AA.TTT.T...--..............G-A..GAT....AA.--..ACA.............T.....TA.CG..................C-........C--A.A.-.TC..AC.ATGG...TTT...T----AT-GCA.C...G..T-T...CCG.CA--A..CC...TT...A**

**1aMch9a**  **----A.A.T....AA.TTT.T...--..............G-A..GAT....AA.--..ACA.............T.....TA.CG..................C-........C--A.A.-.TC..AC.ATGG...TTT...T----AT-GCA.C...G..T-T...CCG.CA--A..CC...TT...A**

**1aMch6b**  **----A.A.T....AA.TTT.T...T-..............G-A..GAT....AA.--...CA.............T.....TA.CG.................TC-........C--A.A.-.TC..AC.ATGG...TTT...T----AT-GCA.C...G..T-T...CCG.CA--A..CC...TT...A**

**1aMch6a**  **----A.A.T....AA.TTT.T...T-..............G-A..GAT....AA.--...CA-............T.....TA.CG..................C-........C--A.A.-.TC..AC.ATGG...TTT...T----AT-GCA.C...G..T-T...CC..CA--A..CC...TT...A**

**1aMch9b**  **----A.A.T....AA..TT.T...--..............G-A..GAT....AA.--..ACA.............T.....TA.CG.....A............C-........C--A.A.-.TC..AC.ATGG...TTT...T----AT-CCA.C...G..T-T...CCG.CA--A..CC...TT...A**

**1aMch8a**  **----A.A.T....AA..TT.T...--..............G-A..GAT....AA.--...CA.............T.....TA..G..................CC......T.C--A.A.-.TC..AC.TTGG...TTT...T----GT-CGA.C...G..T-T...CC..CA--A..CC...TT...A**

**1aMch3a**  **--CAA.A.T....AA..TT.T...T-..............G-A..GAT....AA.--...CAA............T.....TA..G..................C-........C--A.A.-.TC..AC.TTGG...TTT...T----AT-CGA.C...G..T-T...CCG.CA--A..CC...TT...A**

**1aMch13a**  **--CAA.A.T....AA..TT.T...T-..............G-A..GAT....AA.--...CAA............T.....TA..G..................C-........C--A.A.-.TC..AC.TTGG...TTT...T----AT-CGA.C...G..T-T...CCG.CA--A..CC...TT...A**

**1b'Hch2**  **------------.AA..ATCA.A.C.......GC..TC.T.TT..G.TGG.C.A-----.CA.............T...T.T..CG.....-.A.A........A-..T.A...C--G.A.-ATGGTTCC.AGG...TTT...T----AT-CGA.G...T...-T...CC..C---...C........C-**

**1b'Hch8**  **------------.AA..ATCA.A.C.......GC..TC.T.TT..G.TGG.C.A-----.CA.............T...T.T..CG.....-.A.A........A-..T.A...C--G.A.-ATGGTTCC.AGG...TTT...T----AT-CGA.G...T...-T...CC..C---...C........C-**

**1b'Hch4**  **------------.AA..ATCA.A.C.......GC..TC.T.TT..G..GG.C.A-----.CA.............T...T.T..CG.....-.A.A........A-..T.A...C--G.A.-ATG.TTCC.AGG...TTT...T----AT-CGA.G...T...-T...CC..CA--...C........C-**

**1b'Hch12**  **------------.AA..ATCA.A.C.......GC..TC.T.TT..G..GG.C.A-----.CA.............T...T.T..CG.....-.A.A........A-..T.A...C--G.A.-ATG.TTCC.AGG...TTT...T----AT-CGA.G...T...-T...CC..CA--...CC.....A..C**

**1b'Hch11**  **------------.AA..ATCA.A.C.......GC..TC.T.TT..G..GG.C.A-----.CA.............T...T.T..CG.....-.A.A........A-..T.A...C--G.A.-ATG.TTCC.AGG...TTT...T----AT-CGA.G...T...-T...CC..CA--...CC.....A..C**

**1b'Hch3**  **------------.AA..ATCA.A.C.......GC..TC.T.TT..G..GG.C.A-----.CA.............T...T.T..CG.....-.A.A........A-..T.A...C--G.A.-ATG.TTCC.AGG...TTC...T----AT-CGA.G...T...-T...CC..CA--...CC.....A..C**

**1b'Hch22**  **------------.AA..ATCA.A.C.....G.GC..TC.T.TT..G..GG.C.A-----.CA.............T...T.T..CG.....-.A.A........A-..T.A...C--G.A.-ATG.TTCC.AGG...TT-...T----AT-CGA.G...T...-T.N.CC..CA--...CC.....AC--**

**1b'Hch21**  **------------.AA..ATCA.A.C.......GC..TC.T.TT..G..GG.C.A-----.CA.............T...T.T..CG.....-.A.A........A-..T.A...C--G.A.-ATG.TTCC.AGG...TTT...T----AT-CGA.G...TT..-T...CC..CA--...CC.....A..C**

**1b'H**

**1b'Hch6**  **------------.AA..A.CA.A.C.......GC..TC.T.TT..G..GG.C.A-----.CA.............T...T.T..CG.....-.A.A........A-..T.A...C--G.A.-ATG.TTCC.AGG...TTT...T----AT-CGA.G...T...-T...CC..CA--...CC.....A..C**

**1b'Hch5**  **------------.AA..ATCA.A.C.......GC..TC.T.TT..G..GG.C.A-----.CA.............T...T.T..CG.....-.A.A........A-..T.A...C--G.A.-ATG.TTCC.AGG...TTT...T----AT-CGA.G...T...-T...CT..CA--...CC.....A..C**

**1b'Hfa2**  **------------.AA..ATCA.A.C.......GC..TC.T.TT..G..GG.C.A-----.CA.............T...T.T..CG.....-.A.A........A-..T.A...C--G.A.-ATG.TTCC.AGG..GTTT...T----AT-CGA.G...T...-T...CC..C---...CC.....A..C**

**1b'Hch9**  **------------.AA..ATCA.A.C.......GC..TC.T.TT..G.TGG.C.A-----.CA.............T...T.T..CG.....-.A.A........A-..T.A...C--G.A.-ATGATTC..AGG...TTT...T----AT-CGA.G...T...-T...CC..C----..CC.....A..C**

**1b'Hch23**  **------------.AA..ATCA.A.C.......GC..TC.T.TT..G.TGG.C.A-----.CA.............T...T.T..CG.....-.A.A........A-..T.A...C--G.A.-ATGATTC..AGG...TTT...T----AT-CGA.G...T...-T...CC..C----..CC.....A..C**

**1b'HfaN4b**  **--AAA.A.T..T.AA..ATCA.A.C.......GC..CT.T---..G..GG.C.A-----.CA.............T...T.T..CG.....-.A.A........A-..T.A...C--G.A.-ATGATTC..AGG...TTT...T----AT-CGA.G...T...-T...CC..C----..CC.....A..C**

**1b'HfaP1b**  **--AAA.A.T..T.AA..ATCA.A.C.T.....G...CT.T---..G..GG.C.A-----.CA.............T...T.T..CG.....-.A.A........A-..T.A...C--G.A.-ATGATTC..AGG...TTT...T----AT-CGA.G...T...-T...CC..C----..CC.....A..C**

**1b'Hfa4b**  **--AAA.A.T..T.AA..ATCA.A.C.T.....G...CT.T---..G..GG.C.A-----.CA.............T...T.T..CG.....-.A.A........A-..T.A...C--G.A.-ATGATTC..AGG...TTT...T----AT-CGA.G...T...-T...CC..C----..CC.....A..C**

**1b'Hfa8b**  **--AAA.A.T..T.AA..ATCA.A.C.T.....G...CT.T---..G..GG.C.A-----.CA.............T...T.T..CG.....-.A.A........A-..T.A...C--G.A.-ATG.TTCC.AGG..GTTT...T----AT-CGA.G...T...-T...CC..C----..CC.....A..C**

**1b'HchP9b**  **--AAA.A.T..T.AA..ATCA.A.C.T.....G...CT.T---..G..GG.C.A-----.CA.............T...T.T..CG.....-.A.A........A-..T.A...C--G.A.-ATG.TTCC.AGG..GTTT...T----AT-CGA.G...T...-T...CC..C----..CC.....A..C**

**1b'HfaP10b** **--AAA.A.T..T.AA..ATCA.A.C.T.....G...CT.T---..G..GG.C.A-----.CA.............T...T.T..CG.....-.A.A........A-..T.A...C--G.A.-ATGATTC..AGG...TTT...T----AT-CGA.G...T...-T...CC..C----..CC.....A..C**

**1b'Hfa8a**  **---AA.A.T..T.AA..ATCA.A.C.T.....G...CT.---T..G..GG.C.A-----.CA.............T...T.T..CG.....-.A.A........A-..T.A...C--G.A.-ATGATTC..AGG...TTT...-----AT-CGA.G...T...-T...CC..C----..CC.....A..C**

**1b'Hfa17b**  **---AA.A.T..T.AA..ATCA.A.C.T.....G...CT.---T..G..GG.C.A-----.CA.............T...T.T..CG.....-.A.A........A-..T.A...C--G.A.-ATGATTC..AGG...TTT...T----AT-CGA.G...T...-T...CC..C----..CC.....A..C**

**1b'Hfa2b**  **---AA.A.T..T.AA..ATCA.A.C.T.....G...CT.---C..G..GGC..A-----.CA.............T...T.T..CG.....-.A.A........A-..T.A...C--G.A.-ATGATTC..AGG...TTT...T----AT-CGA.G...T...-T...CC..C----..CC.....A..C**

**1bHfaN4**  **----------.TCAA..ATCA.A.C.......GC..TC.T.TT..G..GG.C.A-----.CA.............T...T.T..C......-.A.A......C.A-...A....C--G.A.-ATGATTC-.AGC..CTTT...TT----TTCAA.C...G...-T...CC..C---T..CC....T.---**

**1bHfaP1**  **----------.TCAA..ATCA.A.C.......GC..TC.T.TT..G..GG.C.A-----.CA.............T...T.T..C......-.A.A......C.A-...A....C--G.A.-ATGATTC-.AGC..CTTT...TT----TTCAA.C...G...-T...CC..C---T..CC....T.---**

**1bH**

**1bHfaP4**  **----------.TCAA..ATCA.A.C.......GC..TC.T.TT..G..GG.C.A-----.CA.............T...T.T..C......-.A.A......C.A-...A....C--G.A.-ATGATTC-.AGC..CTTT...TT----TTCAA.C...G...-T...CC..C---T..CC....T.---**

**1bHfaP8**  **----------.TCAA..ATCA.A.C.......GC..TC.T.TT..G..GG.C.A-----.CA.............T...T.T..C......-.A.A......C.A-...A....C--G.A.-ATGATTC-.AGC..CTTT...TT----TTCAA.C...G...-T...CC..C---T..CC....T.---**

**1bHchP9**  **----------.TCAA..ATCA.A.C.......GC..TC.T.TT..G..GG.C.A-----.CA.............T...T.T..C......-.A.A......C.A-...A....C--G.A.-ATGATTC-.AGC..CTTT...TT----TTCAA.C...G...-T...CC..C---T..CC....T.---**

**1bHfaP10**  **----------.TCAA..ATCA.A.C.......GC..TC.T.TT..G..GG.C.A-----.CA.............T...T.T..CG.....-.A.A......C.A-...A....C--G.A.-ATGATTC-.AGC..CTTT...TT----TTCAA.C...G...-T...CC..C---T..CC....T.---**

**1bHfa2a**  **----------.TCAA..ATCA.A.C.......GC..TC.T.TT..G..GG.C.A-----.CA.............T...T.T..C......-.A.A......C.A-...A....C--G.A.-ATGATTC-.AGC..CTTT...TT----TTCAA.C...G...-T...CC..C---T..CC....T.---**

**1bHfa8**  **----------.TCAA..ATCA.A.C.......GC..TC.T.TT..G..GG.C.A-----.CA.............T...T.T..C.....--.A.A......C.A-...A....C--G.A.-ATGATTC-.AGC..CTTT...TT----TTCAA.C...G...-T...CC..C---T..CC....T.---**

**1bHfa17**  **----------.TCAA..ATCA.A.C.......GC..TC.T.TT..G..GG.C.A-----.CA.............T...T.T..C......-.A.A......C.A-...A....C--G.A.-ATGATTC-.AGC..CTTT...TT----TTCAA.C...G...-T...CC..C---T..CC....T.---**

**2aHfa2_**  **----------------T.TCA.AAC.TA.TG..TT.TC.TCAAA.AATA..CAT-----.CCAGCT...A.AT..T...AAT.AA.TC...............TATG...CATA.AGA.A.A.T.TGTC.AT..T..GTTAGC.GC-GGTTGAACC.CCC...TT...TT..-----.GG..C..G.C.GAAATTTTCGGAACAAG**

**2aHch8**  **----------------T.TCA.AAC.TA.TG..TT.TC.TCAAA.AATA..CAT-----.CCAGCT...A.AT..T...AAT.AA.TC.A.............TATG...CATA.AGA.A.A.T.TGTC.AT..T..GTTAGC.GC-GGTTGAACC.CCC...TT...TT..-----.GG..C..G.C.GAAATTTTCGGAACAAG**

**2aHfa17b_**  **----------------T.TCA.AAC.TA.TG..TT.TC.TCAAA.AATA..CAT-----.CCAGCT...A.AT..T...AAT.AA.TC...............TATG...CATA.AGA.A.A.T.TGTC.AT..T..GTTAGC.GC-GGTTGAACC.CCC...TT...TT..-----.GG..C..G.C.GAAATTTTCGGAACAAG**

**2aHch2**  **----------------T.TCA.AAC.TA.TG..TT.TC.TCAAA.AATA..CAT-----.CCAGCT...A.AT..T...AAT.AA.TC...............TATG...CATA.AGA.A.A.T.TGTC.AT..T..GTTAGC.GC-GGTTGAACC.CCC...TT...TT..-----.GG..C..G.C.GAAATTTTCGGAACAAG**

**2aHch3**  **----------------T.TCA.AAC.TA.TG..TT.TC.TCAAA.AATA..CAT-----.CCAGCT...A.AT..T...AAT.AA.TC...............TATG...CATA.AGA.A.A.T.TGTC.AT..T..GTTAGC.GC-GGTTGAACC.CCC...TT...TT..-----.GG..C..G.C.GAAATTTTCGGAACAAG**

**2aHch4**  **----------------T.TCA.AAC.TA.TG..TT.TC.TCAAA.AATA..CAT-----.CCAGCT...A.AT..T...AAT.AA.TC...............TATG...CATA.AGA.A.A.T.TGTC.AT..T..GTTAGC.GC-GGTTGAACC.CCC...TT...TT..-----.GG..C..G.C.GAAATTTTCGGAACAAG**

**2aMH**

**2aHch6**  **----------------T.TCA.AAC.TA.TG..TT.TC.TCAAA.AATA..CAT-----.CCAGCT...A.AT..T...AAT.AA.TC...............TATG...CATA.AGA.A.A.T.TGTC.AT..T..GTTAGC.GC-GGTTGAACC.CCC...TT...TT..-----.GG..C..G.C.GAAATTTTCGGAACAAG**

**2aHch12**  **----------------T.TCA.AAC.TA.TG..TT.TC.TCAAA.AATA..CAT-----.CCAGCT...A.AT..T...AAT.AA.TC...............TATG...CATA.AGA.A.A.T.TGTC.AT..T..GTTAGC.GC-GGTTGAACC.CCC...TT...TT..-----.GG..C..G.C.GAAATTTTCGGAACAAG**

**2aHch9**  **----------------T.TCA.AAC.TA.TG..TT.TC.TCAAA.AATA..CAT-----.CCAGCT...A.AT..T...AAT.AA.TC...............TATG...CATA.AGA.A.A.T.TGTC.AT..T..GTTAGC.GC-GGTTGAACC.CCCG..TT...TT..-----.GG..C..G.C.GAAATTTTCGGAACAAG**

**2aMfa7**  **----------------T.TCA.AAC.TA.TG..TT.TC.TCAAA.AATC..CAT-----.C-AGCT...A.AT..T...AAT.AA.TC...............TATG...CATA.AGA.A.A.T.TGTC.AT..T..GTTAGC.GC-GGTTGAACC.CCC...TT...TT..-----.GG..C..G.C.GAAATTTTCGGAACAG-**

**2aMch6a**  **----------------T.TCA.AAC.TA.TG..TT.TC.TCAAA.AATC..CAT-----.C-AGCT...A.AT..T...AAT.AA.TC...............TATG...CATA.AGA.A.A.T.TGTC.AT..T..GTTAGC.GC-GGTTGAACC.CCC...TT...TT..-----.GG..C..G.C.GAAATTTTCGGAACAG-**

**2aMch10b**  **----------------T.TCA.AAC.TA.TG..TT.TC.TCAAA.AATC..CAT-----.C-AGCT...A.AT..T...AAT.AA.TC...............TATG...CATA.AGA.A.A.T.TGTC.AT..T..GTTAGC.GC-GGTTGAA.CACCC...TT...TT..-----AGG..C..G.C.GAAATTTTCGGAACAAG**

**2aMch8c**  **----------------T.TCA.AAC.TA.TG..TT.TC.TCAAA.AATC..CAT-----.CCAGCT...A.AT..T...AAT.AA.T................TATG...CATA.AGA.A.A.T.TGTC.AT..T..GTTAGC.GC-GGTTGAA.CACCC...TT...TT..-----AGG..C..G.C.GAAATTTTCGGAACAAG**

**2aMch8d**  **----------------T.TCA.AAC.TA.TG..TT.TC.TCAAA.AATC..CAT-----.CCAGCT.....A...T...AAT.AA.TC...............TATG...CATA.AGA.A.A.T.TGTC.AT..T..GTTAGC.GC-GGTTGAACC.CCC...TT...TT..-----.GG..C..G...GAAATTTTCGGAACAAG**

**2aMfa3b**  **----------------T.TCA.AAC.TA.TG..TT.TC.TCAAA.AATC..CAT-----.CCAGCT.....A...T...AAT.AA.TC...............TATG...CATA.A.A.A.A.T.TGTC.AT..T..TTTAGC.GC-GGTTGAACC.CCC...TT...TT..-----.G..C..G.C.GAAATTTTCGGAACAAGG**

**2aMfa4b**  **----------------T.TCA.AAC.TA.TG..TT.TC.TCAAA.AATC..CAT-----.CCAGCT...A.AT..T...AAT.AA.TC...........A...TATG...CATA.AGA.A.A.TATGTC.AT..T..GTTAGC.GC-AGGCGAACC.CCC...TT...TT..-----AG..C..G.C.GAAATCTTCGGAACAAGG**

**2aMfa5b**  **----------------T.TCA.AAC.TA.TG..TT.TC.TCAAA.AATC..CAT-----.CCAGCT...A.AT..T...AAT.AA.TC...........A...TATG...CATA.AGA.A.A.TATGTC.AT..T..GTTAGC.GC-AGGCGAACC.CCC...TT...TT..-----AG..C..G.C.GAAATCTTCGGAACAAGG**

**2aMch10a**  **----------------T.TCA.AAC.TA.TG..TT.TC.TCAAA.AATC..CAT-----.C-AGCT...A.AT..T...AAT.AA.T................TATG...CATA.AGA.A.A.T.TGTC.AT..T..GTTAGC.GC-AGGCGAACC.CCC...TT...TT..-----.G..C..G.C.GAAATTTTCGGAACAAGG**

**2aMfa4a**  **----------------T.TCA.AAC.TA.TG..TT.TC.TCAAA.AATC..CAT-----.C-AGCT.....AT..T...AAT.AA.TC...............TATG...CATA.AGA.A.A.TATGTC.AT..T..GTTAGC.GC-AGGCGAACC.CCC...TT...TT..-----.G..C..G.C.GAAATTTTCGGAACAAGG**

**2aMfa5a**  **----------------T.TCA.AAC.TA.TG..TT.TC.TCAAA.AATC..CAT-----.C-AGCT.....AT..T...AAT.AA.TC...............TATG...CATA.AGA.A.A.TATGTC.AT..T..GTTAGC.GC-AGGCGAACC.CCC...TT...TT..-----.G..C..G.C.GAAATTTTCGGAACAAGG**

**2aMch6b**  **----------------T.TCA.AAC.TA.CG..TT.TC.TCAAA.AATC..CAT-----.CCAGCT.....A...T...AAT.AA.TC...............TATG...CATA.AGA.A.A.TATGTC.AT..T..GTTAGC.GC-AGGCGAACC.CCC...TT...TT..-----.G..C..G.C.GAAATTTTCGGAACAAGG**

**2aMch7**  **----------------T.TCA.AAC.TA.CG..TT.TC.TCAAA.AATC..CAT-----.CCAGCT.....A...T...AAT.AA.TC...............TATG...CATA.AGA.A.A.TATGTC.AT..T..GTTAGC.GC-AGGCGAACC.CCC...TT...TT..-----.G..C..G.C.GAAATTTTCGGAACAAGG**

**2aMch8a**  **----------------T.TCA.AAC.TA.TG..CT.TC.TCAAA.AATC..CAT-----.CCAGCT.....A...T...AAT.AA.TC...............TATG...CATA.AGA.A.A.T.TGTC.AT..T..GTTAGC.GC-AGGCGAACC.CCC...TT...TT..-----.G..C..G.C.GAAATTTTCGGAACAAGG**

**2aMH**

**2aMfa3c**  **----------------T.TCA.AAC.TA.TG..TT.TC.TCAAA.AATC..CAT-----.CCAGCT.....A...T...AAT.AA.TC...............TATG...CATA.AGA.A.A.T.TGTC.AT..T..GTTAGC.GA-AGGCGAACC.CCC...TT...TT..-----.G..C..G...GAAATTTTCGGAACAAGG**

**2aMch8b**  **----------------T.TCA.AACCTA.TG..TT.TC.TCAAA.AATC..CAT-----.CCAGCT...A.AT..T...AAT.AA..C...............TATG...CATA.AGA.A.A.T.TGTC.AT..T..GTTAGC.GC-AGGCGAACC.CCC...TT...TT..-----AG..C..G.C.GAAATTTTCGGAACAAGG**

**2aMfa3a**  **----------------T.TCA.AAC.TA.TG..TT.TC.TCAAA.AATC..GAT-----.CTAGCT.....AT..T...AAT.AA.TC...............TATG...CATT.A.A.A.A.T.TGTC.AT..T..TTTAGC.GC-GGTTGAACC.CCC...TT...TT..-----.G..C..G.C.GAAATTTTCGGAACAAGG**

**2aHfa8**  **----------------T.TCA.AAC.TA.TG..TT.TC.TCAAA.AATA..CAT-----.CCAGCT...A.AT..T...AAT.AA.TC...............TATG...CATA.AGA.A-A.T.TGTC.AT..T..GTTAGC.GC-GGTTGAACC.CCC...TT...TT..-----.G..C..G.C.GAAATTTTCGGAACAAGG**

**2aHfaN4**  **----------------T.TCA.AAC.TA.TG..TT.TC.TCAAA.AATA..CAT-----.CCAGCT...A.AT..T...AAT.AA.TC...............TATG...CATA.AGA.A.A.T.TGTC.AT..T..GTTAGC.GC-GGTTGAACC.CCC...TT...TT..-----.G..C..G.C.GAAATTTTCGGAACAAGG**

**2aHfaP1**  **----------------T.TCA.AAC.TA.TG..TT.TC.TCAAA.AATA..CAT-----.CCAGCT...A.AT..T...AAN.AA.TC...............TATG...CATA.AGA.A.A.T.TGTC.AT..T..GTTAGC.GC-GGTTGAACC.CCC...TT...TT..-----.G..C..G.C.GAAATTTTCGGAACAAGG**

**2aHfaP4**  **----------------T.TCA.AAC.TA.TG..TT.TC.TCAAA.AATA..CAT-----.CCAGCT...A.AT..T...AAT.AA.TC...............TATG...CATA.AGA.A.A.T.TGTC.AT..T..GTTAGC.GC-GGTTGAACC.CCC...TT...TT..-----.G..C..G.C.GAAATTTTCGGAACAAGG**

**2aHfaP8**  **----------------T.TCA.AAC.TA.TG..TT.TC.TCAAA.AATA..CAT-----.CCAGCT...A.AT..T...AAT.AA.TC...............TATG...CATA.AGA.A.A.T.TGTC.AT..T..GTTAGC.GC-GGTTGAACC.CCC...TT...TT..-----.G..C..G.C.GAAATTTTCGGAACAAGG**

**2aHchP9**  **----------------T.TCA.AAC.TA.TG..TT.TC.TCAAA.AATA..CAT-----.CCAGCT...A.AT..T...AAT.AA.TC...............TATG...CATA.AGA.A.A.T.TGTC.AT..T..GTTAGC.GC-GGTTGAACC.CCC...TT...TT..-----.G..C..G.C.GAAATTTTCGGAACAAGG**

**2aHfaP10**  **----------------T.TCA.AAC.TA.TG..TT.TC.TCAAA.AATA..CAT-----.CCAGCT...A.AT..T...AAT.AA.TC...............TATG...CATA.AGA.A.A.T.TGTC.AT..T..GTTAGC.GC-GGTTGAACC.CCC...TT...TT..-----.G..C..G.C.GAAATTTTCGGAACAAGG**

**2aHch21**  **----------------T.TCA.AAC.TA.TG..TT.TC.TCAAA.AATA..CAT-----.CCAGCT...A.AT..T...AAT.AA.TC...............TATG...CATA.AGA.A.A.T.TGTC.AT..T..GTTAGC.GC-GGTTGAACC.CCC...TT...TT..-----.G..C..G.C.GAAATTTTCGGAACAAGG**

**2aHch22**  **----------------T.TCA.AAC.TA.TG..TT.TC.TCAAA.AATA..CAT-----.CCAGCT...A.AT..T...AAT.AA.TC...............TATG...CATA.AGA.A.A.T.TGTC.AT..T..GTTAGC.GC-GGTTGAACC.CCC...TT...TT..-----.G..C..G.C.GAAATTTTCGGAACAAGG**

**2aHch23**  **----------------T.TCA.AAC.TA.TG..TT.TC.TCAAA.AATA..CAT-----.CCAGCT...A.AT..T...AAT.AA.TC...............TATG...CATA.AGA.A.A.T.TGTC.AT..T..GTTAGC.GC-GGTTGAACC.CCC...TT...TT..-----.G..C..G.C.GAAATTTTCGGAACAAGG**

**2aHch11**  **----------------T.TCA.AAC.TA.TG..TT.TC.TCAAA.AATA..CAT-----.CCAGCT...A.AT..T...AAT.AA.TC...............TATG...CATA.AGA.A.A.T.TGTC.AT..T..GTTAGC.GC-GGTTGAACC.CCC...TT...TT..-----.GG.C..G.C.GAAATTTTCGGAACAAGG**

**2bMch1**  **---------------CCAT.AC.CTCA..TGT.TT.T..TCAAA.GAG...AAT------CT..CT.AT..AT..T...AAT.CA..................TATG...CATAAAG..A.AAT.TTTTTAA...CTTTTAGATCT-TTTTGAA....CC...ATT..TT....--AAT..CTG.CC.ACAATTTCATAAGT---A**

**2bM**

**2bMch3**  **---------------CCAT.AC.CTCA..TGT.TT.T..TCAAA.GAG...AAT------CT..CT.AT..AT..T.....T.CA..................TATG...CATAAAG..A.AAT.TTTTTAA...CTTTTAGATCT-TTTTGAA....CC...ATT..TT....---AT..CTG.CC.ACAATTTCATAAGT---A**

**2bMch5**  **---------------CCAT.AC.CTCA..TGT..T.T..TCAAA.GAG...AAT------CT..CT.AT..AT..T.....T.CA..................TATG...CATAAAG..A.AAT.TTTTTAAG..CTTTTAGATC--TTTTGAA....CC...ATT..TT....---AT..CTG.C..ACAATTTCATAAGT---A**

**2bMch9**  **---------------CCAT.AC.CTCA..TGT.TT.T..T.AAA.GAG...A.T------CT..CT.A...AT..T...AAT.CA..................TATG...CATAAAG..A.AAT.TTTTTAA...CTTTTAGATCT-TTTTGAAT...CC...ATT..TT....TTAAT..CTG.CC.ACAATTTCATAAGT---A**

**2bMch2**  **---------------CGAT.AC.CTCA..TGT..T.T..TCAAA.GAG...AAT------CT..CT.A...AT..T...AAT.CA..................TATG...CATAAAG..A.AAA.TTTTTAA...CTTTTAGATCT-TTTTGAA....CC..TATT..TT....---AT..CTG.CC.ACAATTTCATAAGT---A**

**2bMch4**  **---------------CCAT.AC.CTCA..TGT..T.T..TCAAA.GAG...AAT------CT..CT.AT..AT..T.....T.CA..................TATG...CATAAAG..A.AAT.TTTTTAA...CTTTTAGATCT-TTTTGAAT...CC...ATT..TT....---AT..CTG.CC.ACAATTTCATAAGT---A**

**2bMch7**  **---------------CCAT.AC.CTCA..TGT..T.T..TCAAA.GAG...A.T------CT..CT.AT..AT..T.....T.CA..................TATG...CATAAAG..A.AAT.TTTTTAAG..CTTTTAGATCT-TTTTGAA....CC...ATT..TT....---AT..CTG.CC.ACAATTTCATAAGT---A**

Figure S3. Alignment of 1a, 1b, 1b’, 1c, 1d, 2a and 2b monomers from *M. fallax* and *M. chitwoodi*. Monomers are extracted from monomeric and HOR arrays using KSA algorithm [26]. All monomers are compared with first sequence and positions identical to the first sequence are shown with dot. Monomer group are indicated on the right side. Monomer sequence are deposited in EMBL data bank under accession numbers:JX186757 - JX186849 and JX186878 - JX186996. Box 1 is shaded with yellow. Detail description of satellite monomers are indicated below:

1cHch_n_-1c monomers from chitwoodi HOR arrays (H_1c_ch_n_, H_u_ch_n_)

1cHfa_n_ -1c monomers from fallax HOR arrays (H_1c_fa_n_, H_u_fa_n_)

1cMch_n_-1c monomers from chitwoodi monomeric arrays (M_1c_ch_n_)

1cMfa_n_ -1c monomers from fallax monomeric arrays (M_1c_fa_n_)

1dHch_n_-1d monomers from chitwoodi HOR arrays (H_1c_ch_n_, H_u_ch_n_)

1dHfa_n_ -1d monomers from fallax HOR arrays (H_1c_fa_n_, H_u_fa_n_)

1dMch_n_-1d monomers from chitwoodi monomeric arrays (M_1c_ch_n_)

1dMfa_n_ -1d monomers from fallax monomeric arrays (M_1c_fa_n_)

1aHch_n_-1a monomers from chitwoodi HOR arrays (H_1c_ch_n_, H_u_ch_n_)

1aHfa_n_ -1a monomers from fallax HOR arrays (H_1c_fa_n_, H_u_fa_n_)

1aMch_n_-1a monomers from chitwoodi monomeric arrays (M_1a_ ch_n_)

1aMfa_n_ -1a monomers from fallax monomeric arrays (M_1a_ fa_n_)

1bHch_n_-1b monomers from chitwoodi HOR arrays (H_1c_ch_n_, H_u_ch_n_)

1bHfa_n_ -1b monomers from fallax HOR arrays (H_1c_fa_n_, H_u_fa_n_)

1b’Hch_n_-1b monomers from chitwoodi HOR arrays (H_1c_ch_n_, H_u_ch_n_)

1b’Hfa_n_ -1b monomers from fallax HOR arrays (H_1c_fa_n_, H_u_fa_n_)

2aHch_n_-2a monomers from chitwoodi HOR arrays (H_1c_ch_n_, H_u_ch_n_)

2aHfa_n_ -2a monomers from fallax HOR arrays (H_1c_fa_n_, H_u_fa_n_)

2aMch_n_-2a monomers from chitwoodi monomeric arrays (M_2a_ ch_n_)

2aMfa_n_ -2a monomers from fallax monomeric arrays (M_2a_ fa_n_)

2bMch_n_-2b monomers from chitwoodi monomeric arrays (M_2b_ ch_n_)
