# Supplementary material for: Conserved DNA Motifs, Including the CENP-B Box-like, Are Possible Promoters of Satellite DNA Array Rearrangements in Nematodes
Source: PLoS One. 2013 Jun 27;8(6):e67328. doi: 10.1371/journal.pone.0067328 (PMC3694981; doi:10.1371/journal.pone.0067328)
Supplement: Figure S4 — Alignment of Box 1-containing sequences extracted from unassembled part of M. incognita sequenced genome. All sequences are compared with first sequence and positions identical to the first sequence are shown with dot. Sequences are deposited in EMBL data bank under accession numbers: KC968979–KC969073. Box 1 is shaded with yellow. (DOC) [file pone.0067328.s004.doc]

10 20 30 40 50 60 70 80 90 100 110 120 130 140 150 160 170 180 190 200

....|....|....|....|....|....|....|....|....|....|....|....|....|....|....|....|....|....|....|....|....|....|....|....|....|....|....|....|....|....|....|....|....|....|....|....|....|....|....|....|

**RN0AAB125YE10FM1**  **---------------------------------------------------------------------------------------------------------------------------------------------------------------------------------CCA-----------AGGCTGGTC**

**RN0AAB3YE08AHM1**  **---------------------------------------------------------------------------------------------------------------------------------------------------------------------------------...-----------.........**

**RN0AAB140YN05FM1**  **---------------------------------------------------------------------------------------------------------------------------------------------------------------------------------...-----------.........**

**RN0AAB414YN09FM1**  **----------------------------------------------------------------------------------------------------------------------------------------------------------------------------------..-----------.........**

**RN0AAB545YM04AHM1** **----------------------------------------------------------------------------------------------------------------------------------------------------------------------------------..-----------.........**

**RN0AAA130YM16FM1**  **----------------------------------------------------------------------------------------------------------------------------------------------------------------------------------..-----------.........**

**RN0AAA519YK05RM1**  **--------------------------------------------------------------------------------------------------------------------------------------------------------------------------------------------------------**

**RN0AAB125YE10FM1**  **--------------------------------------------------------------------------------------------------------------------------------------------------------------------------------------------------------**

**RN0AAB3YE08AHM1**  **--------------------------------------------------------------------------------------------------------------------------------------------------------------------------------------------------------**

**RN0AAB140YN05FM1**  **--------------------------------------------------------------------------------------------------------------------------------------------------------------------------------------------------------**

**RN0AAB414YN09FM1**  **--------------------------------------------------------------------------------------------------------------------------------------------------------------------------------------------------------**

**RN0AAB545YM04AHM1** **--------------------------------------------------------------------------------------------------------------------------------------------------------------------------------------------------------**

**RN0AAA130YM16FM1**  **--------------------------------------------------------------------------------------------------------------------------------------------------------------------------------------------------------**

**RN0AAA519YK05RM1**  **--------------------------------------------------------------------------------------------------------------------------------------------------------------------------------------------------------**

**RN0AAB176YD12FM1**  **--------------------------------------------------------------------------------------------------------------------------------------------------------------------------------------------------------**

**RN0AAA286YA16FM1**  **--------------------------------------------------------------------------------------------------------------------------------------------------------------------------------------------------------**

**RN0AAA179YC21RM1**  **--------------------------------------------------------------------------------------------------------------------------------------------------------------------------------------------------------**

**RN0AAA756YE07FM1**  **--------------------------------------------------------------------------------------------------------------------------------------------------------------------------------------------------------**

**RN0AAA591YF08RM1**  **--------------------------------------------------------------------------------------------------------------------------------------------------------------------------------------------------------**

**RN0AAA538YB16RM1**  **--------------------------------------------------------------------------------------------------------------------------------------------------------------------------------------------------------**

**RN0AAB458YP20AHM1** **--------------------------------------------------------------------------------------------------------------------------------------------------------------------------------------------------------**

**RN0AAA669YI24RM1**  **--------------------------------------------------------------------------------------------------------------------------------------------------------------------------------------------------------**

**RN0AAA87YA19FM1**  **--------------------------------------------------------------------------------------------------------------------------------------------------------------------------------------------------------**

**RN0AAB380YP14FM1**  **--------------------------------------------------------------------------------------------------------------------------------------------------------------------------------------------------------**

**RN0AAB423YM10FM1**  **--------------------------------------------------------------------------------------------------------------------------------------------------------------------------------------------------------**

**RN0AAB147YJ10FM1**  **--------------------------------------------------------------------------------------------------------------------------------------------------------------------------------------------------------**

**RN0AAA64YH05FM1**  **--------------------------------------------------------------------------------------------------------------------------------------------------------------------------------------------------------**

**RN0AAB176YD12FM1**  **----------------------------------------------------------------------------------------------------------------------------------------------------------------------------------T.CACTTCG-GTT..TT.T..T**

**RN0AAA286YA16FM1**  **------------------------------------------------------------------------------------------------------------------------------------------------------------------------------------------------........**

**RN0AAA179YC21RM1**  **---------------------------------------------------------------------------------------------------------------------------------------------------------------------------------TTTGGTAATT-CTTC..T..-..**

**RN0AAA756YE07FM1**  **---------------------------------------------------------------------------------------------------------------------------------------------------------------------------------TTTGGTAATT-CTTC..T..-..**

**RN0AAA591YF08RM1**  **--------------------------------------------------------------------------------------------------------------------------------------------------------------------------------------------------------**

**RN0AAA538YB16RM1**  **--------------------------------------------------------------------------------------------------------------------------------------------------------------------------------------------------------**

**RN0AAB458YP20AHM1** **-------------------------------------------------------------------------------------------------------------------------------------------------------------------------------TCT.CGGTAATA-GTTT.TAATTCG**

**RN0AAA669YI24RM1**  **-------------------------------------------------------------------------------------------------------------------------------------------------------------------------------TC.TTGGTAATA-GTTT..AATTCG**

**RN0AAA87YA19FM1**  **-------------------------------------------------------------------------------------------------------------------------------------------------------------------------------TATTTGGTAATA-GTTT..AA.TCG**

**RN0AAB380YP14FM1**  **-------------------------------------------------------------------------------------------------------------------------------------------------------------------------------AAATTGGTAATA-GTTT..AATTCG**

**RN0AAB423YM10FM1**  **-------------------------------------------------------------------------------------------------------------------------------------------------------------------------------AAATTGGTAATA-GTTT..AATTCG**

**RN0AAB147YJ10FM1**  **---------------------------------------------------------------------------------------------------------------------------------------------------------------------------------TTTGGTAATA-CTTT..AA.-..**

**RN0AAA64YH05FM1**  **---------------------------------------------------------------------------------------------------------------------------------------------------------------------------------AATGGTAATA-GTTT.....AC.**

**RN0AAB311YB19AHM1** **--------------------------------------------------------------------------------------------------------------------------------------------------------------------------------------------------------**

**RN0AAB44YH23AHM1**  **--------------------------------------------------------------------------------------------------------------------------------------------------------------------------------------------------------**

**RN0AAA717YI15RM1**  **-------------------------------------------------------------------------------------------------------------------------------------------------------------------------------G-TTTGGTAATT-CCA.........**

**RN0AAB125YA16FM1**  **----------------------------------------------------------------------------------------------------------------------------------------------------------------------------------ACGGTAATNAGTTT..AATTCG**

**RN0AAB437YE21AHM1** **----------------------------------------------------------------------------------------------------------------------------------------------------------------------------------ACGGTAATA-GTTT..AATTCG**

**RN0AAB266YH16AHM1** **------------------------------------------------------------------------------------------------------------------------------------------------------------------------------------GGTAATA-GTTT..AATTCG**

**RN0AAA131YF02RM1**  **----------------------------------------------------------------------------------------------------------------------------------------------------------------------------------ATGGTAATA-GTTT..AATTCG**

**RN0AAA130YE05RM1**  **----------------------------------------------------------------------------------------------------------------------------------------------------------------------------------ATGGTAATA-GTTT..AATTCG**

**RN0AAA579YK24RM1**  **--------------------------------------------------------------------------------------------------------------------------------------------------------------------------------------------------------**

**RN0AAB100YP08AHM1** **---------------------------------------------------------------------------------------------------------------------------------------------------------------------------------------AATA-GTTT..AATTCG**

**RN0AAB180YL08FM1**  **----------------------------------------------------------------------------------------------------------------------------------------------------------------------------------TCGGTAATA-GTTT..AATTCG**

**RN0AAA175YL12RM1**  **-------------------------------------------------------------------------------------------------------------------------------------------------------------------------------A-TGGGGTAATA-GTTT..AATTCG**

**RN0AAB484YE18FM1**  **---------------------------------------------------------------------------------------------------------------------------------------------------------------------------------.TCGGTAATA-GTTT..AATTCG**

**RN0AAA316YE18RM1**  **----------------------------------------------------------------------------------------------------------------------------------------------------------------------------------TTGGTAATA-GTTT..AATTCG**

**RN0AAA14YA24RM1**  **----------------------------------------------------------------------------------------------------------------------------------------------------------------------------------TTGGTAATA-GTTT..AATTCG**

**RN0AAA691YK01RM1**  **------------------------------------------------------------------------------------------------------------------------------------------------------------------------------------GGTAATA-GTTT..--CTGA**

**RN0AAB526YI05AHM1** **------------------------------------------------------------------------------------------------------------------------------------------------------------------------------------GGTAATA-GTTT..--CTGA**

**RN0AAB490YN21AHM1** **---------------------------------------------------------------------------------------------------------------------------------------------------------------------------------TTTGGTAATT-CTTT..-.T...**

**RN0AAA132YI06RM1**  **----------------------------------------------------------------------------------------------------------------------------------------------------------------------------------------------------T...**

**RN0AAB289YD03FM1**  **---------------------------------------------------------------------------------------------------------------------------------------------------------------------------------TTTGGTAATT-CCA...-CT.GT**

**RN0AAA727YI23RM1**  **---------------------------------------------------------------------------------------------------------------------------------------------------------------------------------TTGGGTAATA-CCA...-CT.GT**

**RN0AAA90YK10FM1**  **---------------------------------------------------------------------------------------------------------------------------------------------------------------------------------TAGGGTAATT-CCA...A.T...**

**RN0AAB20YB17AHM1**  **----------------------------------------------------------------------------------------------------------------------------------------------------------------------------------ACGGTAATT-CCA...A.T...**

**RN0AAB577YG05AHM1** **---------------------------------------------------------------------------------------------------------------------------------------------------------------------------------ATGGGTAATT-CCA...A.T...**

**RN0AAB48YA06AHM1**  **--------------------------------------------------------------------------------------------------------------------------------------------------------------------------------------------------------**

**RN0AAA133YD03FM1**  **--------------------------------------------------------------------------------------------------------------------------------------------------------------------------------------------------------**

**RN0AAA542YE07RM1**  **---------------------------------------------------------------------------------------------------------------------------------------------------------------------------------T.CGGTAATT-CCA...A.T...**

**RN0AAA601YB08RM1**  **-------------------------------------------------------------------------------------------------------------------------------------------------------------------------------A-.AGGGTAATT-CCA...A.T...**

**RN0AAA281YH13FM1**  **-------------------------------------------------------------------------------------------------------------------------------------------------------------------------------A-.AGGGTAATT-CCA...A.T...**

**RN0AAA699YB04FM1**  **-------------------------------------------------------------------------------------------------------------------------------------------------------------------------------ATT.CGGTAATT-CTTC..-.T...**

**RN0AAA437YA19RM1**  **-------------------------------------------------------------------------------------------------------------------------------------------------------------------------------AG.A.AAAGACTTTGG.AT..TTGA**

**RN0AAA499YO02FM1**  **---------------------------------------------------------------------------------------------------------------------------------------------------------------------------------.A.AAAGACTTTGG.AT..TTGA**

**RN0AAA377YF05FM1**  **-------------------------------------------------------------------------------------------------------------------------------------------------------------------------------AG.A.AAAGACTTTGG.AT..TTGA**

**RN0AAA222YD17RM1**  **--------------------------------------------------------------------------------------------------------------------------------------------------------------------------------------------------------**

**RN0AAB173YC20AHM1** **--------------------------------------------------------------------------------------------TTTGATACAGTTTTTTAACGCTCTTCGAAATGATATATAGAACTTCTATCTCAAATTTAAATTCGCGAATTAT-AATTTATACAAAA.TTTCATGTAATTAAAAATGT**

**RN0AAB161YD24FM1**  **--------------------------------------------------------------------------------------------------------------------------------------------------------------------------------------------------------**

**RN0AAB55YM18AHM1**  **--------------------------------------------------------------------------------------------------------------------------------------------------------------------------------------------------------**

**RN0AAA481YC13RM1**  **------------------------------------------------------------------------------------------------------------------------------------------------------------GTGAATTATAAAATTATACAAAT.TTTCATGTAATTAAAAAT.T**

**RN0AAA81YK04RM1**  **--------------------------------------------------------------------------------------------------------------------------------------------------------------------------------------------------------**

**RN0AAA229YJ17FM1**  **--------------------------------------------------------------------------------------------------------------------------------------------------------------------------------------------------------**

**RN0AAA222YD17FM1**  **-----------------------------TTACGTTAATTCCTGGTGTACATTCAACTTGAAAATTAAATTGAAAAACAATTACCTGGCTTTTTTTGATACAGTTTTTAACGCTCTTTCGAATGATATATAGAACTTCTATCTCAAATTTAAATTCGCGAATTAT-AAATTATACAAAA.TTTCATGTAATTAAAAATGT**

**RN0AAA303YG05RM1**  **-------------------------------------------------------------------------------------------------------------------------------------------ATTTCAAATTTAAATTCGCGAATTAT-AAATTATACAAAA.TTTCATGTAATTAAAAAT.T**

**RN0AAA32YB09FM1**  **--------------------------------------------------------------------------------------------------------------------------------------------------------------------------------------------------------**

**RN0AAA474YI20RM1**  **---------------------TTTCCAACTTACGTTAACTCCTGGTGTACATTCAACTTGAAAATTAAATTGAAAAACAATTACCTGGCTTTTTTTGATACAGTTTTTAACGCTCTTTCGAATGATATATAGAACTTCTATCTCAAATTTAAATACGCGAATTAT-AAATTATACAAAT.TTTCATGTAATTAAAAAT.T**

**RN0AAA373YI20FM1**  **--------------------------------------------------------------------------------------------------------------------------------------------------------------------------------------------------------**

**RN0AAA434YP23FM1**  **---------------------------------------------------------------------------------------------------------------------------------------------------------------------------------AT.TTTCATGTAATTAAAAAT.T**

**RN0AAA462YN08FM1**  **--------------------------------------------------------------------------------------------------------------------------------------------------------------------------------------------AATTAAAA.TGT**

**RN0AAA474YG15FM1**  **-------------------------------------------------------------------------------------------------------------------------------------ACATCTATATCTAATTTAAATTCGTGAATTAT-AAATTATACAAAA.TATCATGTAATTAAAAATGT**

**RN0AAA481YC13FM1**  **-----------------------------------------------------------------------------------------------------------------------------------------------------------CGAGAATTAT-AAATTAAACTAAT.TTTCATGTAGTTAAAAAT.T**

**RN0AAA556YI14FM1**  **---------------------------------------------------------------------------------------------------------------------------------------------------------------------------------------------------AATGT**

**RN0AAA76YA17RM1**  **--------------------------------------------------------------------------------------------------------------------------------------------------------------------------------------------------------**

**RN0AAA94YM05RM1**  **----------------------------------------------------------------------------------------------------------------------------------------------------------------------------------------------------AT.T**

**RN0AAB55YM18FM1**  **-------------------------------------------------------------------------------------------------ATACAGTTTTTAACGCTCTTTCGAATGATATATAGAACTTCTATCTCAAATTTAAATTCGTGAATTAT-AAATTATACAAAT.TTTCATGTAATTAAAAAT.T**

**RN0AAB56YP24FM1**  **--------------------------------------------------------------------------------------------------------------------------------------------------------------------------------------------------------**

**RN0AAA513YA20RM1**  **--------------------------------------------------------------------------------------------------------------------------------------------------------------------------------------------------------**

**Box 1**  **--------------------------------------------------------------------------------------------------------------------------------------------------------------------------------------------------------**

210 220 230 240 250 260 270 280 290 300 310 320 330 340 350 360 370 380 390 400

....|....|....|....|....|....|....|....|....|....|....|....|....|....|....|....|....|....|....|....|....|....|....|....|....|....|....|....|....|....|....|....|....|....|....|....|....|....|....|....|

**RN0AAB125YE10FM1**  **CCCCC---TTTTTTTCCCTATTCC-CCTGACCATTTTGAGCTTT----TTTGCTT-GACATA----TAATTT--ACAATATATCATTC-GAAAGAGCTGTTCGAG-CTG--AGTAG-AATGAT--ACTATGATCGGTTAGATTTGGCGAATCTGAACAG--ATTTATGAAGCTAAATCCTCGATGGACGTGCTTTT-TGT**

**RN0AAB3YE08AHM1**  **.....CCC................-...................----.......-......----......--..............-................-...--.....-......--......T...........................--...................................-...**

**RN0AAB140YN05FM1**  **.....---C...............-...................----.......-......----......--..............-................-...--.....-......--..................................--...................................-...**

**RN0AAB414YN09FM1**  **.....C--A...............-.........C.........----.......-......----......--..............-................-...--.....-......--..................................--...........C.......................-...**

**RN0AAB545YM04AHM1** **.....C--A...............-.........C.........----.......-......----......--..............-................-...--.....-......--..................................--...........C.......................-...**

**RN0AAA130YM16FM1**  **.....C--A...............-.........C.........----.......-......----......--..............-................-...--.....-......--..................................--...........C.......................-...**

**RN0AAA519YK05RM1**  **-----------------------------------------------------------...----......--..............-................-...--.....-......--..................................--...........C.......................-...**

**RN0AAB125YE10FM1**  **---------...............-...................----.......-......----......--..............-................-...--.....-......--..................................--...................................-...**

**RN0AAB3YE08AHM1**  **---------...............-...................----.......-......----......--..............-................-...--.....-......--......T...........................--...................................-...**

**RN0AAB140YN05FM1**  **---------...............-...................----.......-......----......--..............-................-...--.....-......--..................................--...................................-...**

**RN0AAB414YN09FM1**  **---------...............-.........C.........----.......-......----......--..............-................-...--.....-......--..................................--...........C.......................-...**

**RN0AAB545YM04AHM1** **---------...............-.........C.........----.......-......----......--..............-................-...--.....-......--..................................--...........C.......................-...**

**RN0AAA130YM16FM1**  **---------...............-.........C.........----.......-......----......--..............-................-...--.....-......--..................................--...........C.......................-...**

**RN0AAA519YK05RM1**  **-----------------------------------------------------------...----......--..............-................-...--.....-......--..................................--...........C.......................-...**

**RN0AAB176YD12FM1**  **---------CCCCCC...CT....-...................----.......-......----....G.--..............-................-...--.....-......--.......A................T........A--.A...C.....-..........G...--.A.....-...**

**RN0AAA286YA16FM1**  **---------.......----..T.-......T............----...A...-...T..----......--..............-.........A...T..-...--.....-......--.............C..C....A..TA....G...--.A.........................A.......-...**

**RN0AAA179YC21RM1**  **----------C....TTA.G..T.-...A...............----.......-......CCTA......--.TT...........-.........A.C....-.C.--.....-.T....--....A.....T.............T.T......A--.A......G...........TG.....A.......-...**

**RN0AAA756YE07FM1**  **----------C....TTA.G..T.-...A...............----.......-......CCTA......--.TT...........-.........A.C....-.C.--.....-.T....--....A.....T.............T.T......A--.A......G...........TG.....A.......-...**

**RN0AAA591YF08RM1**  **------------------------------------------------------------A.----...A..--.TC......A...T-.....CT..C.A....-..C--G..C.-......--..C.....T..G.....C..ATC.T...T.C..A--.A.................ATT...GTAAA.....-...**

**RN0AAA538YB16RM1**  **-------------------G..T.-T................-.-T--...-G..T..A.C.----......T-.TC...........-.....CT..C.A....-..C--G..C.-......--..C.....T..C.....C..ATC.T...T.C..A--.A.................ATT...GTAAA.....--..**

**RN0AAB458YP20AHM1** **---------....CA--.CG..T.-.................-.-T--...-G..T..A.C.----......--.TC...........-.....CT..C.A....-..C--G..C.-......--..C..A..T..C.....C..ATC.T...T.C..A--.A.................ATT...GT.AA.....-...**

**RN0AAA669YI24RM1**  **---------.......A.CG..T.-.................-.-T--...-G..T..T.C.----......--.TC...........-.....CT..C......-..T--G..C.-......--..C.....T..C.....C..ATC.T...T.C..A--.A.................T.TC..G.AAA.....-...**

**RN0AAA87YA19FM1**  **---------...CCC--.C...T.-T...........T....A.-T--...-G..T..T...----......--.TT..........--.G..T....C..T...-..T--G..C.-......--..C.-...TA.C.....C..TT..T...T.T..A--.A....A..............TA..GAAAA.....-...**

**RN0AAB380YP14FM1**  **---------...CAC---CG..T.-.................-.-T--...-G..T..T...----......--.TC........GA.-.........C......-..T--T..C.-......--..C.AA..T..C...C.C..AT.CT...T.CA.A--.A....A..A...........T...GAAATT....-...**

**RN0AAB423YM10FM1**  **---------...CAC---CG..T.-.................-.-T--...-G..T..T...----......--.TC........GA.-.........C......-..T--T..C.-......--..C.AA..T..C...C.C..AT.CT...T.CA.A--.A....A..A...........T...GAAATT....-...**

**RN0AAB147YJ10FM1**  **---------CCCCC.---CC.AT.--....T....G........-T--...-GC.T..T...----..-------T............-.......T.TA.....-...--T..TA-...T-.--........T.....A.....ATCGT.T.A....A--.A.........C.....AA..C....AAAA.C...-...**

**RN0AAA64YH05FM1**  **---------....CC---CG..T.-A..........G.......-T--...-A..T..T...----......--TTT...........-.........C.....T-.CT--G..C.-......--.TC.A...T..C........AT..T...T.CT.A--.A...........G....A..T....AAA......-...**

**RN0AAB176YD12FM1**  **ATAGTT--CCCCCCC...CT....-...................----.......-......----....G.--..............-................-...--.....-......--.......A................T........A--.A...C.....-..........G...--.A.....-...**

**RN0AAA286YA16FM1**  **....T----.......----..T.-......T............----...A...-...T..----......--..............-.........A...T..-...--.....-......--.............C..C....A..TA....G...--.A.........................A.......-...**

**RN0AAA179YC21RM1**  **G....C----C....TTA.G..T.-...A...............----.......-......CCTA......--.TT...........-.........A.C....-.C.--.....-.T....--....A.....T.............T.T......A--.A......G...........TG.....A.......-...**

**RN0AAA756YE07FM1**  **G....C----C....TTA.G..T.-...A...............----.......-......CCTA......--.TT...........-.........A.C....-.C.--.....-.T....--....A.....T.............T.T......A--.A......G...........TG.....A.......-...**

**RN0AAA591YF08RM1**  **-----------------------------------------------------------.C.----...A..--.TC......A...T-.....CT..C.A....-..C--G..C.-......--..C.....T..G.....C..ATC.T...T.C..A--.A.................ATT...GTAAA.....-...**

**RN0AAA538YB16RM1**  **-------------------G..T.-T................-.-T--...-G..T..A.C.----......--.TC...........-.....CT..C.A....-..C--G..C.-......--..C.....T..C.....C..ATC.T...T.C..A--.A.................ATT...GTAAA.....--..**

**RN0AAB458YP20AHM1** **.....CCT.....CA--.CG..T.-.................-.-T--...-G..T..A.C.----......--.TC...........-.....CT..C.A....-..C--G..C.-......--..C..A..T..C.....C..ATC.T...T.C..A--.A.................ATT...GT.AA.....-...**

**RN0AAA669YI24RM1**  **.....CTT........A.CG..T.-.................-.-T--...-G..T..T.C.----......--.TC...........-.....CT..C......-..T--G..C.-......--..C.....T..C.....C..ATC.T...T.C..A--.A.................T.TC..G.AAA.....-...**

**RN0AAA87YA19FM1**  **.....CTT....CCC--.C...T.-T...........T....A.-T--...-G..T..T...----......--.TT..........--.G..T....C..T...-..T--G..C.-......--..C.-...TA.C.....C..TT..T...T.T..A--.A....A..............TA..GAAAA.....-...**

**RN0AAB380YP14FM1**  **.....CTT....CAC---CG..T.-.................-.-T--...-G..T..T...----......--.TC........GA.-.........C......-..T--T..C.-......--..C.AA..T..C...C.C..AT.CT...T.CA.A--.A....A..A...........T...GAAATT....-...**

**RN0AAB423YM10FM1**  **.....CTT....CAC---CG..T.-.................-.-T--...-G..T..T...----......--.TC........GA.-.........C......-..T--T..C.-......--..C.AA..T..C...C.C..AT.CT...T.CA.A--.A....A..A...........T...GAAATT....-...**

**RN0AAB147YJ10FM1**  **AT...CCTCCCCCC.---CC.AT.--....T....G........-T--...-GC.T..T...----..-------T............-.......T.TA.....-...--T..TA-...T-.--........T.....A.....ATCGT.T.A....A--.A.........C.....AA..C....AAAA.C...-...**

**RN0AAA64YH05FM1**  **AT...CCT.....CC---CG..T.-A..........G.......-T--...-A..T..T...----......--TTT...........-.........C.....T-.CT--G..C.-......--.TC.A...T..C........AT..T...T.CT.A--.A...........G....A..T....AAA......-...**

**RN0AAB311YB19AHM1** **-----------------------------------------------------------------------------------------------------------------------...C--GT..AA...A-ACGA.A-.CT-..C.TAA.TG.ACA.AG.TG-----.G.CCAT.TAT.TT.TCC-AC..GCATA**

**RN0AAB44YH23AHM1**  **-------------------------------------------------------------------------------------------------------------------------------------------------------------.ACATAG.TA-----.G.GCAT.T.T.TT.ATC-.C...CA.C**

**RN0AAA717YI15RM1**  **.....CCC.....-----CC..T.TAT.----T....CGAT...---T.A---AACATTT..TGG-C..A..-----...........-.........C......-...--...C.-..C.G.--....GAT..A-.CGA.A-.CC-..C.TAA.TA.ACATAG.TA-----.G.GCAT.T.T.TT.A-C-.CC..CA.C**

**RN0AAB125YA16FM1**  **.....CCT......----CC..T.TAT.---TT..C.T..T...---T.C-T-C--ATTT..TGA-C..A..-----...........-.........C......-...--...C.-.....C--.T..AA...A-ACGAGA-.CT-..C.TAAGTG.ACA.AG.-----TG.G.CCG..TAT.TT.TCC-AC..GCAT.**

**RN0AAB437YE21AHM1** **.....CCT......----CC..T.TAT.---TT..C.T..T...---T.C-T-C--ATTT..TGA-C..A..-----...........-.........C......-...--...C.-.....C--.T..AA...A-ACGAGA-.CT-..C.TAAGTG.ACA.AG.-----TG.G.CCG..TAT.TT.TCC-AC..GCAT.**

**RN0AAB266YH16AHM1** **.....C--......----CC..T.TAT.---TT..C.T..T...---T.C-T-C--ATTT..TGG-C..A..-----...........-.........C......-...--...T.-.....C--.T..AA...A-ACGAGA-.CT-..C.TAAGTG.ACA.AG.-----TG.G.CCGT..AT.TT.TCC-AC..GCAT.**

**RN0AAA131YF02RM1**  **.....CCT......----CC..T.TAT.---TT..C.T..T...---T.C-T-C--ATTT..TGG-C..A..-----...........-.........C......-...--...C.-.....C--.T..AA...A-ACGAGA-.CT-..C.TAAGTG.ACA.AG.-----TG.G.CCA.CTAT.TT.TCC-AC..GCAT.**

**RN0AAA130YE05RM1**  **.....CCT......----CC..T.TAT.---TT..C.T..T...---T.C-T-C--ATTT..TGG-C..A..-----...........-.........C......-...--...C.-.....C--.T..AA...A-ACGAGA-.CT-..C.TAAGTG.ACA.AG.-----TG.G.CCA.CTAT.TT.TCC-AC..GCAT.**

**RN0AAA579YK24RM1**  **-.GTTTTTCCC---------C.TTAAT.----T..C---A....---T..---.-CTTTT..TGT-C..A..-----...A.......-....A.A..C......-...--...T.G.....C----..A....A-A.GA.A-.CT-..C.TAA.T..ACATAG..GT--TA.G.TCTT.T...TT.ATA-AC..GCA.C**

**RN0AAB100YP08AHM1** **T....CCCCCC...----.CC.T-TAT.---TT..CCT..T...---T.C-T-C--.TTT..TGG-C..A..-----...........-.........C...A..-...--...C.-..A...--....AA...A-ACGA.A-.CT-..T.TAATTG.ACA.AG.-----TG.G.CCGT.T.T.TT.TCC-AC..GAATC**

**RN0AAB180YL08FM1**  **T....CCCCCC...----.CC.T-TAT.---TT..CCT..T...---T.C-T-C--.TTT..TGG-C..A..-----...........-.........C...A..-...--...C.-..A...--....AA...A-ACGA.A-.CT-..T.TAATTG.ACA.AG.-----TG.G.CCGT.T.T.TT.TCC-AC..GAATC**

**RN0AAA175YL12RM1**  **.....CCT......----CC.CT.TAT.---TT..C.T..T...---T..-TTC--ATTT..TGG-C..A..-----...........-.........C......-...--...C.-.....G--.T..AA...A-A.GA.A-.CT-..CATAACTG.ACA.AG.-----TG.G.CCA.CTAT.TT.TTC-AC..CCAT.**

**RN0AAB484YE18FM1**  **.....CCT.....-----CC..T.TAT.---TT..CCT..T..C---T.G-T-C--ATTT..TGA-C..A..-----...........-.........C...A..-...--...C.-......--.T..AA...A-ACGA.C-.CT-..C.TATGTG.ACA.AG.-----TG.G.CCGT.TAT.TT.TCC-AC..ACAT.**

**RN0AAA316YE18RM1**  **.....CTT...C.-----CC.CT.TAT.---TT..C.T..T...---T.C-T-C--ATTT..TGG-C..A..-----...........-.........C......-...--...C.-.....C--.T..AA...A-ACGA.A-.CT-..C.TAAGTG.ACA.AG.-----TG.G.CCGT.TAT.TT.TCC-AC..GCAT.**

**RN0AAA14YA24RM1**  **.....CTT...C.-----CC.CT.TAT.---TT..C.T..T...---T.C-T-C--ATTT..TGG-C..A..-----...........-.........C......-...--...C.-.....C--.T..AA...A-ACGA.A-.CT-..C.TAAGTG.ACA.AG.-----TG.G.CCGT.TAT.TT.TCC-AC..GCAT.**

**RN0AAA691YK01RM1**  **.AT..CCC..C...----CC..T.T-T.---TT....C.AT...---T.C-CAC--ATTT..TGG-C..A..-----...........-.........T...A..-.A.--...C.-GG..G.--....A.....-ACGA.A-.TT-..C.TAA.TG.ACA.AG.-----TA.G.CCAT.A.T.TT.----AAA.GC.T.**

**RN0AAB526YI05AHM1** **.AT..CCC..C...----CC..T.T-T.---TT....C.AT...---T.C-CAC--ATTT..TGG-C..A..-----...........-.........T...A..-.A.--...C.-GG..G.--....A.....-ACGA.A-.TT-..C.TAA.TG.ACA.AG.-----TA.G.CCAT.A.T.TT.----AAA.GC.T.**

**RN0AAB490YN21AHM1** **G....TCT......---TATG.T.TAT-----T....TCAT..C---C.A..-AAATTTC..TGG-ATT...-----.....-.....-.........C......-...--...C.-..C.G.--....AAT..A-.C.A.A-.CC-..C.TAA.TA.ACA.AG.TA-----.G.GCAT.T.T.TT.ATC-.C...CAAC**

**RN0AAA132YI06RM1**  **G....CCT....-----TATG.T.TAT-----T....CCAT..C---C.A..-AAATTTC..TGA-ATT...-----.....-.....-.........CC.....-...--...CA-.TC.GC--....GA...A-.CGA.A-.CT-..C.TAA.T..ACATAG.TA-----.G.GCAT.T.T.TT.ATC-AC...CAAC**

**RN0AAB289YD03FM1**  **.....TAT...CC.-------.TTTAT.T---T....CGAT...---T.A---AAATTTT..TGG-C..A..-----...........-.........C......-...--...C.-....G.--....GAT..A-.CGA.AA.CC-..C.TAA.TA.ACATAG.TA.G-TA.G.GCGT.T.T.TT.ATC-.C...CA.C**

**RN0AAA727YI23RM1**  **.....CTT....CC----C...T.TAT.----T....CG.G...---T.A---AACTTTT..TGG-C..A..-----...........-.........C......-...--...C.-..C.C.--....AAT..A-.CGA.A-.CC-..T.TAA.TA.AGATAG.TA-----.G.GCAT.T.T.TT.ATC-.....CAAC**

**RN0AAA90YK10FM1**  **.....CCT.....-----CC..T.TAT.----T....CGAT...---T.A---AACATTT..TGG-C..A..-----...........-.........C......-...--...C.-..C.G.--....GAT..A-.CGA.A-.CC-..C.TAA.TA.ACATAG.TA-----.G.GCAT.T.T.TT.ATC-.C...CA.C**

**RN0AAB20YB17AHM1**  **.....CCT....------CC..T.TAT.----T....CGAT...---T.A---AACATTT..TGG-C..A..-----...........-.........C......-...--...C.-..C.G.--....GAT..A-.CGA.A-.CC-..C.TAA.TA.ACATAG.TA-----.G.GCAT.T.T.TT.ATC-.C...CA.C**

**RN0AAB577YG05AHM1** **.....CCT....------CC..T.TAT.----T....CGAT...---T.A---AACATTT..TGG-C..A..-----...A.......-.........C......-...--...C.-..C.G.--....GAT..A-.CGA.A-.CC-..C.TAA.TA.ACATAG.TA-----.G.GCAT.T.T.TT.ATC-.C...CA.C**

**RN0AAB48YA06AHM1**  **---------------------------------------------------------------GG-C..A..-----...A.......-.........C......-...--...C.-..C.G.--....GAT..A-.CGA.A-.CC-..C.TAA.TA.ACATAG.TA-----.G.GCAT.T.T.TT.ATCA.C...CA.C**

**RN0AAA133YD03FM1**  **---------------------------------------------------------------------------------------------------......-...--...C.-..C.G.--....GAT..A-.CGA.A-.CC-..C.TAA.TA.ACATAG.TA-----.G.GCAT.T.T.TT.ATC-.C...CA.C**

**RN0AAA542YE07RM1**  **.....CCCC.....----CC..T.TAT.----T....CGAT...---T.A---AACATTT..TGG-C..A..-----...........-.........C......-...--...C.-..C.G.--....GAT..A-.CGA.A-.CC-..C.TAA.TA.ACATAG.TA-----.G.GCAT.T.T.TT.ATC-.C...CA.C**

**RN0AAA601YB08RM1**  **.....CCCC.....----CC..T.TAT.----T....CGAT...---T.A---AACATTT..TGG-C..A..-----...........-.........C......-...--...C.-..C.G.--....GAT..A-.CGA.A-.CC-..C.TAA.TA.ACATAG.TA-----.G.GCAT.T.T.TT.ATC-.C...CA.C**

**RN0AAA281YH13FM1**  **.....CCCC.....----CC..T.TAT.----T....CGAT...---T.A---AACATTT..TGG-C..A..-----...........-.........C......-...--...C.-..C.G.--....GAT..A-.CGA.A-.CC-..C.TAA.TA.ACATAG.TA-----.G.GCAT.T.T.TT.ATC-.C...CA.C**

**RN0AAA699YB04FM1**  **G....CCC......---TATG.T.TAT-----T....TCAT..C---C.A..-AAATTTC..TGGA.TT...-----.....-.....-.........C......-...--...C.-..C.GC--....GA...A-..GA.A-.CT-..C.TAA.T..ACATAG.TA-----.G.GCAT.T.T.TT.ATC-.C...CAAC**

**RN0AAA437YA19RM1**  **AAGTAGCC.C.---------C.TTTTTC----T..C.--AT...---TC.-----GTTTC..TTG-C..GC.-----...........-.........C.A....-...--.A.C.-....GA--....AA...A-A.GA.A-.CT-..C.TAA.TG..CA.AG.TA-----.G.GCGT.T.T.CT.ATC-.C..GAAC.**

**RN0AAA499YO02FM1**  **AAGTAGCC.C.---------C.TTTTTC----T..C.--AT...---TC.-----GTTTC..TTG-C..GC.-----...........-.........C.A....-...--.A.C.-....GA--....AA...A-A.GA.A-.CT-..C.TAA.TG..CA.AG.TA-----.G.GCGT.T.T.CT.ATC-.C..GAAC.**

**RN0AAA377YF05FM1**  **AAGTAGCC.C.---------C.TTTTTC----T..C.--AT...---TC.-----GTTTC..TTG-C..GC.-----...........-.........C.A....-...--.A.C.-....GA--....AA...A-A.GA.A-.CT-..C.TAA.TG..CA.AG.TA-----.G.GCGT.T.T.CT.ATC-.C..GAAC.**

**RN0AAA222YD17RM1**  **--------------------------------------------------------------CCT--GGC..T-TTTTG...CAG..TTT..C.CT..T.-...A--..AT.TATA--GAAC.----.C.----ATC.CA.A-------T.TAA.TA-CGCGAA.TAT.------.ATTATACAA---A.-A...CA...**

**RN0AAB173YC20AHM1** **..AA.TTACG..AA.T...GG.GTA.A.T-.A.C..GA-AAA..AAAT.GAAAAACA.-T..CCT--GGC..T-TTTTG...CAG..TTT..C.CT..T.-...A--..AT.TATA--GAAC.----.C.----ATC.CA.A-------T.TAA.TT-CGCGAA.TAT.------.ATTATACAA---AA-A...CA...**

**RN0AAB161YD24FM1**  **------------------GCG.GTA.A.T-.A.C..GT-AAA..AAAC.GAAAAACA.-T..CCT--GGC..T-TTTTG...CAG..TTT.TC.CT..T.-...A--..AT.TATA--GAAC.----.T.----ATC.CA.A-------T.TAA.TT-CGTGAA.TAT.------.ATTATACAA---A.-A...CA...**

**RN0AAB55YM18AHM1**  **---------------T...GG.GTA.A.T-.A.C..GA-.AA..AAAT.GAAAAACA.-T..CCT--GGC..T-TTTTG...CAG..TTT..C.CT..T.-...A--..AT.TATA--GAAC.----.C.----ATC.CA.A-------T.TAA.TT-CGTGAA.TAT.------.ATTATACAA---A.-A...CA...**

**RN0AAA481YC13RM1**  **..AA.TTACG..AA.T.G.TG.GTA.A.T-.A.C..GA-AAA..AAAT.GAAAAACA.-T..CCT--GGC..T-CTTTG...CAG..TTT..C.CT..T.-...A--..AT.TATA--GAAC.----.G.----ATC.CA.A-------T.TAA.TA-CGCGAA.TAT.------.ATTATACAA---A.-A...CA...**

**RN0AAA81YK04RM1**  **-----------------------TA.A.T-.A.C..GA-AAA..AAAT.GAAAAACA.-T..CCT--GGC..T-TTTTG...CAG..TTT..C.CT..T.-...A--..AT.TATA--GAAC.----.C.----ATC.CA.A-------T.TAA.TT-CGCGAA.TAT.------.ATTATACAA---A.-A...CA...**

**RN0AAA229YJ17FM1**  **----------------------------------------------------.AATT.-C.CTGG--CT...T-TT--G...CAG..TTT..C.CT..T.-...A--..AT.TATA--GAAC.----.C.----ATC.CA.--A----TT.-AA.TA-CGTGAA.TAT.------.ATTATACA----AATA...CA...**

**RN0AAA222YD17FM1**  **..AA.TTACG..AA.T...TG.GTA.A.T-.A.C..GA-AAA..AAAT.GAAAAACA.-T..CCT--GGC..T-TTTTG...CAG..TTT..C.CT..T.-...A--..AT.TATA--GAAC.----.C.----ATC.CA.--A-----T.TAA.T-.CGCGAA.TAT.------.ATTATACA----AATA...CA...**

**RN0AAA303YG05RM1**  **..AA.TTACG..AA.T...GG.GTA.A.T-.A.C..GA-AAA..AAAT.GAAAAACA.-T..CCT--GGC..T-TTTTG...CAG..TTT..C.CT..T.-...A--..AT.TATA--GAAC.----.C.----ATC.CA.A.-------.TAA.TT-CGCGAA.TAT.------.ATTATACAA---A.-A...CA...**

**RN0AAA32YB09FM1**  **-----------------------------------------------------------------------------------------------------------------ATA--GAAC.----.C.----ATC.CA.--A----TT.-AA.TT-CGTGAA.TAT.------.ATTATACA----AATA...CA...**

**RN0AAA474YI20RM1**  **..AA.TTACG..AA.T...GG.GTA.A.T-.A.C..GA-AAA..AAAT.GAAAAACA.-T..CCT--GGC..T-TTTTG...CAG..TTT..C.CT..T.-...A--..AT.TATA--GAAC.----.C.----ATC.CA.--A-----T.TAA.T-TCGTGAA.TAT.------.ATTATACA----AATA...CA...**

**RN0AAA373YI20FM1**  **--------------------------------------------------------------------------------------------------------------------------------------------------------------------------------------------------------**

**RN0AAA434YP23FM1**  **..AA.TTACG..AA.T...GG.GTA.A.T-.A.C..GA-AAA..AAAT.GAAAAACA.-T..CCT--GGC..T-TTTTG...CAG..TTT..C.CT..T.-...A--..AT.TATA--GAAC.----.C.----ATC.CA.--A----TT.-AA.TA-CGCGAA.TAT.------.ATTATACA----AATA...CA...**

**RN0AAA462YN08FM1**  **..AA.TTACA..AA.T...GGCG.A.A.T-.A.C..GAGAAA..AAAT.GAAAAACA.-T..CCT--GGCG.T-TTTTG...CAG..TTT..C.CT..T.-...A--..AT.TATA--GAAC.----.C.----ATC.CA.--A----TT.-AA.T.TCGCGAA.TAT.------.ATTATACA----AATA...CA...**

**RN0AAA474YG15FM1**  **..AA.TTACG..AA.T...TG.GTA.A.T-.A.G..GA-AAA..AAAT.GAAAAACA.-T..CCT--GGC..T-TTTTG...CAG..TTT..C.CT..T.-...A--..AT.TATA--GAAC.----.C.----ATC.CA.A.-------.TAA.TT-CGCGAA.TAT.------.ATTATACAA---AA-A...CA...**

**RN0AAA481YC13FM1**  **G.AA.TTACG..AA.T.G.GG.GTA.A.T-.A.C..GA-AAA..AAAT.GAAAAACA.-T..CCT--GGC..T-TTTTG...CAG..TTT..C.CT..T.-...A--..AT.TATA--GAAC.----.C.----ATC.CA.--A----TT.-AA.TTGCGCGAA.TAT.------.ATTATACA----AAAA...CA...**

**RN0AAA556YI14FM1**  **A.TT.GTAC.A.AA.G.G.CG.GTA.A.T-TT.C..G.CCG.C.T--T.AAAAAACA.-T..CCT--GGC..T-TTTTG...CAG..TTT..C.CT..T.-...A--..AT.TATA--GAAC.----.C.----ATC.CA.--A----TT.-AA.TT-CGCGAA.TAT.------.ATTATACA----AAAA...CA...**

**RN0AAA76YA17RM1**  **----------------------------------------------------------------------------------------------CT..T.-...A--..AT.TATA--GAAC.----.C.----ATC.CA.A.-------.GAA.TT-CGCGA..TAT.------.ATTATACAA---A.-A...CA...**

**RN0AAA94YM05RM1**  **..AA.TTACG..AA.T...GG.GTA.A.T-.A.C..GA-TAA..AAAT.GAAAAACA.-T..CCT--GGC..T-TTTTG...CAG..TTT..C.CT..T.-...A--..AT.TATA--GAAC.----.C.----ATC.CA.--A----TT.-AA.TT-CGTGAA.TAT.------.ATTATACA----AATA...CA...**

**RN0AAB55YM18FM1**  **..AA.TTACG..AA.T...GG.GTA.A.T-.A.C..GA-AAA..AAAT.GAAAAACA.-T..CCT--GGC..T-TTTTG...CAG..TTT..C.CT..T.-...A--..AT.TATA--GAAC.----.C.----ATC.CA.--A----TT.-AA.TT-CGCGAA.TAT.------.ATTATACA----AATA...CA...**

**RN0AAB56YP24FM1**  **---------------------------------------------------------.-CCCTGG--GT...T-TTTGG...CAG..TTT..C.CT..T...TCA--..AT.TATA--GAAC.----.C.----ATC.CA.--A----TT.TAA.TT-CGCGAA.TAT.------.ATTATACA----AATA...CA...**

**RN0AAA513YA20RM1**  **--------------------------------------------------------------------------------------------------------------------------------------------------------------------------------------------------------**

**Box 1**  **--------------------------------------------------------------------------------------------------------------------------------------------------------------------------------------------------------**

410 420 430 440 450 460 470 480 490 500 510 520 530 540 550 560 570 580 590 600

....|....|....|....|....|....|....|....|....|....|....|....|....|....|....|....|....|....|....|....|....|....|....|....|....|....|....|....|....|....|....|....|....|....|....|....|....|....|....|....|

**RN0AAB125YE10FM1**  **GATGCTGAAAG----GGATTAAAACA--AATGCTC-ATAACTA----TGTTTATTT-----AAGT--CGGATTTC--GATGAAT--CTA---G---TACCGT--T----CGACTCAGC-------TCGAAGAGCTCTTTCGAATGATATATAATTTGCCAT--AGAATGTTTTAA-------AAATCGAAAAAA-ATAGA**

**RN0AAB3YE08AHM1**  **...........----...........--.......-.......----.........-----....--........--.......--...---.---......--.----.........-------....................................--.A.......A..-------............-.....**

**RN0AAB140YN05FM1**  **...........----...........--.......-.......----.........-----....--........--.......--...---.---......--.----.........-------....................................--.A.T.....A..-------............-.....**

**RN0AAB414YN09FM1**  **...........----...........--.......-.......----.........-----....--........--.......--...---.---......--.----.........-------......................T.............--.A.......A..-------............-.....**

**RN0AAB545YM04AHM1** **...........----...........--.......-.......----.........-----....--........--.......--...---.---......--.----.........-------......................T.............--.A.......A..-------............-.....**

**RN0AAA130YM16FM1**  **...........----...........--.......-.......----.........-----....--........--.......--...---.---......--.----.........-------......................T.............--.A.......A..-------............-.....**

**RN0AAA519YK05RM1**  **...........----...........--.......-.......----.........-----....--........--.......--...---.---......--.----.........-------......................T.............--.A.......A..-------............-.....**

**RN0AAB125YE10FM1**  **...........----...........--.......-.......----.........-----....--........--.......--...---.---......--.----.........-------....................................--............-------............-.....**

**RN0AAB3YE08AHM1**  **...........----...........--.......-.......----.........-----....--........--.......--...---.---......--.----.........-------....................................--.A.......A..-------............-.....**

**RN0AAB140YN05FM1**  **...........----...........--.......-.......----.........-----....--........--.......--...---.---......--.----.........-------....................................--.A.T.....A..-------............-.....**

**RN0AAB414YN09FM1**  **...........----...........--.......-.......----.........-----....--........--.......--...---.---......--.----.........-------......................T.............--.A.......A..-------............-.....**

**RN0AAB545YM04AHM1** **...........----...........--.......-.......----.........-----....--........--.......--...---.---......--.----.........-------......................T.............--.A.......A..-------............-.....**

**RN0AAA130YM16FM1**  **...........----...........--.......-.......----.........-----....--........--.......--...---.---......--.----.........-------......................T.............--.A.......A..-------............-.....**

**RN0AAA519YK05RM1**  **...........----...........--.......-.......----.........-----....--........--.......--...---.---......--.----.........-------......................T.............--.A.......A..-------............-.....**

**RN0AAB176YD12FM1**  **....T......----...C.......--.......-.....------.........-----....--........--.......--T..---.---....A.--.----.........-------..................................----.A..A....A..-------............--....**

**RN0AAA286YA16FM1**  **...AT......----...........--....G..-.......----.........-----....--.......T--.......--T..---.---.....G--.----.....T...-------.T................C.................--.A..A.A..A..-------.........C..-..G..**

**RN0AAA179YC21RM1**  **....T......----...........--.......-.....------.........-----....--.......T--.......--T..---.---.....A--.----.........-------....................TA...A..A.--....--....AT..CAT.GG-----....GA......T.....**

**RN0AAA756YE07FM1**  **....T......----...........--.......-.....------.........-----....--.......T--.......--T..---.---.....A--.----.........-------....................TA...A..A.--....--....AT..CAT.GG-----....GA......A.....**

**RN0AAA591YF08RM1**  **.CCAA..G..----GT..A.....A.--GG..GG.-.C....T-T---...C.C..-----.T..--.A..A...--AT...T.--T..---A---.CT.A.--.----.........-------....................................--.A....AAAA..-------...CTA.G....A.....**

**RN0AAA538YB16RM1**  **.CCAA..C..----GT.GA.....T.--GG..G..-.C....T-T---.....C..-----....--.A...C..--.T...T.--T..---A---.GT.A.--.----.........-------....................................--.A....AGAA..-------.T-CTA.G....A.....**

**RN0AAB458YP20AHM1** **.CCAA..C..----GT.GA.....T.--GG..G..-.C....T-T---...C.C..-----....--.A...C..--.T...T.--T..---A---.GT.A.--.----.........-------....................................--.A....AGAA..-------..-CTA.G....A.....**

**RN0AAA669YI24RM1**  **.CCAA..C..----GT.GA.....TG--..C.G..-.C....T-T---...C.C..-----....--.A......--.T...T.--T..---A---.GT.A.--.----.........-------.T..................................--.A....AGAAGT-------..-CTAGG....A.....**

**RN0AAA87YA19FM1**  **.CCAA..C..----G...A.....T.--..C.G..-.C.G..T-TTT-...CGC..-----....T-.AAT....--.T...T.--T..---A---.GT.A.--.----.......A.-------...C.A..................--........-.--.A....AGAA.T-------.--CTA--....A.....**

**RN0AAB380YP14FM1**  **.CA.A.TC..----GT.GA.......--..C.GG.-.C....T-T---...C.A..-----..A.--.A......--.T...T.--T..---.---..T.T.--.----.........-------.T..................................--.A..C.AGAA..-------..-CTAGG....A...--**

**RN0AAB423YM10FM1**  **.CA.A.TC..----GT.GA.......--..C.G..-.C....T-T---...C.A..-----..A.--.A......--.T...T.--T..---.---..T.T.--.----.........-------.T..................................--.A..C.AGAA..-------..-CTAGG....A...--**

**RN0AAB147YJ10FM1**  **CCCA...C..A---G...........--..C.G..-......T-T---T..G.--------...A--.A.....T--AT.C.T.--T..---.---..T.A.--.----...T..------------.....A..T....A......T.....A..TT...---A....AGAA..AAA----..TC.A......AGGG.G**

**RN0AAA64YH05FM1**  **ACC....C..----GT..........--.C..G..-.C....T-A---........-----....--.A......--AT....C--T..---.---....AA--C----.A...T...-------.T..........................A.....G.--.T.....GAA..------A.TTGAA......A.....**

**RN0AAB176YD12FM1**  **....T......----...C.......--.......-.....------.........-----....--........--.......--T..---.---....A.--.----.........-------..................................----.A..A....A..-------............--....**

**RN0AAA286YA16FM1**  **...AT......----...........--....G..-.......----.........-----....--.......T--.......--T..---.---.....G--.----.....T...-------.T................C.................--.A..A.A..A..-------.........C..-..G..**

**RN0AAA179YC21RM1**  **....T......----...........--.......-.....------.........-----....--.......T--.......--T..---.---.....A--.----.........-------....................TA...A..A.--....--....AT..CAT.GG-----....GA......T.....**

**RN0AAA756YE07FM1**  **....T......----...........--.......-.....------.........-----....--.......T--.......--T..---.---.....A--.----.........-------....................TA...A..A.--....--....AT..CAT.GG-----....GA......A.....**

**RN0AAA591YF08RM1**  **.CCAA..G..----GT..A.....A.--GG..GG.-.C....T-T---...C.C..-----.T..--.A..A...--AT...T.--T..---A---.CT.A.--.----.........-------....................................--.A....AAAA..-------...CTA.G....A.....**

**RN0AAA538YB16RM1**  **.CCAA..C..----GT.GA.....T.--GG..G..-.C....T-T---.....C..-----....--.A...C..--.T...T.--T..---A---.GT.A.--.----.........-------....................................--.A....AGAA..-------.T-CTA.G....A.....**

**RN0AAB458YP20AHM1** **.CCAA..C..----GT.GA.....T.--GG..G..-.C....T-T---...C.C..-----....--.A...C..--.T...T.--T..---A---.GT.A.--.----.........-------....................................--.A....AGAA..-------..-CTA.G....A.....**

**RN0AAA669YI24RM1**  **.CCAA..C..----GT.GA.....TG--..C.G..-.C....T-T---...C.C..-----....--.A......--.T...T.--T..---A---.GT.A.--.----.........-------.T..................................--.A....AGAAGT-------..-CTAGG....A.....**

**RN0AAA87YA19FM1**  **.CCAA..C..----G...A.....T.--..C.G..-.C.G..T-TTT-...CGC..-----....T-.AAT....--.T...T.--T..---A---.GT.A.--.----.......A.-------...C.A..................--........-.--.A....AGAA.T-------.--CTA--....A.....**

**RN0AAB380YP14FM1**  **.CA.A.TC..----GT.GA.......--..C.GG.-.C....T-T---...C.A..-----..A.--.A......--.T...T.--T..---.---..T.T.--.----.........-------.T..................................--.A..C.AGAA..-------..-CTAGG....A...--**

**RN0AAB423YM10FM1**  **.CA.A.TC..----GT.GA.......--..C.G..-.C....T-T---...C.A..-----..A.--.A......--.T...T.--T..---.---..T.T.--.----.........-------.T..................................--.A..C.AGAA..-------..-CTAGG....A...--**

**RN0AAB147YJ10FM1**  **CCCA...C..A---G...........--..C.G..-......T-T---T..G.--------...A--.A.....T--AT.C.T.--T..---.---..T.A.--.----...T..------------.....A..T....A......T.....A..TT...---A....AGAA..AAA----..TC.A......AGGG.G**

**RN0AAA64YH05FM1**  **ACC....C..----GT..........--.C..G..-.C....T-A---........-----....--.A......--AT....C--T..---.---....AA--C----.A...T...-------.T..........................A.....G.--.T.....GAA..------A.TTGAA......A.....**

**RN0AAB311YB19AHM1** **.GCA.AA....TCTT---GCC...T---..G.A.--T..G..TC-ATAA...TGGC-----...-----A..G----ATCAG..--...G----CCATT.A.GG.---ATC.T...--ATCAAGC..........................G.-.---A.AT-TATG.A.CAA.CA-----A...AA.CTC...AT---G**

**RN0AAB44YH23AHM1**  **ATCA.AA....-CAC--G.CC.TC----G.G.A.--T..G..TC-ATAAA.CTG..-----C..-----AT.C----.CCA...--...A----CCG.A.A.AG.---ATC.T..T--ACTCAGC.....C....................TG-.---A.AT-TAT....CAAGC------A...AA.CTC...AT---G**

**RN0AAA717YI15RM1**  **ATCA.AA....-CAC--G.CC.TC----G.G.A.--T..G..TC-ATAAA.CTG..-----C..-----AT.C----.CCA...--...A----CCG.T.A.AG.---ATC.T..T--ACTCAGC.....C....................TG-.---A.AT-TAT....CAGGC------A...AA.CTC...A-.--G**

**RN0AAB125YA16FM1**  **.GCA.AA....TTT---.CCC...----T.G.A.--T..G..TC-ACAA...TGG.-----..A-----A..G----ATCAG..--...G----CCA.T.AGGG.---ATC.T..G--ACCGAGC...T....AG................G.-A---A.AT-TA.G.A.CAA.CA-----A...A-.CTC...A-.--G**

**RN0AAB437YE21AHM1** **.GCA.AA....TTT---.CCC...----T.G.A.--T..G..TC-ACAA...TGG.-----..A-----A..G----ATCAG..--...G----CCA.T.AGGG.---ATC.T..G--ACCGAGC...T....AG................G.-A---A.AT-TA.G.A.CAA.CA-----A...A-.CTC...A-.--G**

**RN0AAB266YH16AHM1** **.GCA.AA....TTG---.CGC...----T.G.A.--T..G..TC-ATAA...TGG.-----...-----A..G----ATCAG..--...G----CCA.T.A.GG.---ATT.T...----CGAGC...T....AG................G.-.---A.AT-TC.G.A.CAA.CA-----A...A-.CT....A-.--G**

**RN0AAA131YF02RM1**  **.GCA.AA....TTT---.CCC...----T.G.A.--T..G..TC-ATAA...TGG.-----...-----A..G----ATCAG..--...G----CCA.T.A.GG.---ATC.T..G--ACCGAGC...T....AG................G..A---A.AT-TATG.T.CAA.CA-----A...A-.CTC...A-.--G**

**RN0AAA130YE05RM1**  **.GCA.AA....TTT---.CCC...----T.G.A.--T..G..TC-ATAA...TGG.-----...-----A..G----ATCAG..--...G----CCA.T.A.GG.---ATC.T..G--ACCGAGC...T....AG................G..A---A.AT-TATG.T.CAA.CA-----A...A-.CTC...A..--G**

**RN0AAA579YK24RM1**  **..CA.--------T---T..C.GC----G.G.A.--TC.G..TC-ATAA..ATG..-----G..-----A..C----CTCA...--...G----CCG.A----------------G--ACCCAGC....G.....................-----------------ACCGAGCA-----A...---CTC...A-.--.**

**RN0AAB100YP08AHM1** **TGCA.AA...AATT----.CCC...---G.G.A.--T..GT.TT-ATAA...T.G.-----...-----A.GC----ATCAG.G--...G----CCA.TTT.GG.---ATC.T..G--ACAAAGC................TC........G.-.---A.AT-TAT..A.CAA.CA-----A...A-.CTC...A-.--G**

**RN0AAB180YL08FM1**  **TGCA.AA...AATT----.CCC...---G.G.A.--T..GT.TT-ATAA...T.G.-----...-----A.GC----ATCAG.G--...G----CCA.TTT.GG.---ATC.T..G--ACAAAGC................TC........G.-.---A.AT-TAT..A.CAA.CA-----A...A-.CTC...A-.--G**

**RN0AAA175YL12RM1**  **.GCA.AA....TTT---.CCC...----T.G.A.--T..G..TC-ATAA....GG.-----...-----A..G----A.CAG..--...G----CCA.A.A.GG.---ATC.T...--AC--------------------------------------------------------------------------------**

**RN0AAB484YE18FM1**  **.GCATGA....TTT---.CCC..C----T.G.A.--T..G..TC-CTAA...TG..-----.CA-----AG..----A.CAG..--T..G----CCA.AAA.GA------------------------------------------------------------------------------------------------**

**RN0AAA316YE18RM1**  **.GCA.AA..T.TTTT--.GCC...----..G.GA--T..G..TT-ATAA...TGG.-----.TA-----A..C----ATCAG..--A.----------------------------------------------------------------------------------------------------------------**

**RN0AAA14YA24RM1**  **.GCA.AA..T.TTT--A-GCC...----..G.A.--T..G..TT-ATAA...TGG.-----.TA-----A..C----ATCAG..--A..G----CCA.T.A.GG.---ATC.T..G--TCCAAGC...........C..............G.-.---A.AT-TATG.A.CAA.CA-----A...A-.CTC...A-.--G**

**RN0AAA691YK01RM1**  **------------------.CC..T----G.G.A.--T.TT..TC-ATAA...T.-.-----..A-----A..C----ATCA...--...G----AC..TTT.GG.---ATT.T..G--ACCCAGA..........GGG..........-..C.A.---A.AT-TAT.CAACAA.TA-----A...A-.CTC...A-.--G**

**RN0AAB526YI05AHM1** **------------------.CC..T----G.G.A.--T.TT..TC-ATAA...T.-.-----..A-----A..C----ATCA...--...G----AC..TTT.GG.---ATT.T...--ACCCAGA.....T....GGG........AG-..C.A.---A.AT-TAT.CAACA----------------------------**

**RN0AAB490YN21AHM1** **ATCA.AA....-CAT--G.CC.C.----G.G.A.--T..G.CTC-ATAA...TG..-----C.A-----A..C----.CCA...--...A----CCG.TTT.AG.---ATC.A..T--GCCCAGC...G.T......................-.---A.AT-TAT....CGAGCA-----A...A-.CTC...A-.--G**

**RN0AAA132YI06RM1**  **ATCA.AA...C-CAT--G.CC.TC----G.G.A.--T..G..TC-GTAAG..T...-----C.A-----A..C----.CCA...--...A----CCG.T.A.AG.---ATC.A..T--AACCAGC..AG.T......................-A---A----TA.....CAAGCA-----A...AA.CTC...A-.--G**

**RN0AAB289YD03FM1**  **ATCA.AA....-CAC--G.CC.TC----G.G.A.--T..G..TC-GTAA..AT...-----C..-----AT.A----.CCA...--...A----CCGGA.A.AG.---ATC.T..T--ACTCAGC.....C....................TG-A---A.AT-TAT...CCAAGCA-----A...AA.CTC...A-.--G**

**RN0AAA727YI23RM1**  **ATCA..A....-CAT--G.CC.TC----..G.A.--T..G..TC-GTAA...TGG.-----...-----A..G----ACCA...--...A----CCG.T.A.AA.---ATC.T..T--ACTCAGC.....C....................TG-.---A.AT-TAT....CAAGCA-----A...AA-CTC..TA-.--G**

**RN0AAA90YK10FM1**  **ATCA.AA....-CAC--G.CC.TC----G.G.A.--T.GG..TC-ATAAA.CTG..-----C..-----AT.C----.CCA...--...A----CCG.T.A.AG.---ATC.T..T--ACTCAGC.....C....................TG-.---A.AT-TAT....CAAGCA-----A...A-.CTC...A-.--G**

**RN0AAB20YB17AHM1**  **ATCA.AA....-CAC--G.CC.TC----G.G.A.--T..G..TC-ATAAA.CTG..-----C..-----AT.C----.CCA...--...A----CCG.T.A.AG.---ATC.T..T--ACTCAGC.....C....................TG-.---A.AT-TAT....CAAGCA-----A...A-.CTC...A-.--G**

**RN0AAB577YG05AHM1** **ATCA.AA....-CAC--G.CC.TC----G.G.A.--T.GG..TC-ATAAA.CTG..-----C..-----AT.C----.CCA...--...A----CCG.T.A.AG.---ATC.T..T--ACTCAGC.....C....................TG-.---A.AT-TAT....CAAGCA-----A...A-.CTC..GA-.--G**

**RN0AAB48YA06AHM1**  **ATCA.AA....-CAC--G.CC.TC----G.G.A.--T.GG..TC-ATAAA.CTG..-----C..-----AT.C----.CCA...--...A----CCG.T.A.AG.---ATC.T..T--ACTCAGC.....C....................TG-.---A.AT-TAT....CAAGCA-----A...A-.CTC..GA-.--G**

**RN0AAA133YD03FM1**  **ATCA.AA....-CAC--G.CC.TC----G.G.A.--T..G..TC-ATAAA.CTG..-----C..-----AT.C----.CCA...--...A----CCG.A.A.AG.---ATC.T..T--ACTCAGC.....C....................TG-.---A.AT-TAT....CAAGCA-----A...A-.CTC...A-.--G**

**RN0AAA542YE07RM1**  **ATCA.AA....-CAC--G.CC.TC----G.G.A.--T..G..TC-ATAAA.CTG..-----C..-----AT.C----.CCA...--...A----CCG.A.A.AG.---ATC.T..T--ACTCAGC.....C....................TG-.---A.AT-TAT....CAAGCA-----A...A-.CTC...A-.--G**

**RN0AAA601YB08RM1**  **ATCA.AA....-CAC--G.CC.TC----G.G.A.--T..G..TC-ATAAA.CTG..-----C..-----AT.C----.CCA...--...A----CCG.A.A.AG.---ATC.T..T--ACTCAGC.....C....................TG-.---A.AT-TAT....CAAGCA-----A...A-.CTC...A-.--G**

**RN0AAA281YH13FM1**  **ATCA.AA....-CAC--G.CC.TC----G.G.A.--T..G..TC-ATAAA.CTG..-----C..-----AT.C----.CCA...--...A----CCG.A.A.AG.---ATC.T..T--ACTCAGC.....C...............A...--------------------------------------------------**

**RN0AAA699YB04FM1**  **ATCA.AA....-CAT--G.CC.C.----G.A.A.--T..G..TC-ATAA...TG..-----C.A-----A..C----.CCA...--...A----CCG.T.A.GG.---ATC.A..T--ACCCAAC...G.C...................C-G-.---A.---TATCA..GA.GTC-----A..G.--TCCC..A-.---**

**RN0AAA437YA19RM1**  **.GCA.AA....-------..CTTC----..GAA.--TCTG..TC-ATAA..CTG..-----C-------A..G----ATC....--...A----TCG.T.A.AG.---ATC.T...--ACTCAGC.....A....T........-.....AT.------.AT-TAT.AA.CAAG.A-----A...G--CTCG..A-.--G**

**RN0AAA499YO02FM1**  **.GCA.AA....-------..CTTC----..GAA.--TCTG..TC-ATAA..CTG..-----C-------A..G----ATC....--...A----TCG.T.A.AG.---ATC.T...--ACTCAGC.....A....T........-.....AT.------.AT-TAT.AA.CAAG.A-----A...G--CTCG..A-.--G**

**RN0AAA377YF05FM1**  **.GCA.AA....-------..CTTC----..GAA.--TCTG..TC-ATAA..CTG..-----C-------A..G----ATC....--...A----TCG.T.A.AG.---ATC.T...--ACTCAGC.....A....T........-.....AT.------.AT-TAT.AA.CAAG.A-----A...G--CTCG..A-.--G**

**RN0AAA222YD17RM1**  **A..TAA-...TTTCC-A.C.T.CGT---T.-AT..CTGGTG..-CA--T.CA.C..-----G.AAATTAA...GAAAA.CA.T.AC..------GGCTTTT.------TT..TA...T------T.TT-.AC....................GAACTT------CT..C.CAA.TTT----A..T...C-G.--AT..T.**

**RN0AAB173YC20AHM1** **A..TAA-...TGTCC-A.C.T.CGT---T.-AT..CTGGTG..-CA--T.CA.C..-----G.AAATTAA...GAAAA.CA.T.AC..------GGCTTTT.------TT..TA...T------T.TT-.AC....................GAACTT------CT..C.CAA.TTT----A..T...C-G.--AT..T.**

**RN0AAB161YD24FM1**  **A..TAA-...TTTCC-A.C.T.CGT---T.-AT..GTGGTG..-CA--T.CA.C..-----G.AAATTAA...GAAAA.CA.T.AC..------GGCTTTT.------TT..TA...T------T.TT-.AC....................GAACTT------CT..C.CAA.TTT----A..T...T-G.--AT..T.**

**RN0AAB55YM18AHM1**  **A..TAA-...TGTCC-A.C.T.CGT---T.-AT..CTGGTG..-CA--T.CA.C..-----G.AAATTAA...GAAAA.CA.T.AC..------GGCTTTT.------TT..TA...T------T.TT-.AC....................GAACTT------CT..C.CAA.TTT----A..T...C-G.--AT..T.**

**RN0AAA481YC13RM1**  **A..TAA-...TTTCC-A.C.T.CGT---T.-AT..CTGGTG..-CA--T.CA.C..-----G.AAATTAA...GAAAA.CA.T.AC..------GGCTTTT.------TT..TA...T------T.TT-.AC....................GAACTT------CT..C.CAA.TTT----A..T...C-G.--AT..T.**

**RN0AAA81YK04RM1**  **A..TAA-...TTTCC-A.C.T.CGT---T.-AT..CTGGTG..-CA--T.CA.C..-----G.AAATTAA...GAAAA.CA.T.AC..------GGCTTTT.------TT..TA...T------T.TT-.AC....................GAACTT------CT..C.CAA.TTT----A..TC..C-G.--AT..T.**

**RN0AAA229YJ17FM1**  **A..TAA-...TTTCC-A.C.T.CGT---T.-AT..CTGGTG..-CA--T.CA.C..-----G.AAATTAA...GAAAA.CA.T.AC..------GGCTTTT.------TT..TA...T------T.TT-.AC....................GAACTT.------G..C.CAA.TTT----A......C-G.--AT..T.**

**RN0AAA222YD17FM1**  **A..TAA-...TTTCC-A.C.T.CGT---T.-AT..CTGGTG..-CA--T.CA.C..-----G.AAATTAA...GAAAA.CA.T.AC..------GGCTTTT.------TT..TA...T------T.TT-.AC....................GAACTT.------T..C.CAA.TTT----A..T...C-G.--AT..T.**

**RN0AAA303YG05RM1**  **A..TAA-...TTTCC-A.C.T.CGT---T.-AT..CTGGTG..-CA--T.CA.C..-----G.AAATTAA...GAAAA.CA.T.AC..------GGCTTTT.------TT..TA...T------T.TT-.AC....................GAACTT------CT..C.CAA.TTT----A..T...T-G.--AT..T.**

**RN0AAA32YB09FM1**  **A..TAA-...TTTCC-A.C.T.CGT---T.-AT..CTGGTG..-CA--T.CA.C..-----G.AAATTAA...GAAAA.CA.T.AC..------GGCTTTT.------TT..TA...T------T.TT-.AC..................C.GAACTT.------T..C.CCG.TTC----A..T...T-G.--AT..T.**

**RN0AAA474YI20RM1**  **A..TAA-...TTTCC-A.C.T.CGT---T.-AT..CTGGTG..-CA--T.CA.C..-----G.AAATTAA...GAAAA.CA.T.AC..------GGCTTTT.------TT..TA...T------T.TT-.AC....................GAACTT.------T..C.CAA.TTT----A..T...C-G.--AT..T.**

**RN0AAA373YI20FM1**  **--------..TGTCC-A.C.T.CGT---T.-AT..CTGGTG..-CA--T.CA.C..-----G.AAATTATT..GAAAA.CA.T.AC..------GGCTTTT.------TT..TA...T------T.TT-.AC....................GAACTT.------T..C.CAA.TTT----A..TA..C-G.--AT..T.**

**RN0AAA434YP23FM1**  **A..TAA-...TTTCC-A.C.T.CGT---T.-AT..CTGGTG..-CA--T.CA.C..-----G.AAATTAA...GAAAA.CA.T.AC..------GGCTTTT.------TT..TA...T------T.TT-.AC....................GAACTT.------T..C.CAA.TTT----A..T..CC-G.--AT..T.**

**RN0AAA462YN08FM1**  **A..TAA-...TTTCC-A.C.T.CGT---T.-AT..CTGGTG..-CA--T.CG.C..-----G.AAATTAA...GAAAA.CA.T.AC..------GGCTTTT.------AT..TA...T------T.TT-.AC........C...........CAACTT.------T..C.CAA.TTT----A..T...C-G.--AT..T.**

**RN0AAA474YG15FM1**  **A..TAA-...TTTCC-A.C.T.CGT---T.-AT..CTGGTG..-CA--T.CA.C..-----G.AAATTAA...GAAAA.CA.T.AC..------GGCTTTT.------TT..TA...T------T.TT-.AC....................GAACTT------CT..C.CAA.TTT----A..T...T-G.--AT..T.**

**RN0AAA481YC13FM1**  **A..TAA-...TGTCC-A.C.T.CGT---T.-AT..CT.GTG..-CA--T.CA.G..-----G.AAATTAA...GAAAA.CA.T.AC..------GGCTTTT.------TT..TA...T------T.TT-.AC....................GAACTT.------T..C.CAG.TTT----A..T...C-G.--AT..T.**

**RN0AAA556YI14FM1**  **A..TAA-...TTTCC-A.C.T.CGT---T.-AT..CTGGTG..-CG--T.CC.C..-----G.AACTTAA...GAAAA.CA.T.AC..------GGCTTTT.------TT..TA...T------T.TT-.AC......G.............GAACTT.------T..CGCAA.TTT----A..T...C-G.--AT..T.**

**RN0AAA76YA17RM1**  **A..TAA-...TGTCC-A.C.T.CGG---T.-AT..CTGGTG..-CA--T.CA.C..-----G.AAATTAA...GAAAA.CA.T.AC..------GGCTTTT.------TT..TA...T------T.TT-.AC....................GAACTT------CT..C.CAA.TTT----A..T...C-G.--AT..T.**

**RN0AAA94YM05RM1**  **A..TAA-...TTTCC-A.C.T.CGT---T.-AT..CT.GTG..-CAG-T.CA.C..-----G.AAATAAA...GAAAA.CA.T.AC..------GGCTTTT.------TT..TA..CT------T.TT-..C....................GAACTT.------T..C.CAA.TTT----A..T...C-G.--AT.CT.**

**RN0AAB55YM18FM1**  **A..TAA-...TTTCC-A.C.T.CGT---T.-AT..CTGGTG..-CA--T.CA.C..-----G.AAATTAA...GAAAA.CA.T.AC..------GGCTTTT.------TT..TA...T------T.TT-.AC....................GAACTT.------T..C.CAA.TTT----A..T...C-G.--AT..T.**

**RN0AAB56YP24FM1**  **A..TAA-...TTTCC-C.C.T.CGT---T.-AT..CTGGTG..-CA--T.CA.C..-----G.AAATTAA...GAAAA.CA.T.AC..------GGCTTTT.------TT..TA...T------T.TT-.AC....................GAACTT.------T..C.CAA.TTT----A..T...C-G.--AT..T.**

**RN0AAA513YA20RM1**  **---------------------------------------------------------------------------AAA.CA.T.AC..------GGCTTTT.------TT..TA...T------T.TT-.AC....................GAACTT.------T..C.CAA.TTT----A..T...C-G.--AT..CT**

**Box 1**  **-----------------------------------------------------------------------------------------------------------------------------------.................----------------------------------------------------**

610 620 630 640 650 660 670 680 690 700 710 720 730 740 750 760 770 780 790 800

....|....|....|....|....|....|....|....|....|....|....|....|....|....|....|....|....|....|....|....|....|....|....|....|....|....|....|....|....|....|....|....|....|....|....|....|....|....|....|....|

**RN0AAB125YE10FM1**  **A----AGGAAAAAG------------------------------------------------------------------------------------------------------------------------------------------------------------------------------------------**

**RN0AAB3YE08AHM1**  **.----.........------------------------------------------------------------------------------------------------------------------------------------------------------------------------------------------**

**RN0AAB140YN05FM1**  **.----.........------------------------------------------------------------------------------------------------------------------------------------------------------------------------------------------**

**RN0AAB414YN09FM1**  **.----.........------------------------------------------------------------------------------------------------------------------------------------------------------------------------------------------**

**RN0AAB545YM04AHM1** **.----.........------------------------------------------------------------------------------------------------------------------------------------------------------------------------------------------**

**RN0AAA130YM16FM1**  **.----.........------------------------------------------------------------------------------------------------------------------------------------------------------------------------------------------**

**RN0AAA519YK05RM1**  **.----.........------------------------------------------------------------------------------------------------------------------------------------------------------------------------------------------**

**RN0AAB125YE10FM1**  **.----.........------------------------------------------------------------------------------------------------------------------------------------------------------------------------------------------**

**RN0AAB3YE08AHM1**  **.----.........------------------------------------------------------------------------------------------------------------------------------------------------------------------------------------------**

**RN0AAB140YN05FM1**  **.----.........------------------------------------------------------------------------------------------------------------------------------------------------------------------------------------------**

**RN0AAB414YN09FM1**  **.----.........------------------------------------------------------------------------------------------------------------------------------------------------------------------------------------------**

**RN0AAB545YM04AHM1** **.----.........------------------------------------------------------------------------------------------------------------------------------------------------------------------------------------------**

**RN0AAA130YM16FM1**  **.----.........------------------------------------------------------------------------------------------------------------------------------------------------------------------------------------------**

**RN0AAA519YK05RM1**  **.----.........------------------------------------------------------------------------------------------------------------------------------------------------------------------------------------------**

**RN0AAB176YD12FM1**  **.----........A------------------------------------------------------------------------------------------------------------------------------------------------------------------------------------------**

**RN0AAA286YA16FM1**  **.----.A....C.A------------------------------------------------------------------------------------------------------------------------------------------------------------------------------------------**

**RN0AAA179YC21RM1**  **.CAT-.AA....G.------------------------------------------------------------------------------------------------------------------------------------------------------------------------------------------**

**RN0AAA756YE07FM1**  **.CAT-.AA....G.------------------------------------------------------------------------------------------------------------------------------------------------------------------------------------------**

**RN0AAA591YF08RM1**  **G----........AA-----------------------------------------------------------------------------------------------------------------------------------------------------------------------------------------**

**RN0AAA538YB16RM1**  **.----........AA-----------------------------------------------------------------------------------------------------------------------------------------------------------------------------------------**

**RN0AAB458YP20AHM1** **.----........AA-----------------------------------------------------------------------------------------------------------------------------------------------------------------------------------------**

**RN0AAA669YI24RM1**  **.----........A------------------------------------------------------------------------------------------------------------------------------------------------------------------------------------------**

**RN0AAA87YA19FM1**  **.----........AT-----------------------------------------------------------------------------------------------------------------------------------------------------------------------------------------**

**RN0AAB380YP14FM1**  **.----.......G.G-----------------------------------------------------------------------------------------------------------------------------------------------------------------------------------------**

**RN0AAB423YM10FM1**  **.----.......G.G-----------------------------------------------------------------------------------------------------------------------------------------------------------------------------------------**

**RN0AAB147YJ10FM1**  **GG----..GGGGCTT-----------------------------------------------------------------------------------------------------------------------------------------------------------------------------------------**

**RN0AAA64YH05FM1**  **.----.........------------------------------------------------------------------------------------------------------------------------------------------------------------------------------------------**

**RN0AAB176YD12FM1**  **.----........A------------------------------------------------------------------------------------------------------------------------------------------------------------------------------------------**

**RN0AAA286YA16FM1**  **.----.A....C.A------------------------------------------------------------------------------------------------------------------------------------------------------------------------------------------**

**RN0AAA179YC21RM1**  **.CAT-.AA....G.------------------------------------------------------------------------------------------------------------------------------------------------------------------------------------------**

**RN0AAA756YE07FM1**  **.CAT-.AA....G.------------------------------------------------------------------------------------------------------------------------------------------------------------------------------------------**

**RN0AAA591YF08RM1**  **G----........AA-----------------------------------------------------------------------------------------------------------------------------------------------------------------------------------------**

**RN0AAA538YB16RM1**  **.----........AA-----------------------------------------------------------------------------------------------------------------------------------------------------------------------------------------**

**RN0AAB458YP20AHM1** **.----........AAA----------------------------------------------------------------------------------------------------------------------------------------------------------------------------------------**

**RN0AAA669YI24RM1**  **.----........A------------------------------------------------------------------------------------------------------------------------------------------------------------------------------------------**

**RN0AAA87YA19FM1**  **.----........ATGA---------------------------------------------------------------------------------------------------------------------------------------------------------------------------------------**

**RN0AAB380YP14FM1**  **.----.......G.G-----------------------------------------------------------------------------------------------------------------------------------------------------------------------------------------**

**RN0AAB423YM10FM1**  **.----.......G.G-----------------------------------------------------------------------------------------------------------------------------------------------------------------------------------------**

**RN0AAB147YJ10FM1**  **G----G..GGGGCTT-----------------------------------------------------------------------------------------------------------------------------------------------------------------------------------------**

**RN0AAA64YH05FM1**  **---A-.........------------------------------------------------------------------------------------------------------------------------------------------------------------------------------------------**

**RN0AAB311YB19AHM1** **GT-C-...G...C.AT----------------------------------------------------------------------------------------------------------------------------------------------------------------------------------------**

**RN0AAB44YH23AHM1**  **GT-C-...GG..TAGG----------------------------------------------------------------------------------------------------------------------------------------------------------------------------------------**

**RN0AAA717YI15RM1**  **GT-C-...GG..TAG-----------------------------------------------------------------------------------------------------------------------------------------------------------------------------------------**

**RN0AAB125YA16FM1**  **GT-C-...G...C.G-----------------------------------------------------------------------------------------------------------------------------------------------------------------------------------------**

**RN0AAB437YE21AHM1** **GT-C-...G...C.G-----------------------------------------------------------------------------------------------------------------------------------------------------------------------------------------**

**RN0AAB266YH16AHM1** **GT-C-...G...C.G-----------------------------------------------------------------------------------------------------------------------------------------------------------------------------------------**

**RN0AAA131YF02RM1**  **GT-C-...G...C.G-----------------------------------------------------------------------------------------------------------------------------------------------------------------------------------------**

**RN0AAA130YE05RM1**  **GT-C-...G...CCG-----------------------------------------------------------------------------------------------------------------------------------------------------------------------------------------**

**RN0AAA579YK24RM1**  **GT-T-...G...C-------------------------------------------------------------------------------------------------------------------------------------------------------------------------------------------**

**RN0AAB100YP08AHM1** **GT-C-...G...C.G-----------------------------------------------------------------------------------------------------------------------------------------------------------------------------------------**

**RN0AAB180YL08FM1**  **GT-C-...G...C.G-----------------------------------------------------------------------------------------------------------------------------------------------------------------------------------------**

**RN0AAA175YL12RM1**  **--------------------------------------------------------------------------------------------------------------------------------------------------------------------------------------------------------**

**RN0AAB484YE18FM1**  **--------------------------------------------------------------------------------------------------------------------------------------------------------------------------------------------------------**

**RN0AAA316YE18RM1**  **--------------------------------------------------------------------------------------------------------------------------------------------------------------------------------------------------------**

**RN0AAA14YA24RM1**  **GT-C-...G...C.G-----------------------------------------------------------------------------------------------------------------------------------------------------------------------------------------**

**RN0AAA691YK01RM1**  **GT-C-...G..GCAA-----------------------------------------------------------------------------------------------------------------------------------------------------------------------------------------**

**RN0AAB526YI05AHM1** **--------------------------------------------------------------------------------------------------------------------------------------------------------------------------------------------------------**

**RN0AAB490YN21AHM1** **GT-C-...G...CAT-----------------------------------------------------------------------------------------------------------------------------------------------------------------------------------------**

**RN0AAA132YI06RM1**  **GT-C-...C...CAT-----------------------------------------------------------------------------------------------------------------------------------------------------------------------------------------**

**RN0AAB289YD03FM1**  **GT-C-..AGG..CAG-----------------------------------------------------------------------------------------------------------------------------------------------------------------------------------------**

**RN0AAA727YI23RM1**  **GT-C-...TG..CAG-----------------------------------------------------------------------------------------------------------------------------------------------------------------------------------------**

**RN0AAA90YK10FM1**  **GT-C-...GG..TAG-----------------------------------------------------------------------------------------------------------------------------------------------------------------------------------------**

**RN0AAB20YB17AHM1**  **GT-C-...GG..TAG-----------------------------------------------------------------------------------------------------------------------------------------------------------------------------------------**

**RN0AAB577YG05AHM1** **GT-C-...GG..TAG-----------------------------------------------------------------------------------------------------------------------------------------------------------------------------------------**

**RN0AAB48YA06AHM1**  **GT-C-...CG..TAG-----------------------------------------------------------------------------------------------------------------------------------------------------------------------------------------**

**RN0AAA133YD03FM1**  **GT-C-...GG..TAG-----------------------------------------------------------------------------------------------------------------------------------------------------------------------------------------**

**RN0AAA542YE07RM1**  **GT-C-...GG..TAG-----------------------------------------------------------------------------------------------------------------------------------------------------------------------------------------**

**RN0AAA601YB08RM1**  **GT-C-...GG..TAG-----------------------------------------------------------------------------------------------------------------------------------------------------------------------------------------**

**RN0AAA281YH13FM1**  **--------------------------------------------------------------------------------------------------------------------------------------------------------------------------------------------------------**

**RN0AAA699YB04FM1**  **-TTC--TCTGG..CT-----------------------------------------------------------------------------------------------------------------------------------------------------------------------------------------**

**RN0AAA437YA19RM1**  **.T-C-...G...C-------------------------------------------------------------------------------------------------------------------------------------------------------------------------------------------**

**RN0AAA499YO02FM1**  **.T-C-...G...C-------------------------------------------------------------------------------------------------------------------------------------------------------------------------------------------**

**RN0AAA377YF05FM1**  **.T-C-...G...C-------------------------------------------------------------------------------------------------------------------------------------------------------------------------------------------**

**RN0AAA222YD17RM1**  **.ATT-.TCC...TATTT----CATGTAATT------AAAAATTTCCA--ACTTACGTTAATTCCTGGTG-TACATTCAACT----TGAAAAT----TAAATTGAAAAACAATTACCTGGCTTTTTTTGATACAGTTTTTA---ACGCTCTTTCGAATGATATATAGAACTTCTATCTCAAATTTAAATTCGC-GAATTAT**

**RN0AAB173YC20AHM1** **.ATT-.TAC....ATTT----CATGTAATT------AAAAATGTCCA--ACTTACGTTAATTCCTTGTG-TACATTCAAGT----TGAAAAT----TAAATTGAAAAACAATTACCTGGCTTTTTTTGATACAGTTTTTA---ACGCTCTTTCGAATGATATATAGAACTTCTATCTCAAATTTAAATTCGC-GAATTAT**

**RN0AAB161YD24FM1**  **.ATT-.TAC....ATTT----CATGTAATT------AAAAATTTCCA--ACTTACGTTAATTCCTGGTG-TACATTCAACT----TGAAAAT----TAAATTGAAAAACAATTACCTGGCTTTTTTTGATACAGTTTTTA---ACGCTCTTTCGAATGATATATAGAACTTCTATCTCAAATTTAAATTCGT-GAATTAT**

**RN0AAB55YM18AHM1**  **.ATT-.TAC...TATTT----CATGTAATT------AAAAATTTCCA--ACTTACGTTAATTCCTGGTG-TACATTCAACT----TGAAAAT----TAAATTGAAAAACAATTACCTGGCTTTTTTTGATACAGTTTTTA---ACGCTCTTTCGAATGATATATAGAACTTCTATCTCAAATTTAAATTCGC-GAATTAT**

**RN0AAA481YC13RM1**  **.ATT-.TAC....ATTT----CATGTAATT------AAAAATGTCCA--ACTTACGTTAATTCCTTGTG-TACATTCAAGT----TGAAAAT----TAAATTGAAAAACAATTACCTGGCTTTTTTTGATACAGTTTTTA---ACGCTCTTTCGAATGATATATAGAACTTCTATCTCAAATTTAAATTCGC-GAATTAT**

**RN0AAA81YK04RM1**  **.ATT-.CAC....ATTT----CATGTAATT------AAAAATTTCCA--ACTTACGTTAATTCCTGGTG-TACATTCAACT----TGAAAAT----TAAATTGAAAAACAATTACCTGGCTTTTTTTGATACAGTTTTTA---ACGCTCTTTCGAATGATATATAGAACTTCTCTCTCAAATTTAAATACGC-GAATTAT**

**RN0AAA229YJ17FM1**  **.ATT-.TAC-...TATT---TCATGTAATT------AAAAATTTCCA--ACTTACGTTAATTCCTGGTG-TACATTCAACTTG--AA--AAT----TAAATTGAAAAACAATTACCTGGCTTTTTTTGATACAGTTTTTA---ACGCTCTTTCGAATGCTTTCTAGCACATCGGTCGCCGTTTTAAATACGCTACTAACT**

**RN0AAA222YD17FM1**  **.ATT-.TAC-...AATT---TCATGTAATT------AAAAATTTCCA--ACTTACGTTAATTCCTGGTG-TACATTCAACTTG--AA--AAT----TAAATTGAAAAACAATTACCTGGCTTTTTTTGATACAGGTTTTA---ACGCTCTTTCTAATGATATATAGA---------------------------------**

**RN0AAA303YG05RM1**  **.ATT-.TAC...TATTT----CATGTAATT------AAAAATTTCCA--ACTTACGTTAATTCCTGGTG-TACATTCAACT----TGAAAAT----TAAATTGAAAAACAATTACCTGGCTTTTTTTGATACAGTTTTTA---ACGCTCTTTCGAATGATATATAGAACTTCTATCTCAAATTTAAATTCGC-GAATTAT**

**RN0AAA32YB09FM1**  **.ATT-.CCC-.GCTA-----------------------------------------------------------------------------------------------------------------------------------------------------------------------------------------**

**RN0AAA474YI20RM1**  **.ATT-.TAC-...TATT---TCATGTAATT------AAAAATTTCCA--ACTTACGTTAATTCCTGGTG-TACATTCAACTTG--AA--AAT----TAAATTGAAAAACAATTACCTGGCTTTTTTTGATACAGTTTTTA---ACGCTCTTTCGAATGATATA-------------------------------------**

**RN0AAA373YI20FM1**  **.ATT-.TAC-...AATT---TCATGTAATT------AAAAATGTCCA--ACTTACCTTTCTTGCAGGCG-GGCGGTCGATATG--AA--TGT----ATAATAGATCAGCAATTAC-------------------------------------------------------------------------------------**

**RN0AAA434YP23FM1**  **.ATT-.TAC-C..AATT---TCATGTAATT------AAAAATTTCCA--ACTTACGTTAATTCCTGGTG-TACATTCAACTTG--AA--AAT----TAAATTGAAAAACAATTACCTGGCTTTTTTTGATACAGTTTTTA---ACGCTCTTTCGAATGATATATAGAACTTCTATCTCAAATTTAAATTCGCGAATTATA**

**RN0AAA462YN08FM1**  **.ATT-.TAC-...TATT---TCATGTAATT------AAAAATGTCCA--ACTTACGTTAATTCCTTGTG-TACATTCAACTTG--AA--GAT----TATA----------------------------------------------------------------------------------------------------**

**RN0AAA474YG15FM1**  **.ATT-.TAC...TATTT----CATGTAATT------AAAAATTTCCA--ACTTACGTTAATTCCTGGTG-TACATTCAACT----TGAAAAT----TAAATTGAAAAACAATTACCTGGCTTTTTTTGATACAGTTTTTA---AAGCTCTTTCGAATGATATATAGAACTTCTATCTCAAATTTAAATTCGC--------**

**RN0AAA481YC13FM1**  **.ATT-.TAC-...AATT---TCATGTAATT------AAAAATTTCCA--ACTTACGTTAATTCCTGGTG-TACATTCAACTTG--AA--AAT----TAAATTGAAAAACAATTACCTGGCTTTTTTTGATACAGTTTTTA---ACGCTCTTTCGAATGATATATAGAACTTCTATCTCAAATTTAGATTCGAGAATTATA**

**RN0AAA556YI14FM1**  **.ATT-.TAC-...AATT---TCATGTAAGT------AAAAATTTCCA--AC-----------------------------------------------------------------------------------------------------------------------------------------------------**

**RN0AAA76YA17RM1**  **.ATT-.TAC....ATTT----CATGTAATT------AAAAATTTCCA--ACTTACGTTAATTCCTTGTG-TACATTCAACT----TGAAAAT----TAAATTGAAAAACAATTACCTGGCTTTTTTTGATACAGTTTTTA---ACGCTCTTTCGAATGATATATAG----------------------------------**

**RN0AAA94YM05RM1**  **.ATT-.TA------------------------------------------------------------------------------------------------------------------------------------------------------------------------------------------------**

**RN0AAB55YM18FM1**  **.ATT-.TAC-...AATT---TCATGTAATT------AAAAATTTCCA--ACTTACGTTAATTCCTGGTG-TACATTCAACTTG--AA--AAT----TAAATTGAAAAACAATTACCTGGCTTTTTTTGATACAGTTTTTA---ACGCTCTTTCGAATGATATATAGAACTTCTATCTCAAATTTAAATTCGCGAATTATA**

**RN0AAB56YP24FM1**  **.ATT-.TAC-...TATT---TCATGTAATT------AAAAATTTCCA--ACTTACGTTAATTCCCGGTGGTACATTCAACTTG--AA--AATA---TAAATTGAAAAACAATTACCTGGCTTTTTTTGATACCGTT----------------------------------------------------------------**

**RN0AAA513YA20RM1**  **TATT-.TAC-...TATT---TCATGTAACT------GCCAATTT------------------------------------------------------------------------------------------------------------------------------------------------------------**

**Box 1**  **--------------------------------------------------------------------------------------------------------------------------------------------------------------------------------------------------------**

Figure S4. Alignment of Box 1-containing sequences from unassembled reads of *M. incogita* sequenced genome. Sequences are compared with first sequence and positions identical to the first sequence are shown with dot. Sequences are deposited in EMBL data bank under accession numbers: KC968979 - KC969073. Box 1 is shaded with yellow.
