# Supplementary material for: Conserved DNA Motifs, Including the CENP-B Box-like, Are Possible Promoters of Satellite DNA Array Rearrangements in Nematodes
Source: PLoS One. 2013 Jun 27;8(6):e67328. doi: 10.1371/journal.pone.0067328 (PMC3694981; doi:10.1371/journal.pone.0067328)
Supplement: Figure S5 — Alignment of Box 2 from HOR related monomers of group 1 (1aH, 1bH, 1b’H1c, and 1dH). (DOC) [file pone.0067328.s005.doc]

10 20

....|....|....|....|

**1aHfa2**  **TTCCTTCCCCCAAATTTTTC**

**1aHfa8**  **TTCCTTCCCCCAAATTTTTC**

**1aHfa17**  **TTCCTTCCCCCAAATTTTTC**

**1aHfaN4** **TTCCTTCCCCCAAATTTTTC**

**1aHfaP1**  **TTCCTTCCCCCAAATTTTTC**

**1aHfaP4**  **TTCCTTCCCCCAAATTTTTC**

**1aHfaP10**  **TTCCTTCCCCCAAATTTTTC**

1aH

**1aHch23**  **TTCCT-CCCCCAAATTTTTC**

**1aHch5**  **TTCCTTCCCCCAAATTTTTC**

**1aHch3**  **TTCCT-CCCCCAAATTTTTC**

**1aHch8**  **TTCCT-CCCCCAAATTTTTC**

**1aHfaP8**  **TTCCTTCCCCAAAATTTTTC**

**1aHchP9**  **TTCCTTCCCCAAAATTTTTC**

**1aHfa18**  **TTCCTTCCCCAAAATTTTTC**

**1aHch21**  **TTCCTTCCCCCAAATTTTTC**

**1aHch12**  **TTCCTTCCCCCAAATTTTTC**

**1aHch11a**  **TTCCTTCCCCCAAATTTTTC**

**1aHch21a**  **TTCCTTCCCCCAAATTTTTC**

**1aHch4**  **TTCCTTCCCCCAAATTTTTC**

**1aHch6**  **TTCCTTCCCCCAAATTTTTC**

**1aHch22**  **TTCCTTC-CCCCAATTTTTC**

**1aHch22a**  **TTCCTTC-CCCCAATTTTTC**

**1aHch9**  **TTCCT-CCCCCAAATTTTTC**

**1aHch11**  **TTCCTTCCCCCAAATTTTTC**

**1aHch2**  **TTCCT-CCCCCAAATTTTTC**

**1bHch2**  **TTCCTTCCCCCAAAAAATTC**

**1bHch8**  **TTCCTTCCCCCAAAAAATTC**

**1bHch3**  **TTCCTTCACCCCCAAAAAAT**

**1bHch22**  **NTCCTTCACCCCCAAAAAAC**

**1bHch4**  **TTCCTTCACCCCCAAAAAAT**

**1bHch12**  **TTCCTTCACCCCCAAAAAAT**

1b'H

**1bHch11**  **TTCCTTCACCCCCAAAAAAT**

**1bHch21**  **TTCCTTCACCCCCAAAAAAT**

**1bHfa2**  **TTCCTTC-CCCCCAAAAAAT**

**1bHch5**  **TTCTTTCACCCCCAAAAAAT**

**1bHch6**  **TTCCTTCACCCCCAAAAAAT**

**1bHch9**  **TTCCTTCCCCCAAAAAATTC**

**1bHch23**  **TTCCTTCCCCCAAAAAATTC**

**1bHfaN4b**  **TTCCTTCCCCCAAAAAATTC**

**1bHfaP1b**  **TTCCTTCCCCCAAAAAATTC**

**1bHfa4b**  **TTCCTTCCCCCAAAAAATTC**

**1bHfaP10b**  **TTCCTTCCCCCAAAAAATTC**

**1bHfa8b**  **TTCCTTCCCCCAAAAAATTC**

**1bHchP9b**  **TTCCTTCCCCCAAAAAATTC**

**1bHfaN4**  **TTCCTTCTCCCCAAAATTTT**

**1bHfa8**  **TTCCTTCTCCCCAAAATTTT**

1bH

**1bHfaP1**  **TTCCTTCTCCCCAAAATTTT**

**1bHfaP4**  **TTCCTTCTCCCCAAAATTTT**

**1bHfaP8**  **TTCCTTCTCCCCAAAATTTT**

**1bHchP9**  **TTCCTTCTCCCCAAAATTTT**

**1bHfa2a**  **TTCCTTCTCCCCAAAATTTT**

**1bHfa17**  **TTCCTTCTCCCCAAAATTTT**

**1bHfaP10**  **TTCCTTCTCCCCAAAATTTT**

**1cHch6**  **ATTCTCCCCCCAAAAAATTT**

**1cHfaP1**  **ATTCTCCCCCCAAAAAATTT**

**1cHfaP4**  **ATTCTCCCCCCAAAAAATTT**

1cH

**1cMfa16b**  **ATTCTCCCCCCAAAAAATTT**

**1cHfaP8**  **ATTCTCCCCCCAAAAAATTT**

**1cHch2**  **ATTCTCCCCCCAAAAAATTT**

**1cHch9**  **ATTCTCCCCCCAAAAAATTT**

**1cHch22**  **ATTCTCCCCCCAAAAAATTT**

**1cHch23**  **ATTCTCCCCCCAAAAAATTT**

**1cHfaN4**  **ATTCTCCCCCCAAAAAATTT**

**1cHchP9**  **ATTCTCCCCCCAAAAAATTT**

**1cHfaP10**  **ATTCTCCCCCCAAAAAATTT**

**1cHch4**  **ATTCTCCCCCCAAAAAATTT**

**1cHch8**  **ATTCTCCCCCCAAAAAATTT**

**1cHch12**  **ATTCTCCCCCCAAAAAATTT**

**1cHch21**  **ATTCTCCCCCCAAAAAATTT**

**1cHch11**  **ATTCTCCCCCCAAAAAATTT**

**1cHch3**  **ATTCTCCCCCCAAAAAATTT**

**1dHch2**  **TTTTTTCCCCCAAAAAAATT**

**1dHch8**  **TTTTTTCCCCCAAAAAAATT**

**1dHch6**  **TTTTTTCCCCCAAAAAAATT**

**1dHch3**  **TTTTTTCCCCCAAAAAAATT**

**1dHch4**  **TTTTTTCCCCCAAAAAAATT**

**1dHch12**  **TTTTTTCCCCCAAAAAAATT**

**1dHch21**  **TTTTTTCCCCCAAAAAAATT**

1dH

**1dHch22**  **TTTTTTCCCCCAAAAAAATT**

**1dHch23**  **TTTTTTCCCCCAAAAAAATT**

**1dHch11**  **TTTTTTCCCCCAAAAAAATT**

**1dHfaN4**  **TTTTTTCCCCCAAAAAAATT**

**1dHfaP1**  **TTTTTTCCCCCAAAAAAATT**

**1dHfaP4**  **TTTTTTCCCCCAAAAAAATT**

**1dHfaP10**  **TTTTTTCCCCCAAAAAAATT**

**1dHch9**  **TTTTTTCCCCCAAAAAAATT**

**1dHch13a**  **TTTTTTCCCCCAAAAAAATT**

**1dHfaP8**  **TTTTTTCCCCCAAAAAAATT**

**1dHchP9**  **TTTTTTCCCCCAAAAAAATT**

Figure S5. Alignment of Box 2 from HOR related monomers of group 1 (1aH, 1bH, 1b’H1c, and 1dH).
