# Supplementary material for: Conserved DNA Motifs, Including the CENP-B Box-like, Are Possible Promoters of Satellite DNA Array Rearrangements in Nematodes
Source: PLoS One. 2013 Jun 27;8(6):e67328. doi: 10.1371/journal.pone.0067328 (PMC3694981; doi:10.1371/journal.pone.0067328)
Supplement: Table S1 — Primers used to amplify genomic sequences. (DOC) [file pone.0067328.s006.doc]

| Sequence name  Table S1. Primers used to amplify genomic sequences | Primer name | Primer sequence | Reference |
| --- | --- | --- | --- |
| 1a satDNA | 1aL  1aR | CCAAATTCAGCAAATTTCCAACGAT  AATCCATCGACTAGTTTTTGAG | This work |
| 1a satDNA (HOR specific) | 1a’L  1a’R | CCAAATTCAGCAAATTTCCAACGAT  GGGGAAGGAATATTTTTGAACTTTT | This work |
| 1b satDNA | 1bL  1bR | CATATCTCTCAAAGCCTTCT  TCGGAAGCATATTCGCTGT | This work |
| 1c satDNA | 1cL  1cR | TCGATTCACCTCTTCATCCTC  GGGGGGAGAATGGATACTTTG | This work |
| 2a satDNA | 2aL  2aR | CCTCTTTCGAATGATATATGAATC TTCAGTAAGTTATGAGACTTGTTCC | This work |
| 2b satDNA | 2bL  2bR | GGACTTATGAAATTGTAGGTCAGT  GCTCTTTCGAATGATATATGAATC | This work |
| U1 | U1L  U1R | GGTGTAGAAGAGACAAGCCTC  AGGGTGTTCCTTTACTCCTTC | This work |
| U2 | U2L  U2R | CTTGTTAGATATTTACAATTTTGG  ATTCCCATTCTATATAGATGATG | This work |
| *M. fallax* SCAR | Ff2  Rf | CCATTTCTGCTAAATGCCAAACTA  GGACACAGTAATTCATGAGCTAG | Zijistra, 2000 |
| *M. chitwoodi* SCAR | Fc2  Rc | GGCATTGACGTGCTCCGAGAGT  GGTCTGAGTGAGGACAAGAGTA | Zijistra, 2000 |
