# Supplementary material for: Conserved DNA Motifs, Including the CENP-B Box-like, Are Possible Promoters of Satellite DNA Array Rearrangements in Nematodes
Source: PLoS One. 2013 Jun 27;8(6):e67328. doi: 10.1371/journal.pone.0067328 (PMC3694981; doi:10.1371/journal.pone.0067328)
Supplement: Table S2 — Description of cloned satellite DNA arrays. In cloned satellite fragments, letters H, M and h indicate higher-order repeats, monomeric arrays, complex fragment, respectively. Then follow primer name (first subscript), species acronym and clone number (second subscript). (DOC) [file pone.0067328.s007.doc]

| primers | species | Satellite fragments | Length (bp) |
| --- | --- | --- | --- |
| 1c satDNA primers  (1cL and 1cR) | *M. fallax* | H_1c_fa_2_ | 1353 |
|  |  | H_1c_fa_8_ | 1588 |
|  |  | H_1c_fa_17_ | 1530 |
|  |  | H_1c_fa_18_ | 1445 |
|  |  | M_1c_fa_8_ | 1180 |
|  |  | M_1c_fa_11_ | 1040 |
|  |  | M_1c_fa_6_ | 505 |
|  |  | M_1c_fa_7_ | 505 |
|  | *M. chitwoodi* | H_1c_ch_2_ | 1480 |
|  |  | H_1c_ch_3_ | 1480 |
|  |  | H_1c_ch_4_ | 1480 |
|  |  | H_1c_ch_6_ | 1480 |
|  |  | H_1c_ch_8_ | 1480 |
|  |  | H_1c_ch_9_ | 1523 |
|  |  | H_1c_ch_11_ | 1200 |
|  |  | H_1c_ch_12_ | 1533 |
|  |  | M_1c_ch_13_ | 1036 |
|  |  | M_1c_ch_16_ | 1040 |
|  |  | M_1c_ch_10_ | 505 |
|  |  | M_1c_ch_5_ | 505 |
| U1 primers  (1A and 1B) | *M. fallax* | H_u_fa_4_ | 1422 |
|  |  | H_u_fa_1_ | 1419 |
|  |  | H_u_fa_7_ | 1419 |
|  |  | H_u_fa_8_ | 1252 |
|  |  | H_u_fa_9_ | 1253 |
|  |  | H_u_fa_10_ | 1419 |
|  |  | h_u_fa_1_ | 750 |
|  |  | h_u_fa_2_ | 750 |
|  |  | h_u_fa_3_ | 870 |
|  | *M. chitwoodi* | H_u_ch_11_ | 1264 |
|  |  | H_u_ch_21_ | 1269 |
|  |  | H_u_ch_22_ | 1268 |
|  |  | H_u_ch_23_ | 1266 |
|  |  | h_u_ch_1_ | 750 |
|  |  | h_u_ch_2_ | 750 |
|  |  | h_u_ch_3_ | 870 |
| 1a satDNA primers  (1aL and 1aR) | *M. fallax* | M_1a_fa_1_ | 910 |
|  |  | M_1a_fa_2_ | 903 |
|  |  | M_1a_ fa_3_ | 903 |
|  |  | M_1a_ fa_4_ | 901 |
|  |  | M_1a_ fa_6_ | 902 |
|  |  | M_1a_ fa_7_ | 906 |
|  | *M. chitwoodi* | M_1a_ ch_3_ | 941 |
|  |  | M_1a_ ch_4_ | 586 |
|  |  | M_1a_ ch_5_ | 587 |
|  |  | M_1a_ ch_6_ | 575 |
|  |  | M_1a_ ch_8_ | 914 |
|  |  | M_1a_ ch_9_ | 922 |
|  |  | M_1a_ ch_11_ | 911 |
|  |  | M_1a_ ch_13_ | 911 |
| 2a sat DNA primers  (2aL and 2aR) | *M. fallax* | M_2a_ fa_3_ | 801 |
|  |  | M_2a_ fa_4_ | 487 |
|  |  | M_2a_ fa_5_ | 487 |
|  |  | M_2a_ fa_7_ | 487 |
|  | *M. chitwoodi* | M_2a_ ch_1_ | 487 |
|  |  | M_2a_ ch_2_ | 487 |
|  |  | M_2a_ ch_3_ | 801 |
| 2b satDNA primers  (2bL and 2bR) | *M. chitwoodi* | M_2b_ ch_1_ | 500 |
|  |  | M_2b_ ch_2_ | 500 |

Table S2. Description of cloned satellite DNA arrays. In cloned satellite fragments, letters H, M and h indicate higher-order repeats, monomeric arrays, complex fragment, respectively. Then follow primer name (first subscript), species acronym and clone number (second subscript).
